# Meningitis Caused by the Live Varicella Vaccine Virus: Metagenomic Next Generation Sequencing, Immunology Exome Sequencing and Cytokine Multiplex Profiling

**DOI:** 10.3390/v13112286

**Published:** 2021-11-16

**Authors:** Prashanth S. Ramachandran, Michael R. Wilson, Gaud Catho, Geraldine Blanchard-Rohner, Nicoline Schiess, Randall J. Cohrs, David Boutolleau, Sonia Burrel, Tetsushi Yoshikawa, Anne Wapniarski, Ethan H. Heusel, John E. Carpenter, Wallen Jackson, Bradley A. Ford, Charles Grose

**Affiliations:** 1 Department of Neurology, Weill Institute for Neurosciences, University of California San Francisco, San Francisco, CA 94110, USA; Prashanth.Ramachandran@ucsf.edu (P.S.R.); Michael.Wilson@ucsf.edu (M.R.W.); awapniarski@gmail.com (A.W.); 2 Division of Pediatric Infectious Diseases, Geneva University Hospitals, Faculty of Medicine, University of Geneva, 1205 Geneva, Switzerland; Gaud.Catho@hcuge.ch; 3 Pediatric Immunology and Vaccinology Unit, Division of General Pediatrics, Department of Pediatrics, Gynecology and Obstetrics, Geneva University Hospitals, University of Geneva, 1205 Geneva, Switzerland; Geraldine.BlanchardRohner@hcuge.ch; 4 Department of Neurology, Johns Hopkins School of Medicine, Baltimore, MD 21205, USA; nschies1@jhmi.edu; 5 Department of Neurology, University of Colorado School of Medicine, Aurora, CO 80045, USA; randall.cohrs@cuanschutz.edu; 6 Virology Department, National Reference Center for Herpesviruses, Pitie-Salpetriere Hospital, Sorbonne University, 75013 Paris, France; david.boutolleau@aphp.fr (D.B.); sonia.burrel@aphp.fr (S.B.); 7 Department of Pediatrics, Fujita Health University School of Medicine, Aichi, Toyoake 470-1192, Japan; tetsushi@fujita-hu.ac.jp; 8 Division of Infectious Diseases/Virology, Department of Pediatrics, University of Iowa, Iowa City, IA 52242, USA; ethan-heusel@uiowa.edu (E.H.H.); john-carpenter@uiowa.edu (J.E.C.); wallen-jackson@uiowa.edu (W.J.); 9 Department of Pathology, University of Iowa, Iowa City, IA 52242, USA; bradley-ford@uiowa.edu

**Keywords:** varicella-zoster virus, Oka strain, human herpesvirus 6, human herpesvirus 7, corticosteroids, serious adverse event, herpes zoster, IL-6, IL-10, innate immunity

## Abstract

Varicella vaccine meningitis is an uncommon delayed adverse event of vaccination. Varicella vaccine meningitis has been diagnosed in 12 children, of whom 3 were immunocompromised. We now report two additional cases of vaccine meningitis in twice-immunized immunocompetent children and we perform further testing on a prior third case. We used three methods to diagnose or investigate cases of varicella vaccine meningitis, none of which have been used previously on this disease. These include metagenomic next-generation sequencing and cytokine multiplex profiling of cerebrospinal fluid and immunology exome analysis of white blood cells. In one new case, the diagnosis was confirmed by metagenomic next-generation sequencing of cerebrospinal fluid. Both varicella vaccine virus and human herpesvirus 7 DNA were detected. We performed cytokine multiplex profiling on the cerebrospinal fluid of two cases and found ten elevated biomarkers: interferon gamma, interleukins IL-1RA, IL-6, IL-8, IL-10, IL-17F, chemokines CXCL-9, CXCL-10, CCL-2, and G-CSF. In a second new case, we performed immunology exome sequencing on a panel of 356 genes, but no errors were found. After a review of all 14 cases, we concluded that (i) there is no common explanation for this adverse event, but (ii) ingestion of an oral corticosteroid burst 3–4 weeks before onset of vaccine meningitis may be a risk factor in some cases.

## 1. Introduction

Twenty years ago, virologists at the Food and Drug Administration (FDA) carried out an analysis of antibody titers of 4631 children collected over the 4 years after varicella vaccination. They interpreted the results as showing frequent asymptomatic reactivation of latent varicella vaccine virus in immunized children with low serum antibody titers after vaccination [1]. Subsequent epidemiologic studies have clearly shown that symptomatic reactivation as herpes zoster can occur after varicella vaccination, but the prevalence is less than that after wild-type varicella-zoster virus (VZV) infection [2]. Nevertheless, eight cases of varicella vaccine meningitis, a consequence of herpes zoster, have been reported in children who have received one varicella vaccination [3,4]. Even more recently, four cases of varicella vaccine meningitis in twice-immunized children have been reported [5]. We now report two new cases of varicella vaccine meningitis that occurred in twice-immunized, previously healthy boys. The first case is notable because identification of the virus as varicella vaccine virus was made by metagenomic next-generation sequencing (mNGS) of cerebrospinal fluid (CSF). Thus, this report provides proof-of-principle that mNGS can be used to differentiate vaccine-type from wild-type VZV strains.

The second case is notable because an immunology exome analysis was performed in an attempt to define risk factors for varicella vaccine meningitis in apparently healthy children. Additionally, we propose that we have found a previously unrecognized risk factor—namely, bursts of oral corticosteroids. Re-assessment of all 14 cases of varicella vaccine meningitis revealed that 1 of the new cases as well as a previously published varicella vaccine meningitis case had received a burst of oral corticosteroids 3–4 weeks before onset of varicella vaccine meningitis [6]. As a final goal of defining the CNS immunology environment during the meningitis event, we carried out cytokine multiplex profiling on CSF samples from one of the current cases and a prior case of varicella vaccine meningitis.

## 2. Materials and Methods

### 2.1. Patients and Ethics Statement

Two new patients are described. Furthermore, we performed additional testing on an archived sample from an earlier varicella meningitis patient and we reviewed the histories of all published cases to define risk factors. In order to distinguish each patient separately, we have chosen to continue the nomenclature that was used in an earlier review that summarized data from 12 patients with varicella vaccine meningitis [5]. Therefore, the two new cases will be called case 13 and case 14. The family of case 13 lived in the United States; the family of case 14 lived in both the United States and Switzerland. Both children received their varicella vaccinations in the United States. These studies were approved by the Institutional Review Boards at the respective medical centers.

### 2.2. Next Generation Sequencing of Cerebrospinal Fluid

VZV genotyping and a search for co-infection was performed on a CSF sample from case 13, using mNGS [7,8]. Total nucleic acid was extracted from 90 μL of CSF using the Zymo Quick-DNA/RNA MagBead (Zymo Cat. No. R2130) via the Agilent Bravo liquid handling robot (Santa Clara, CA, USA). The nucleic acid was then divided, with half undergoing DNAse treatment to isolate RNA for RNA sequencing (RNA-seq) and the remainder being used for DNA sequencing (DNA-Seq).

**Figure 1:**
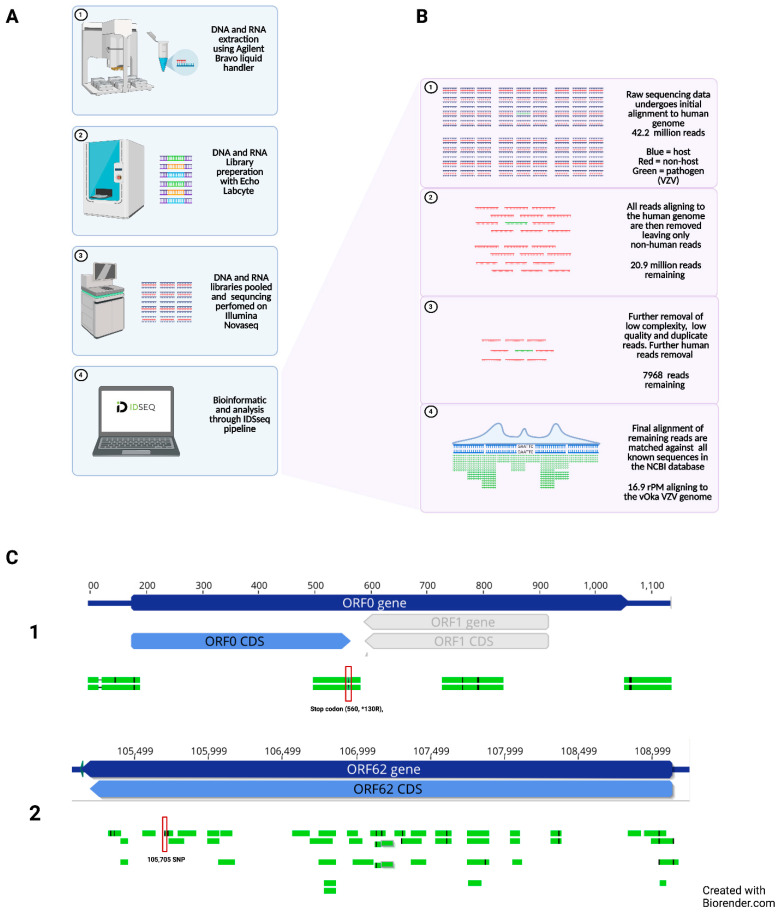
Identification of varicella vaccine virus DNA in CSF by mNGS. (**A**) Overview. Samples were processed on liquid handling robots; initially nucleic acid was extracted, and DNA and RNA underwent library preparation before being pooled and sequenced on the Illumina Novaseq at a sequencing depth of 42.2 million reads. Sequencing data were then analyzed using the open source IDseq pipeline. (**B**) Individual steps. The pipeline matched all data against the human genome, with removal of human reads (blue). Remaining non-host reads (red) underwent quality processing with the removal of redundant, low quality, and low complexity reads. The remaining reads were matched against the NCBI database for potential pathogens, with 16.9 rPM matching to the VZV genome (green). (**C**) VZV genes. Further analysis found 8 reads aligning with the ORF0 reference gene (panel **C1**) and 44 reads with the ORF62 reference gene (panel **C2**). Black lines within the reads (green) represent nucleotide mismatches or single nucleotide polymorphisms against the reference genome (blue). DNA, deoxyribonucleic acid; RNA, ribonucleic acid; VZV, varicella zoster virus; vOka, vaccine strain; ORF, open reading frame; CDS, CoDing Sequence.

RNA-Seq libraries were prepared using the New England Biolabs’ NEBNext Ultra II RNA library preparation kit (NEB Cat No. E7770, Ipswich, MA, USA), DNA libraries were prepared using the New England Biolabs’ NEBNext^®^ Ultra™ II DNA Library preparation kit (NEB Cat No. E7645), both using the Echo Labcyte 525 and Agilent Bravo robots with a previously described protocol. Host ribosomal RNA depletion was performed using the Qiagen QIAseq FastSelect RNA removal kit (Qiagen Cat No. 333180, Hilden, Germany) at 1:100 dilution. Pooled libraries were size selected using Ampure beads. Final libraries were then sequenced on an Illumina Novaseq 6000 (San Diego, CA, USA) using 146 base pair paired-end sequencing. The mNGS workflow events are also illustrated in Figure 1.

### 2.3. Sanger Sequencing

VZV genotyping on a CSF sample from case 14 was carried out as follows. After DNA extraction on the automated platform EMAG (BioMerieux, Marcy-l’Étoile, France), a duplex real-time PCR targeting the SNP108111 in ORF62 was performed on the LightCycler 480 (Roche Diagnostics, Basel, Switzerland) to discriminate vaccine from wild-type VZV. Thereafter, VZV genotyping was performed using conventional PCR and Sanger sequencing, targeting vaccine-associated SNPs in ORF1 (560, 685, and 703), ORF38 (69349), and ORF62 (105705, 106262, and 108111), as recommended by the Centers for Disease Control [9,10].

### 2.4. Immunology Exome Analysis

Immunology exome sequencing was performed on a panel of genes involved in immunologic and autoinflammatory disorders, by a method that has been previously described [11]. Exome capture was initiated by collecting genomic DNA extracted from white blood cells from case 14, using the SureSelect Human ALL Exon kit (Agilent Technologies, Santa Clara, CA, USA). High-throughput sequencing was performed in a HiSeq2000. The number of targeted genes was increased from 92 to 356. The full name of each gene as well as its genetic phenotype are listed in the catalog of Online Mendelian Inheritance in Man (OMIM) [12] or in HUGO (Human Genome Organization dictionary).

### 2.5. Measurement of Viral Antibody

VZV antibody in serum samples from case 13 was titrated by immunofluorescence (FAMA; fluorescent antibody to membrane antigen) on VZV-infected live cells [13]. Antibody titers to human herpes viruses (HHV) 6 and 7 were measured by immunofluorescence of fixed HHV6- and HHV7-infected cells [14]. Cell lines used in this report are available from the European Collection of Authenticated Cell Cultures and include MeWo cells (no. 93082609) and Molt-3 cells (90021901).

### 2.6. Measurement of Chemokines and Interleukins

CSF samples were stored frozen after the initial series of routine laboratory tests were performed. Cytokines in the CSF from cases 12 and 13 were analyzed by a bead-based multiplex fluorescence assay [15]. The Procartaplex Human Cytokine Magnetic 35-plex Bead Panel (Product #LHC6005M, Invitrogen/Thermo Fisher Scientific, Waltham, MA, USA) simultaneously evaluates 35 analytes (EGF, Eotaxin, FGF-basic, G-CSF, GM-CSF, HGF, IFNa, IFNγ, IL-1a, IL-1β, IL-1RA, IL-2, IL-2R, IL-3, IL-4, IL-5, IL-6, IL-7, IL-8, IL-9, IL-10, IL-12, IL-13, IL-17A, IL-17F, IL-22, IP-10, MIG, MIP-1α, MIP-1β, MCP-1, RANTES, TNFα, and VEGF). This assay was run on the Luminex^®^ FLEXMAP 3D^®^ instrument operated with xPONENT Software V4.2 (both from Luminex Corp., Austin, TX, USA). The cytokine profiling was carried out at the Bursky Center for Human Immunology and Immunotherapy Programs at the Washington University Immunomonitoring Laboratory, St. Louis, MO, USA.

## 3. Results

### 3.1. Varicella Vaccine Meningitis Case 13

Three days before admission, this previously healthy 7-year-old boy developed a headache, followed a few hours later by low grade fever. Two days before admission, his headaches worsened, and he began to vomit. Vomiting continued over the next 24 h whenever his parents attempted to feed him. When the vomiting persisted into the second day, he was admitted to a local hospital for intravenous (IV) hydration. His past history included febrile seizures at age 13 months and age 26 months; these were not further evaluated. He had received all recommended childhood immunizations, including Varivax at age 14 months and ProQuad at age 49 months.

After spending one night at the local hospital, he was transferred to a tertiary medical center. On admission, the child was in obvious pain. His physical examination did not disclose any rashes or abnormalities in major organ systems. Although the neurology examination was difficult to perform, he had mild nuchal rigidity but no focal neurological abnormalities. His non-contrast head CT imaging was normal.

His initial laboratory testing included the following values: ESR = 14 mm; CRP ≤ 0.5 mg/L; white blood cell count = 4000/mm^3^ with 1790 neutrophils, 1130 lymphocytes, and 380 eosinophils. His hemoglobin was 12.2 gm/dL; AST/ALT = 20/30 units/L and his urinalysis was normal. His cerebrospinal fluid (CSF) was hazy with a glucose of 50 mg/dL, total protein of 60 mg/dL, and nucleated cell count = 544 cells/mm^3^ with mainly lymphocytes. (A table of normal values is included in Table S1.) A Biofire FilmArray multiplex meningitis/encephalitis PCR (bioMerieux) was positive for both VZV and human herpesvirus 6 (HHV6). All Biofire VZV and HHV 6 reactions were repeated for verification.

Because the preliminary diagnosis was VZV meningitis, treatment with acyclovir IV at 30 mg/kg/day was initiated. The IV acyclovir was stopped after 4 days, when the child was markedly improved and his headaches resolved. He was discharged on a regimen of valacyclovir (500 mg three times daily) for 10 more days. The child was seen several times during the 18 months post discharge. His recovery from meningitis was complete.

### 3.2. Metagenomic Next Generation Sequencing of Cerebrospinal Fluid

Because a diagnosis of dual CNS infection with VZV and HHV6 was unexpected, we had an initial concern about a false positive result. In addition, the Biofire Filmarray panel cannot distinguish wild-type VZV from vaccine-type VZV. For the above two reasons, we pursued mNGS (Figure 1). Depth of sequencing for DNA-seq was 42,353,554 paired-end reads and 17,809,888 paired-end reads for RNA-seq. DNA-seq demonstrated 16.9rPM (716 reads) aligning to the VZV genome. RNA-seq had 1.6rPM (26 reads) aligning to VZV. The combination of both DNA and RNA reads allowed for 26.9% coverage of the VZV genome and definitive identification of the vaccine strain (GenBank AB097932), based on established criteria (Figure 1). Of great significance, one read aligning to ORF0 included the stop codon (560, *130R), which is mutated in the VZV vaccine strain [16]. More than 200 wild-type and vaccine-type VZV strains have been completely sequenced and the only other non-vaccine virus that has a similar ORF0 mutation is the highly passaged (>90) laboratory strain VZV Ellen (GenBank JQ972913) [17]. In addition, reads aligning with ORF62 contained additional SNPs found in the vaccine strain at nucleotides 105331, 105356, 105705, 107136, and 107165, including an extremely important fixed allele 105,705 [18]. Altogether, therefore, the mNGS data documenting a vaccine-type virus fulfilled the diagnostic criteria outlined by the Centers for Disease Control [9,10]. In addition, see Figure S1 for information about locations of the SNPs.

**Figure 2:**
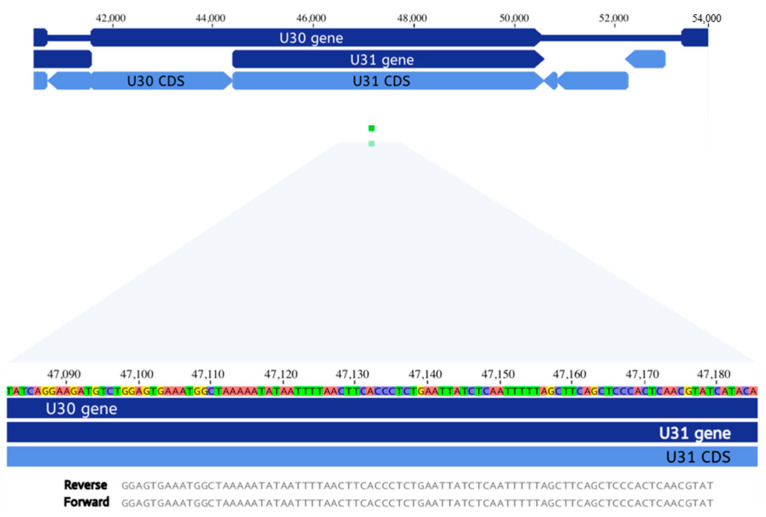
Identification of human herpesvirus 7 DNA in CSF by mNGS. The HHV7 nucleotide sequence includes 144,861 bp. The genome encodes ~100 open reading frames (ORFs). U30 includes 2816 bp; it is a capsid assembly protein. U31 includes 6179 bp, including the read from the mNGS of patient 13; it is a tegument protein. U = ORF located in the unique portion of the genome.

Further analysis of the mNGS data, however, failed to confirm the positive HHV6 result in the Biofire assay of the child’s CSF, as described above. Instead, DNA-seq revealed two reads of 85 bp length aligning to HHV7 (Figure 2). These two reads only matched to HHV7 and no other virus when processed through BLAST in the NCBI database (GenBank U43400.1).

### 3.3. Viral Antibody Studies

VZV-specific IgM and IgG antibody titers detected by immunofluorescence on patient 13 were positive at titers of 1:4 and 1:64, respectively. Because the Biofire meningitis panel had a positive HHV6 signal and the mNGS assay detected HHV7 sequences but no HHV6 sequences, we also performed testing for both HHV6 and HHV7 antibodies. The boy had detectable IgG antibodies to both HHV6 and HHV7 at a titer of 1:16.

### 3.4. Corticosteroid Burst Treatment Prior to Varicella Vaccine Meningitis

Patient 13 had reported an erythematous rash about 4 cm in diameter over the right knee about 20 days before admission to the local hospital for meningitis. When seen at a medical clinic, poison ivy was diagnosed and oral prednisolone was prescribed at a daily dosage of 20 mg for 7 days (Table 1). Because case 13 had received a corticosteroid burst, we re-assessed the available clinical data from the 12 previous cases of varicella vaccine meningitis. We discovered that case 12 had also received a short course of prednisone before onset of her neurological disease. The timing was similar to case 13, but the dosage (mg/kg/day) was much lower (Table 1). She had taken prednisone because of an exacerbation of asthma. Obviously, ingestion of oral corticosteroids may lead to a transient immunocompromised state in an otherwise healthy child with more severe VZV disease. For these reasons, we decided to investigate the cytokine inflammatory profiles in the CSF samples from both patients who had received the corticosteroid bursts.

### 3.5. Cytokine Profiling in the Cerebrospinal Fluid

An archived CSF sample from case 12 had been stored frozen [6]. CSF samples from cases 12 and 13 were examined in a 35-well cytokine multiplex assay (Table 2). The 10 most elevated cytokines in our patients’ CSF samples included interferon gamma, interleukins IL-1RA; IL-6, IL-8, IL-10, and IL-17F; chemokines CCL2, CXCL9, and CXCL10; as well as G-CSF. Of note, IL-8 is elevated in CSF of adult cases of severe herpes zoster [19]. Granulocyte colony-stimulating factor (G-CSF) was first shown to be elevated in acute viral meningitis many years ago [20]. A table of normal values is included in Table S1.

### 3.6. Case 14 and Immunology Exome Sequencing

A previously healthy 12-year-old boy presented to the emergency department with a 1-day history of fever, headache, phonophobia and photophobia. His immunizations were up to date, including varicella vaccinations (Varivax) at 14 months and 30 months. Because the child had neck stiffness, a lumbar puncture was performed. Laboratory analysis of CSF revealed 83 white cells/mm3 (88% lymphocytes). The CSF was tested by the meningitis panel and found to be positive for VZV and negative for enterovirus, herpes simplex virus, and HHV6, as well as bacteria. The child was hospitalized, and acyclovir IV (30 mg/kg/day) was initiated. No radiologic studies were ordered. On day 5, he was discharged home; his acyclovir was switched to oral administration for a total treatment course of 14 days. The virus in the CSF was confirmed to be the VZV vaccine strain by Sanger sequencing, as described in methods by the Centers for Disease Control [9,10]. Specifically, the fixed alleles found in the vaccine strain are included in Table 1 [10]. Because we were aware of results from case 13, we also performed a PCR analysis for HHV-7 DNA on the CSF; that test was negative.

Patient 14 also had an extensive immunology evaluation, which showed normal serum immunoglobulin IgG, IgA, and IgM levels and normal serum levels of vaccine antibodies for all tested antigens (measles, varicella, tetanus, and diphtheria). Anti-VZV antibodies were detected using a commercial VZV ELISA kit. An extended immunophenotyping showed normal subpopulations of B, T, and NK cells. Finally, genetic analysis was performed by immunology exome sequencing, which included a panel of 356 genes of primary immunodeficiency (Table 3). However, no errors in innate immune genes or other rare primary immunodeficiencies were discovered; specifically, there were no errors in RNA polymerase III genes (POLR3A and POLR3C) [21]. RNA polymerase III is a sensor of foreign DNA in the cytosol; errors in RNA polymerase III genes have been found in some people with severe wild-type varicella meningoencephalitis [21]. Furthermore, a frequently cited gene involved in susceptibility to HSV1 meningoencephalitis (TLR3) was intact [22]. The child had undergone a complete recovery when seen at 1 and 12 months after his hospitalization.

## 4. Discussion

**Figure 3:**
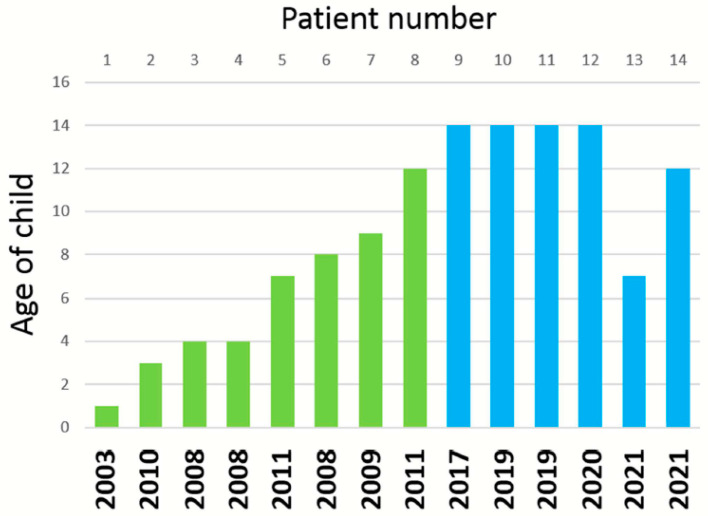
Fourteen cases of varicella vaccine meningitis. The cases are arranged first by whether the child had one varicella vaccination (green bar) or two varicella vaccinations (blue bar). Then, the height of each bar graph indicates the age of the patients when they developed meningitis. The year under each bar indicates the year in which the case was first reported in the medical literature. Data about the first 12 cases are taken from Reference 5.

Varicella vaccine meningitis presumably represents the clinical manifestations of herpes zoster of the trigeminal ganglion, whereby afferent fibers carry reactivated virus to the meninges [23,24]. An alternative source of virus is the superior cervical ganglion, which also houses latent virus and also innervates the dura [25]. This serious adverse event is rare. Only one case of varicella vaccine meningitis had been described before 2006 [26], when the CDC switched their recommendation from one to two varicella vaccine doses for children (Figure 3). With inclusion of the 2 new cases reported herein, the current tally is 14 cases (Figure 3). This tally includes eight children who only received one varicella vaccination (Table 4) [3,4]. There are now six case reports of varicella vaccine meningitis in twice-immunized immunocompetent adolescents [6,27,28]. There is also no pattern in the timing between meningitis and a second varicella vaccination (Table 4). We have recently reviewed the epidemiology of wild-type varicella meningitis in immunocompetent children [5]. Among the published cases, about one-half are preceded by clinical herpes zoster and one-half are not preceded by herpes zoster. Of interest, the majority of cases of wild-type varicella meningitis have occurred in adolescents, a possible suggestion of waning immunity since a bout of chickenpox in early childhood. The adolescents have recovered with no permanent neurological sequelae.

### 4.1. Corticosteroids and VZV Reactivation

In 2021, a group from Taiwan surveyed the medical records of 4.5 million children in their national database and reported an association between oral corticosteroid bursts and increased severe adverse events in children, including gastrointestinal bleeding, sepsis, pneumonia, and glaucoma [29]. Case 13 was diagnosed with poison ivy on the right knee 3 weeks before his bout of meningitis. After a visit to a local clinic, he was treated with prednisolone 20 mg daily for 7 days (Table 1). Based on this observation, we reviewed all prior case reports of varicella vaccine meningitis in immunocompetent children who had received either one or two vaccinations. Reports of meningitis in the once-immunized children contained no information about corticosteroid prescriptions. Among the three prior cases of varicella vaccine meningitis in immunocompetent children who had received two varicella vaccinations, the report of case 12 mentioned a prior corticosteroid burst.

There has been extensive reporting on adverse consequences in people who received corticosteroids prior to or during VZV infection, dating back almost 70 years, long before acyclovir was available [30,31]. Corticosteroids increased the likelihood of disseminated varicella and subsequent death; corticosteroids also facilitated VZV reactivation to cause herpes zoster [32]. Prednisone dosages usually associated with these severe consequences ranged from 0.5 to 2 mg/kg/day. Based on these publications, treatment with a prednisolone burst at a relatively high dosage likely allowed latent vaccine virus to reactivate in case 13. Further, we again point out the serological data that showed frequent subclinical reactivations in healthy children who had received varicella vaccination [1]. If any vaccinated child received a steroid burst during a subclinical reactivation, perhaps that child might develop clinical herpes zoster and even varicella vaccine meningitis.

### 4.2. Specificity of mNGS and the Biofire Filmarray Encephalitis Panel

In an analysis of specificity of the Biofire meningitis/encephalitis panel by the Biofire Company, they reported 1/7 false-positive results for VZV [33]. With regard to VZV specificity, we point out the overall importance of mNGS methodology for finalizing the primary diagnosis from an extremely small CSF sample (90 μL). Indeed, mNGS has the capability to assess genomic data in a sample that falls outside targeted PCR primer regions, allowing for better strain identification. Furthermore, any SNPs that may be present at prime sites leading to PCR failure could be captured by mNGS.

The data about HHV6 and HHV7 are a true conundrum. When patient 13 was hospitalized, we initially considered a positive HHV6 result in CSF by Biofire assay to be a false positive [34,35]. When reviewing the mNGS data, however, a new hypothesis emerged. We observed two 85 base-pair reads specific for HHV7, not HHV6 (Figure 2). When considered along with that result, we now think that there are two possible explanations for the positive PCR for HHV6 on the Biofire panel: (i) The Biofire result was a true positive, but the amount of HHV6 DNA detectable by the Biofire assay was below the limits of detection by mNGS and (ii) the Biofire result for HHV6 was a false positive in that primers for HHV6 in the Biofire assay amplified conserved sequences in the closely related HHV7 genome. In an analysis of specificity in the original publications about the Biofire assay by the Biofire company, they observed that HHV6 had the highest number of false positives, but they did not mention the possibility of HHV7 amplification by the HHV6 primers in the Biofire kit. The HHV7 U31 gene was discovered in the mNGS analysis, but that gene is unlikely to be a target in the Biofire kit because there is only a 46% identity to its HHV6 homolog [36]. However, the Biofire kit could be recognizing a HHV7 gene such as U77, which has 75% identity to its HHV6 homolog [36].

We also note that HHV7 was codetected in the CSF of patient 13 but not patient 14. The severity of meningitis was much greater in patient 13 than patient 14, as documented by the decision not to perform any CNS imaging on patient 14. A strikingly similar result was found earlier in a large virological analysis of childhood meningoencephalitis—namely, HHV7 was codetected in 2 of 5 cases of Epstein–Barr virus meningoencephalitis [37]. This pattern fits into a hypothesis that HHV7 codetection may be a marker for increased severity of viral meningoencephalitis.

### 4.3. Cytokine Profiles in CSF after Neurotropic Virus Infection

**Figure 4:**
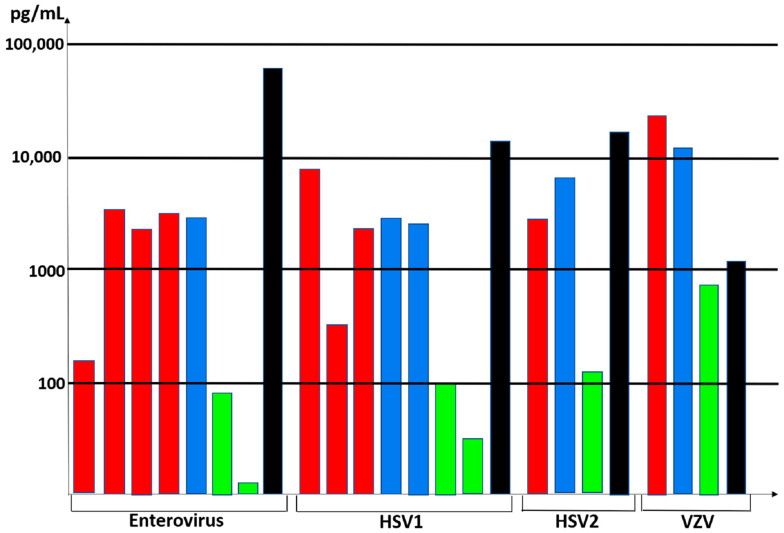
Cytokine levels in CSF from patients with HSV1, HSV2, enterovirus, and VZV infections in the central nervous system. Cytokine data about HSV1, HSV2, and enterovirus were obtained from References 38–46; data about VZV are from the current report. Bars: red = IL-6; blue = IL-8; green = IL-10; black = CXCL10.

The cytokine profiles from cases 12 and 13 were very revealing when compared with the cytokine profiles in CSF samples from patients with enterovirus, HSV1, HSV2, and VZV CNS infections (Figure 4). The concentrations in the figure represent the highest values for each cytokine within each study group [38,39,40,41,42,43,44,45,46]. We selected IL-6, IL-8, IL-10, and CXCL-10 (IP-10) for inclusion in the figure because there were several papers that included these cytokines. As is apparent from reviewing the table, there is a commonality to the cytokine profiles in the CSF during both enterovirus and alpha herpesvirus CNS infections. Of note for VZV, extremely high IL-6 levels are also seen in a human skin organ culture model for VZV infection [47]. Because lumbar punctures are not performed in healthy children, there are minimal data on normal cytokine levels in the cerebrospinal fluids.

### 4.4. Elevated CXCL10 in CSF

The importance of elevated CXCL10 in the CSF deserves further discussion. Increased CXCL10 production in the dorsal root ganglion (DRG) has been shown to be a manifestation of an antiviral host response to peripheral neuronal infection by wild-type VZV [48]. Increased CXCL10 has also been found in the CSF of patients with wild-type VZV infection [49]. Furthermore, increased CXC10 production has been documented in the DRG following reactivation of the closely related simian varicella virus [50]. Thus, the inflammatory response to varicella vaccine virus infection in the CNS closely mimics the response to wild-type virus infection in both the CNS and the peripheral nervous system [49].

## 5. Conclusions

To provide perspective, universal varicella vaccination has been the national public health policy in the United States for decades [51,52]. Over 200 million doses have administered, and the safety profile of the live attenuated varicella vaccine is excellent [53,54]. Although impaired for reactivation from latency, the vaccine virus certainly does enter ganglia and reactivate [55]. An original hypothesis put forward by Hope-Simpson was the need for periodic subclinical VZV reactivations to maintain VZV immunity and delay clinical herpes zoster [56]. An overview of VZV evolution also suggests that VZV reactivation is a mechanism by which the virus achieves fitness throughout the lifetime of its human host [57]. Therefore, it seems likely that periodic reactivations, usually subclinical but occasionally clinical, are required after live varicella vaccination to maintain immunity within immunized children into early adulthood [1,2]. We encourage continued varicella vaccination of young children [58].

## Figures and Tables

**Table 1:** History of corticosteroid burst before varicella meningitis.

Category	Patient 13	Patient 12
Gender	Male	Female
1st Vaccine	1 Year	1 Year
2nd Vaccine	4 Years	5 Years
Meningitis	7 Years	14 Years
Head Image	Normal	Normal
Prior Zoster	No	Yes
VZV type	Vaccine	Vaccine
Corticosteroids	Prednisolone	Prednisone
Time Before Meningitis	3 Weeks	4 Weeks
Daily Dosage	20 mg	20 mg
Mg/Kg/Day	0.8 mg/kg	0.3 mg/kg
Duration	7 Days	5 days
Reason for Steroids	Poison Ivy	Asthma
Follow-up	Recovered	Recovered

**Table 2:** Cytokine levels in the cerebrospinal fluid.

Cytokine	PT. 13	PT. 12	Cytokine	PT. 13	PT. 12
IL-1A	3.19	1.02	IFNa	104.44	38.79
IL-1RA	184.63	1620.65	IFNg	75.76	865.85
IL-1b	6.14	3.70	MIP1a	39.27	117.88
IL-2	8.74	12.63	CXCL10	1178.58	507.83
IL-2R	104.40	79.92	CCL11	3.91	2.86
IL-3	10.42	11.61	Rantes	115.95	30.76
IL-4	16.42	23.54	GM-CSF	1.74	3.21
IL-5	43.76	11.55	TNFa	75.76	1.07
IL-6	90.57	31,816.58	HGF	93.61	187.21
IL-7	10.22	11.99	MIP-1b	91.13	156.86
IL-8	4034.30	11,604.79	CCL2	616.79	3025.46
IL-9	8.83	6.50	FGF2	28.21	15.03
IL-10	75.39	846.40	VEGF	2.35	4.36
IL-12	72.32	39.31	G-CSF	55.79	171.57
IL-13	15.48	18.67	CXCL9	81.16	273.94
IL-15	56.41	26.00	EGF	48.25	42.61
IL-17A	7.22	3.67			
IL-17F	348.68	72.26			
IL-22	22.12	70.20			

Note: The full name for each abbreviation is given in the catalog of Online Mendelian Inheritance in Man (OMIM^R^) or HUGO (Human Genome Organization dictionary).

**Table 3:** Immunology exome sequencing of a panel of 356 genes.

ACD	C8B	CFP	FADD	IL10RA	LPIN2	NSMCE3	RSGRP1	SPINK5	TRAC
ACP5	C9	CFTR	FAS	IL10RB	LRBA	OAS1	RBCK1	SPPL2A	TRAF3IP2
ADA	CARD11	CHD7	FASLG	IL12B	LYST	ORAI1	RC3H1	SRP54	TREX1
ADA2	CARD14	CIB1	FAT4	IL12RB1	MAGT1	OTULIN	RECQL4	STAT1	TRIM22
ADAM17	CARD9	CIITA	FCGR3A	IL17F	MALT1	PARN	RELA	STAT2	TRNT1
ADAR	CARMIL2	CLPB	FCHO1	IL17RA	MAP3K14	PAX1	RFX5	STAT3	TTC37
AICDA	CASP10	COPA	FERMT3	IL17RC	MBL2	PEPD	RFXANK	STAT5B	TTC7A
AIRE	CASP8	CORO1A	FNIP1	IL1RN	MCM4	PGM3	RFXAP	STIM1	TYK2
AK2	CCBE1	CSF2RA	FOXN1	IL21	MEFV	PIK3CD	RHOH	STING1	UNC13D
ALPI	CD19	CSF2RB	FOXP3	IL21R	MOGS	PIK3CG	RIPK1	STK4	UNC93B1
AP1S3	CD247	CSF3R	FPR1	IL2RA	MPO	PIK3R1	RNASEH2A	STX11	UNG
AP3B1	CD27	CTC1	G6PC3	IL2RB	MRTFA	PLCG2	RNASEH2B	STXBP2	USB1
AP3D1	CD3D	CTLA4	G6PD	IL2RG	MSN	PMS2	RNASEH2C	TAP1	USP18
ARPC1B	CD3E	CTPS1	GATA1	IL36RN	MTHFD1	PNP	RNF168	TAP2	VPS13B
ATM	CD3G	CTSC	GATA2	IL6R	MVK	POLA1	RNF31	TAPBP	VPS45
ATP6AP1	CD4	CXCR4	GFI1	IL6ST	MYD88	POLD1	RORC	TAZ	WAS
B2M	CD40	CYBA	GINS1	IL7R	MYO5B	POLE	RPSA	TBK1	WDR1
BACH2	CD40LG	CYBB	GUCY2C	INO80	MYSM1	POLR3A	RTEL1	TBX1	WIPF1
BCL10	CD46	CYBC1	HAVCR2	IRAK4	NBN	POLR3C	SAMD9	TCF3	XIAP
BCL11B	CD55	DBR1	HAX1	IRF3	MCF1	POMP	SAMD9L	TCN2	ZAP70
BLM	CD59	DCLRE1B	HELLS	IRF7	NCF2	PRF1	SAMHD1	TERT	ZBTB24
BLNK	CD70	DCLRE1C	HPS1	IRF8	NCF4	PRKCD	SBDS	TET2	ZNF341
BLOC1S6	CD79A	DEF6	HPS4	IRF9	NCKAP1L	PRKDC	SEC61A1	TFRC	CR2
BTK	CD79B	DKC1	HPS6	ISG15	NCSTN	PSENEN	SERPING1	TGFB1	
C1QA	CD81	DNAJC21	HTRA2	ITCH	NFE2L2	PSMA3	SGPL1	TICAM1	
C1QB	CD8A	DNASE1L3	ICOS	ITGB2	NFKB1	PSMB10	SH2D1A	TINF2	
C1QC	CDC42	DNASE2	IFIH1	ITK	NFKB2	PSMB4	SKIV2L	TLR3	
C1R	CDCA7	DNMT3B	IFNAR1	ITPKB	NFKBIA	PSMB8	SLC29A3	TLR7	
C1S	CEBPE	DOCK2	IFNGR1	IVNS1ABP	NHEJ1	PSMB9	SLC35C1	TMC6	
C2	CFB	DOCK8	IFNGR2	JAGN1	NHP2	PSTPIP1	SLC37A4	TMC8	
C3	CFD	EFL1	IGHM	JAK3	NLRC4	PTEN	SLC39A7	TNFAIP3	
C4A	CFH	ELANE	IGKC	KRAS	NLRP1	PTPN2	SLC46A1	TNFRSF11A	
C4B	CFHR1	EPG5	IGLL1	LAMTOR2	NLRP12	PTPRC	SLC7A7	TNFRSF13C	
C5	CFHR3	ERBIN	IKBKB	LAT	NLRP3	RAB27A	SMARCAL1	TNFRSF1A	
C6	CFHR4	ERCC6L2	IKBKG	LCK	NOD2	RAC2	SMARCD2	TNFRSF9	
C7	CFHR5	EXTL3	IKZF1	LIG1	NOP10	RAG1	SOCS1	TOP2B	
C8A	CFI	F12	IL10	LIG4	NRAS	RAG2	SP110	TPP2	

Note: The full name for each abbreviation is given in the catalog of Online Mendelian Inheritance in Man (OMIM^R^).

**Table 4:** Interval between varicella vaccination and varicella meningitis.

Case	Age	Vaccine	Interval-1	Interval-2
**1**	1.2 year	1	11 weeks	–
**2**	3.5 years	1	20 months	–
**3**	4 years	1	18 months	–
**4**	4 years	1	32 months	–
**5**	7 years	1	6 years	–
**6**	8 years	1	7 years	–
**7**	9 years	1	8 years	–
**8**	12 years	1	11 years	–
**9**	14 years	2	13 years	2 years
**10**	14 years	2	13 years	10 years
**11**	14 years	2	13 years	4 years
**12**	14 years	2	13 years	9 years
**13**	7 years	2	6 years	2 years
**14**	12 years	2	11 years	9 years

Note: Interval-1 is the time between meningitis and the first varicella vaccination; interval-2 is the time between meningitis and the second varicella vaccination, among the group who had received two vaccinations. Age indicates the age when meningitis was diagnosed. Dash (–) means none.

## Supplementary Materials

The following are available online at https://www.mdpi.com/article/10.3390/v13112286/s1, Figure S1: Additional data about mNGS reads, Table S1: Normal values for blood and cerebrospinal fluid tests.
