# Supplementary material for: Meningitis Caused by the Live Varicella Vaccine Virus: Metagenomic Next Generation Sequencing, Immunology Exome Sequencing and Cytokine Multiplex Profiling
Source: Viruses. 2021 Nov 16;13(11):2286. doi: 10.3390/v13112286 (PMC8620440; doi:10.3390/v13112286)
Supplement: Supplementary file 1 [file viruses-13-02286-s001.zip › viruses-1434602-supplementary.pdf]

Supplemental Figure S1. Additional data about mNGS reads.

CTCCACGTAGCAAAGCTACACTTTTTGCATCAGCCTCCACTTCGTCTGTGGGGGCC
GGCCCCCACAGACGAAGTGGAGGCTGATGCAAAAAGTGTAGCTTTGCTACGTGGAG
CATTAAATGTAATAACCACGCCGGCCAGCAATGTTTTAATTTTATATACAAAATAAAAACATACACCAGAAACGTTTTTAGTTTTTAT
T
AATAAAAACTAAAAACGTTTCTGGTGTATGTTTTTATTTTGTATATAAAATTAAAACATTGCTGGCCGGCGTGGTTATTACATTTAATG
CTAGACGCGGCCGATCCAGCCCGTGTTGTATCGTTCTCGCCCATTATACGATCAACCGCACGTGTGCTGTGAG
CTCACAGCACACGTGCGGTTGATCGTATAATGGGCGAGAACGATACAACACGGGCTGGATCGGCCGCGTCTAG
TTCCCCGAGACACAAAGATTATCGGGTGAACCGGAACCTACAACCACCTAGTATACCTTAACTCAACCGCCGTTGTGGAAAGGTATATGTC
AACATTTACAGTAATATATTAAA
TTTAATATATTACTGTAAATGTTGACATATACCTTTCCACAACGGCGGTTGAGTTAAGGTATACTAGGTGGTTGTAGGTTCCGGTTCACCCGA
TAATCTTTGTGTCTCGGGGAA
CACACGGATCCTGTGTCATTTCGGTAGAACCGGGAGGGCGTTTAAATTGATATTC
GAATATCAATTTAAACGCCCTCCCGGTTCTACCGAAATGACACAGGATCCGTGTG
GATCACGCAAGTAATCCCAACTAAACGCCCACGTGAAGTAACTGAAAACGATGAAGAAAACCAAATAATGCATTTATTTT
AAAATAAATGCATTATTTGGTTTTCTTCATCGTTTTCAGTTACTTCACGTGGGCGTTTAGTTGGGATTACTTGCGTGATC
GAAACAATTGCTGTTCTGTTGGCATCCGCTGCCTGTTTATCCGTATATTCTTCTGCCCGGCAT
ATGCCGGGCAGAAGAATATACGGATAAACAGGCAGCGGATGCCAACAGAACAGCAATTGTTTC
AACATACATTCTCCCGGCGGACTGTAGACATGTTACCGTTGTGTTCATAAACGTACTGGAAATGCGCCCGTCTTTACAATC
GATTGTAAAGACGGGCGCATTTCCCGTACGTTTATGAACACAACGGTAACATGTCTACAGTCCGCCGGGAGAATGTATGTT
GGGATAATGGCCGTTGGGCAGGCCTCCAAAAGGCAATTTGAGCGTTTTAAGTTTGGGGTACGTTTTTAAAGCTTGATTGGCAAACGTT
ACCGCAAATC
GATTTGCGGTAACGTTTGCCAATCAAGCTTTAAAAACGTACCCCAAACTTAAAACGCTCAAATTGCCTTTTGGAGGCCTGCCCAACG
GCCATTATCCC
CCCGATATTGTATTGTAGTACAGGGCAATGGGAATTGATGATCCCAATAAACGGCATAGACGCACAGCGCCGTTATA
TATAACGGCGCTGTGCGTCTATGCCGTTTATTGGGATCATCAATTCCCATTGCCCTGTACTACAATACAATATCGGG
CCGATTTACCGTGTTTACGGCAGTCTGACCATTTACCAGTGTATGTCTGTAATACGACGTTGTTG
CAACAACGTCGTATTACAGACATACACTGGTAAATGGTCAGACTGCCGTAAACACGGTAAATCGG
GGATTACGAACCATAATTATCTGTCTTGGTTTATAATCCTTATCTTGCATGTGTCCGTAT
ATACGGACACATGCAAGATAAGGATTATAAACCAAGACAGATAATTATGGTTCGTAATCC
ATACATAATACAAATATCACGGTGCTTGCGGTTTCCAGTATTTATTCGTATCGCTACAACGTTAAATG
CATTTAACGTTGTAGCGATACGAATAAATACTGGAAACCGCAAGCACCGTGATATTTGTATTATGTAT
CCCTGTAAACGCACCAGCGTCACAGCGTTCTAGATAAAAAAGCAGTCGCGCCA
TGGCGCGACTGCTTTTTTATCTAGAACGCTGTGACGCTGGTGCGTTTACAGGG
TAAACTTCAAGTGTCCAGATGAGACAGCTAGGGTGGAGAAGTCATCTCCAAGTTGTTTTTG
CAAAAACAACTTGGAGATGACTTCTCCACCCTAGCTGTCTCATCTGGACACTTGAAGTTTA
CTTCTGACGGCTATAGCAGTGAGAGTGAAAATTCTATCGTACTAAGTCTCGACGAACATCGGTCATG
CATGACCGATGTTCGTCGAGACTTAGTACGATAGAATTTTCACTCTCACTGCTATAGCCGTCAGAAG
CTAGCGTTTAGTGTAAAACGTGGCATAACACCACCGTCTAGTCTTAAAAATATTTTTGCGGCCCTCATG
CATGAGGGCCGCAAAAATATTTTTAAGACTAGACGGTGGTGTTATGCCACGTTTTACACTAAACGCTAG
TAAATTGTGTTGTTGTCTGTTCTATACGCGCACGCAGTGTGTCTAATATGCGACGAAACGTTGGAT
ATCCAACGTTTCGTCGCATATTAGACACACAGCGTGCGCGTATAGAACAGACAACAACACAATTTA
GGAGAAAAAGCCAAAGAGTCGTGAGTTTGTTTCGTCTTCATCCTCTTCCTCGTCGTGGG
CCCACGACGAGGAAGAGGATGAAGACGAAACAAACTCACGACTCTTTGGCTTTTTCTCC
CGCGCCGGCATGATATACCGCCCCCCCATGGCGTGTAGTCT
AGACTACACGCCATGGGGGGGCGGTATATCATGCCGGCGCG
TGTAACCAGTTTTTCGCAATATTGTTCATCTGTAAAATATGTATCACAGCTTCCGTATATAAGCG
CGCTTATATACGGAAGCTGTGATACATATTTTACAGATGAACAATATTGCGAAAAACTGGTTACA
GCACGTACAGAAATAATACGCAATGAGTCTATACACCCCGAGGTACAGATTGCC
GGCAATCTGTACCTCGGGGTGTATAGACTCATTGCGTATTATTTCTGTACGTGC
GGGCTGGATCGGCCGCGTCTAGACTTGTAAAGCTAATAGTTAACTTAAAAAACATGAGACATG
CATGTCTCATGTTTTTTAAGTTAACTATTAGCTTTACAAGTCTAGACGCGGCCGATCCAGCCC
ATTCAGAAACATTAGTTGCGGTTCAAGAACTACTGAACTCCGAGATGGATCAGGACAGCAGTTCTGACGCATCGGATGATTTTCCGG
G
CCCGGAAAATCATCCGATGCGTCAGAACTGCTGTCCTGATCCATCTCGGAGTTCAGTAGTTCTTGAACCGCAACTAATGTTTCTGAAT
CATGGAAACGGATGGGTTGTGGCCAGTAACCGGTATTCAGGCCTTTCATTATCTAGCC
GGCTAGATAATGAAAGGCCTGAATACCGGTTACTGGCCACAACCCATCCGTTTCCATG
GTTTTTTGGTGACGTGGCAGATGTGCTTGCATCCGACATCGCGACCGTATCTACTAACCCGGAAAGTGAGTCCGCCATAAACGCTGT
ACAGCGTTTATGGCGGACTCACTTTCAGGGTTAGTAGATACGGTCGCGATGTCGGATGCAAGCACATCTGCCACGTCACCAAAAAAC
TACAGCAAGCCTTGTCTATTCTTCAGTGGGCTCGCGATTATGGATCCGGAACCGTGCCCAATGCAG
CTGCATTGGGCACGGTTCCGGATCCATAATCGCGAGCCCACTGAAGAATAGACAAGGCTTGCTGTA
AAATAACGCCAACTCAACCGTTACGGCTGTAAGTTCTGCGTGTTTTTCATGTTCGTTTAATACATCCAA
TTGGATGTATTAAACGAACATGAAAAACACGCAGAACTTACAGCCGTAACGGTTGAGTTGGCGTTATTT
CGATTGGGAGGTGGGGTGTGAAGACGCGTGGGACAGCGAGGAGGGGGGCG
CGCCCCCCTCCTCGCTGTCCCACGCGTCTTCACACCCCACCTCCCAATCG
GTGAGTTTTAAGGTAATACCAGCTCCGGCAAGACCCGTAGTGCGTGCTCCAGAAACCACCGCCAGGCTGGATG
CATCCAGCCTGGCGGTGGTTTCTGGAGCACGCACTACGGGTCTTGCCGGAGCTGGTATTACCTTAAAACTCAC
CCCATTAGATAAAAGTCGAGGCATATGCGCCGCAATCTTCGTGAATCTCGCGTGACGCGTCCCGAC
GTCGGGACGCGTCACGCGAGATTCACGAAGATTGCGGCGCATATGCCTCGACTTTTATCTAATGGG
GCCGTATTTATGTGCAACGTGTTTCTGCATTATGGAGGACTCGATATTGTTCATATTA
TAATATGAACAATATCGAGTCCTCCATAATGCAGAAACACGTTGCACATAAATACGGC
GGAATGACAATATAGTAGTAAATCTTGGAGAGAATAACCCACACCATGACCGTGAATGGGG
CCCCATTCACGGTCATGGTGTGGGTTATTCTCTCCAAGATTTACTACTATATTGTCATTCC
CCCAGGACGCCGAAACAAAACCCACGTTTTACGTCTGCCCACCGCCAACAGGCTCCACAATCGTACGATTAGA
TCTAATCGTACGATTGTGGAGCCTGTTGGCGGTGGGCAGACGTAAAACGTGGGTTTTGTTTCGGCGTCCTGGG
CCTCCAGGCGTCATTGCACGCCCTATTAGACGGGCAAGGCTAAGACGTTCGCCGGGT
ACCCGGCGAACGTCTTAGCCTTGCCCGTCTAATAGGGCGTGCAATGACGCCTGGAGG
CTGTACAACACAAAGGGCCTCTAACAATGCACTGAACCACAACCAAGCTATGGACGCAACGCAGATTACCTTGGTTAGAG
CTCTAACCAAGGTAATCTGCGTTGCGTCCATAGCTTGGTTGTGGTTCAGTGCATTGTTAGAGGCCCTTTGTGTTGTACAG
TAAACGCTTTAATATGGAATTTCCATATCATTCAACCGTATCTTATAACGGCGTAACGTTTTATTTTAACGAGCGTGCTACCAG
CTGGTAGCACGCTCGTTAAAATAAAACGTTACGCCGTTATAAGATACGGTTGAATGATATGGAAATTCCATATTAAAGCGTTTA
CCCCCCGGGGTCCGCCGGGCGCCCAGAAACCGGGGGGGGTTATTTTCGGGGGGGGTCCGACCAGCCCCCCCGTCGCCCGCCCCCAC
C
TGTGCGGGCGGGCGACGGGCGGGCTGGTCGGACCCCCCCCGAAAATAACCCCCCCCGGTTTCTGGGCGCCCGGCGGACCCCGGGGG
G
GTAACGCACAGAAGTTTCTTTCGTTTCCATGAATAACAGCCTCTAGCATGTAGAAAATAG
CTATTTTCTACATGCTAGAGGCTGTTATTCATGGAAACGAAAGAAACTTCTGTGCGTTAC
ACCACTTTGGCGATGATTTCTCGCATTAAGGTCTGGGGGATGGTTAAAGGGAAAACAATTTCCAGTCCTACAGAG
CTCTGTAGGACTGGAAATTGTTTTCCCTTTAACCATCCCCCAGACCTTAATGCGAGAAATCATCGCCAAAGTGGT
ATAGAACACCAAGAAGTTGATCGGCAGTGCCTCGTTCAAATGAGTCCAAAACGGTGCGTAAATTACGAGCCATCTGTTGG
CCAACAGATGGCTCGTAATTTACGCACCGTTTTGGACTCATTTGAACGAGGCACTGCCGATCAACTTCTTGGTGTTCTAT

CAGTCGGTTCTACGGTGCCGTAAGAAGAGTACCCGAGCGGGTAC
GTACCCGCTCGGGTACTCTTCTTACGGCACCGTAGAACCGACTG
CATCCAATTGCAGTTCCAGGCTAATTCCCGGGTGGGTGTGTTTAATCCACGAAACCAGATCCCCTCCATATAACGCCCGC
GCGGGCGTTATATGGAGGGGATCTGGTTTCGTGGATTAAACACACCCACCCGGGAATTAGCCTGGAACTGCAATTGGATG
TACGTTTTCATTTACCGACGGGTCTTACAGCACAGCGGTGTTCGCGTCA
TGACGCGAACACCGCTGTGCTGTAAGACCCGTCGGTAAATGAAAACGTA
GAAGCCGTACGGCTTGCACATCAGGTTTTTGAAACCTGGCGTCAAACATTACAGTCATCGTTACTTGAG
CTCAAGTAACGATGACTGTAATGTTTGACGCCAGGTTTCAAAAACCTGATGTGCAAGCCGTACGGCTTC
GCGAATTCCTCCGGTTGCAGCGTATTCCACATTGACACTAAGGAAATTGGCGGGAGACACATGCGCCCAAG
CTTGGGCGCATGTGTCTCCCGCCAATTTCCTTAGTGTCAATGTGGAATACGCTGCAACCGGAGGAATTCGC
CAAGTGTCTCAGAAATTACATGCATTTTATGATTTTACGGGGGTGGGTTACGATAATAGCTATTAAACAAACACCCAATAAAGGTTC
TACAAAAAAATGCTTTATTGGG
CCCAATAAAGCATTTTTTGTAGAACCTTTATTGGGTGTTTGTTTAATAGCTATTATCGTAACCCACCCCCGTAAAATCATAAAATGCAT
GTAATTTCTGAGACACTTG
ATAACATCTTTGTAATATACCGTCGCCTTAAACTTGTACGCGGCAATGTTTTCTTTATAAACA
TGTTTATAAAGAAAACATTGCCGCGTACAAGTTTAAGGCGACGGTATATTACAAAGATGTTAT
TAACTACACGCAAACATTCAAAACACCGTGGTATCATTAACGTCATATGAAAAGATCCAATCAATCCAATCAACCACACCTCCTACC
GTTTAGCACGTCAGCTAT
ATAGCTGACGTGCTAAACGGTAGGAGGTGTGGTTGATTGGATTGATTGGATCTTTTCATATGACGTTAATGATACCACGGTGTTTTGA
ATGTTTGCGTGTAGTTA
ACGCGGTTGTTTCCTGCCGCTCCGTGATATTTACTAATGCTTATTCCTAAAACGTCCCATGGTCGTTTTAGGAATAAG
CTTATTCCTAAAACGACCATGGGACGTTTTAGGAATAAGCATTAGTAAATATCACGGAGCGGCAGGAAACAACCGCGT
TACGACATATGGGCCGGACAAGTTGTGGAGGCTGCCAGAAAAACATACGTCGATTTTTTTGAACATGTTTTTGATCGCCG
CGGCGATCAAAAACATGTTCAAAAAAATCGACGTATGTTTTTCTGGCAGCCTCCACAACTTGTCCGGCCCATATGTCGTA
TGTGATCAAAACAAACGGTTTGTAAACATGCAAAGTTCTTGTCCAACATCGGAACTCGACGGACCTATCACCTATTCG
CGAATAGGTGATAGGTCCGTCGAGTTCCGATGTTGGACAAGAACTTTGCATGTTTACAAACCGTTTGTTTTGATCACA
CGAGGCAGTTCAGAGACTCTGCGATGCATTAACGCTGGATATGGGATTATGGAAGTCCATCCTGACC
GGTCAGGATGGACTTCCATAATCCCATATCCAGCGTTAATGCATCGCAGAGTCTCTGAACTGCCTCG
CCCTTTGTGTTGTACAGAGGCTTAGTATTTATGTAACCCCCCCTATGGATGTCCATATATGGTATTAATGGGTTATAAACTTTCAAAAT
TTACCGCCCCGTTTATCCGCGGCCACGATCCCGGGGACAGTTCCGATAGCGCTAATTGTAGC
CGTCATTTATTACCACATCCTTTAATCCCGCCTTTATCTCCCTAAAACGGA
CTCCGTTTTAGGGAGATAAAGGCGGGATTAAAGGATGTGGTAATAAATGACG
CTTAGGGGATGTCTTCCTCTTCCGCGATAATTTCACCACGCCCACATACCCACTCCCAATAAAAGCCCTG
CAGGGCTTTTATTGGGAGTGGGTATGTGGGCGTGGTGAAATTATCGCGGAAGAGGAAGACATCCCCTAAG
ATATGTTTCCCTGACGATAGATGGCCATTCCCGTTGAGTCTACAGTTATAATTGTATACGACTCTTCACGTCCCATCCAC
GTGGATGGGACGTGAAGAGTCGTATACAATTATAACTGTAGACTCAACGGGAATGGCCATCTATCGTCAGGGAAACATAT
GTTCTAAAACACTGTCAGAAACACTTGGCGTGTTAAACACTGTACGCAGAAAG
CTTTCTGCGTACAGTGTTTAACACGCCAAGTGTTTCTGACAGTGTTTTAGAAC
TCACTGAAACACCTTACCAAAAAAAGCTCTGCCGGTAAAGCGGGTAAAGGTATTCCAAACAAGATGAAGAAGTCCTAGTTTGTTGG
GAGGGGGAAGGAAATGCCTTAAACATCCACGGTCTGCTTTATTACCAACTGTATGTAAATTATGA
CCAATAGCAACACTCAGGTATTTTTAAAATGCACGTTTAATGATCATAATTTACATACAGTTGGTAATAAAGCAGACCGTGGATGTTT
AAGGCATTTCCTTCCCCCTCCCAACAAACTAGGACTTCTTCATCTTGTTTGGAATACCTTTAC
CTTCTTGAAGCGTTTCCAGATTGTCCCGTGTGGGACAAGGTCTGTCCCTTACCCCCCTGGGGGGTATTTTGGGTTG
CAACCCAAAATACCCCCCAGGGGGGTAAGGGACAGACCTTGTCCCACACGGGACAATCTGGAAACGCTTCAAGAAG
CGTCTGACCACCACACTCCCGGAGAAATAACTTTACATGCGTATGGATTTTTCGGAAGCCGCGGGGGTTGTAAG
CTTACAACCCCCGCGGCTTCCGAAAAATCCATACGCATGTAAAGTTATTTCTCCGGGAGTGTGGTGGTCAGACG
GGAAGAAACTACACTATCGGCATTACGCGTGGCCAATGAGGTCAATGAGCTACGCATTGAATACG
CGTATTCAATGCGTAGCTCATTGACCTCATTGGCCACGCGTAATGCCGATAGTGTAGTTTCTTCC
GTTAGAACACACGCCGGTACAATTACCCCGCGACGGTACACCCCGAGACGTATGTTCTGT
ACAGAACATACGTCTCGGGGTGTACCGTCGCGGGGTAATTGTACCGGCGTGTGTTCTAAC
TTGTCGCGTCACCGCCTTAAGTTTTGAGACGGTGACGCGACAAGATGAGCGAGTGTACCGATGTTGTGGAGAACTTATCCATTGCCG
TATTAACCTACGAT
ATCGTAGGTTAATACGGCAATGGATAAGTTCTCCACAACATCGGTACACTCGCTCATCTTGTCGCGTCACCGTCTCAAAACTTAAGG
CGGTGACGCGACAA
TATCAGAATTATTTGCAGATACTGTAAATTTAATTAAAGATGAGGCGTTCGTA
TACGAACGCCTCATCTTTAATTAAATTTACAGTATCTGCAAATAATTCTGATA
GTTCACTTTGGGTGCGGTAATGCTCCTGGCTACGTTAATTGCCGCCTCTTCTGAATATACCGGGA
TCCCGGTATATTCAGAAGAGGCGGCAATTAACGTAGCCAGGAGCATTACCGCACCCAAAGTGAAC
GCCCCGACACCGGGGGAATTATCGGGGTTCTGGATCGCGGCAGGGAAAATGATTTCTGTCTCTGGCGCCCCGGTTCCCCCG
CGGGGGAACCGGGGCGCCAGAGACAGAAATCATTTTCCCTGCCGCGATCCAGAACCCCGATAATTCCCCCGGTGTCGGGGC
ATAAGGTAAAATCTAGGACATTATCCGGGCGGAATAGAGTCATCCGATAGATTA
TAATCTATCGGATGACTCTATTCCGCCCGGATAATGTCCTAGATTTTACCTTAT
CCATATACGAGTGCGCGGCGGTTCCCAACGTAGCACAGGCCACGAGCGTTCCCAGGGACGG
CCGTCCCTGGGAACGCTCGTGGCCTGTGCTACGTTGGGAACCGCCGCGCACTCGTATATGG
AACAACCCCTGTGTTTGTGCCGCGGTGGGAACGTCGCATACACCGTGTGTACCCGCGCGGGCCCCAAGACCCGCGTCCC
GGGACGCGGGTCTTGGGGCCCGCGCGGGTACACACGGTGTATGCGACGTTCCCACCGCGGCACAAACACAGGGGTTGTT
TATTCTTAATGATAAGACGATGTAGTGATTGGACGAGACTCGAGGCGGGAAGTTCATGGACCATAGTATGCGTTTAAGGAGAGACC
GCTGGT
ACCAGCGGTCTCTCCTTAAACGCATACTATGGTCCATGAACTTCCCGCCTCGAGTCTCGTCCAATCACTACATCGTCTTATCATTAAG
AATA
TTCCAAATTTTAGCTGGGTATTAAATCCCCGGAGAAACAGCTTTAATAAATAACGAACTGTCAAACAGTTAGCCGCTAACCGA
TCGGTTAGCGGCTAACTGTTTGACAGTTCGTTATTTATTAAAGCTGTTTCTCCGGGGATTTAATACCCAGCTAAAATTTGGAA
CTCCAGGCACCCCGTCCGACCCTCTAGTTCCCGCGTTGCATCTGGG
CCCAGATGCAACGCGGGAACTAGAGGGTCGGACGGGGTGCCTGGAG
CTCTAACACAACCCAACTGTGTGTTGACATTGTTCTAACCAACTGACCCGCAAAATATAAAAAC
GTTTTTATATTTTGCGGGTCAGTTGGTTAGAACAATGTCAACACACAGTTGGGTTGTGTTAGAG
ACGCAAGTTTGCTTTACCCTCGCCTTCACCACCCCCGCCACCTTCCGGCCATTGTAATAACTTTAATTGC
GCAATTAAAGTTATTACAATGGCCGGAAGGTGGCGGGGGTGGTGAAGGCGAGGGTAAAGCAAACTTGCGT
CTGCGTGCGTTGGTTTTACCCCATTCGGCGTGGCGATGCCTAACAAAGGTGGAAAAGGCAGAAAACCCG
CGGGTTTTCTGCCTTTTCCACCTTTGTTAGGCATCGCCACGCCGAATGGGGTAAAACCAACGCACGCAG
CTGTTCTAACGATAATACGAACGGAGACATGCTTCGTAAGCAGTTTGAAAGGGTCTC
GAGACCCTTTCAAACTGCTTACGAAGCATGTCTCCGTTCGTATTATCGTTAGAACAG
CTCACGTTTCTAATGTAACAGCGTTTGACATAGCAACTACGGCTATTTTATTTG
CAAATAAAATAGCCGTAGTTGCTATGTCAAACGCTGTTACATTAGAAACGTGAG
CAATAAAAGCCCTGTAGAGCGCATTGGCATCTTACTTGAGATTTGGATACGCTCGGCCGACTTGGTCTGTTTCACGCTTCCTTAAACA
ACATGGCTATGCCATTTG
CAAATGGCATAGCCATGTTGTTTAAGGAAGCGTGAAACAGACCAAGTCGGCCGAGCGTATCCAAATCTCAAGTAAGATGCCAATGC
GCTCTACAGGGCTTTTATTG
GGAGAAACATTAAGCCGGGCTATTATTAACGGCTGTGGTCTGCTTCAGCGAATTTGCTTCCCCGAGACACAAAGATTATCGGG
CCCGATAATCTTTGTGTCTCGGGGAAGCAAATTCGCTGAAGCAGACCACAGCCGTTAATAATAGCCCGGCTTAATGTTTCTCC
GTGTATAAAAGCCTCGTAGGCCTCGGTAGATATGGCGCATAGCGCGGCGGCGAAGTACCGTTCGGCGTCGTTCAT
ATGAACGACGCCGAACGGTACTTCGCCGCCGCGCTATGCGCCATATCTACCGAGGCCTACGAGGCTTTTATACAC
CCCGATATTGTTTCTTGGGGGTTACGTTTACTGCATATGGATCCCCACCCAATTGAAAATGCATG
CATGCATTTTCAATTGGGTGGGGATCCATATGCAGTAAACGTAACCCCCAAGAAACAATATCGGG
ATTAAAATAATGGCGGTACACAGACCGCTCCTTTGACATAAGTTTTCCTCGAATGGTTTGACGGCGGCATGGCGTTCTGG
CCAGAACGCCATGCCGCCGTCAAACCATTCGAGGAAAACTTATGTCAAAGGAGCGGTCTGTGTACCGCCATTATTTTAAT
TCTTCCCCCCGAGGGGGTCGATGGGACACTTCCATTAAAGTAGCGCGTAAGCACTGCGTCTGGGT
ACCCAGACGCAGTGCTTACGCGCTACTTTAATGGAAGTGTCCCATCGACCCCCTCGGGGGGAAGA
CGTACTACGCGTACCCCGGGATGCATCGCAGATGTCATTTCGTTTGGTGTTGAGTCATGGAACAAACCAACCCCCTGAAATCTTGC
GCAAGATTTCAGGGGGTTGGTTTGTTCCATGACTCAACACCAAACGAAATGACATCTGCGATGCATCCCGGGGTACGCGTAGTACG
TCCGATTCTATTGTTAATAGAATACGTTTAATGTCGTCTTGGGTTAATTTGGATGCTAACTCTTCCGGATGCGGCTCC
GGAGCCGCATCCGGAAGAGTTAGCATCCAAATTAACCCAAGACGACATTAAACGTATTCTATTAACAATAGAATCGGA
ACTTGGACGTGGGTTGGATATGAATTTTTCAAAGCATGCTACGGTCGATATATGCGCATATCCCGAAGAGAGTTTGGATTACC
GGTAATCCAAACTCTCTTCGGGATATGCGCATATATCGACCGTAGCATGCTTTGAAAAATTCATATCCAACCCACGTCCAAGT
TTAAATTTTGCTTCACAAGCAGCCCCTTGTATATGCTCATTATAACACGCCTGGCGAGAGAAAGTGCATTCCCGTGGTCAAGACCATG
TGAA
TTCACATGGTCTTGACCACGGGAATGCACTTTCTCTCGCCAGGCGTGTTATAATGAGCATATACAAGGGGCTGCTTGTGAAGCAAAA
TTTAA
CTATGAGCCGTCGGTACGACCGGGCCCAAAAACACTTTATCCTACAGAGTCTCCGCAGAGCCTT
AAGGCTCTGCGGAGACTCTGTAGGATAAAGTGTTTTTGGGCCCGGTCGTACCGACGGCTCATAG
CAAATAACCCAAGTGCTTTAGCGAGCACCATTTTGGATCAACGTGTTAAAGCTCGTATTCTCGGCGACGTTATCTCCGTTTC
GAAACGGAGATAACGTCGCCGAGAATACGAGCTTTAACACGTTGATCCAAAATGGTGCTCGCTAAAGCACTTGGGTTAATTG
CAGACACGGCTCTCGTATCGTGGTTATCTGTAGTCGGGGAAGCTCAAACCGCAGGTTACTTATCCAGACGAGAGTTCGATGAATTAT
CAC
GTGATAATTCATCGAACTCTCGTCTGGATAAGTAACCTGCGGTTTGAGCTTCCCCGACTACAGATAACCACGATACGAGAGCCGTGT

CTG
GGCCGTGTTTGCATCGTCGCGCACCTTTGGGCCAGCGAGTTTTGCTCGTATCGAGACTGCGTTTGCCAACC
GGTTGGCAAACGCAGTCTCGATACGAGCAAAACTCGCTGGCCCAAAGGTGCGCGACGATGCAAACACGGCC
TGGGCTTCTCGAAATTTATCGGGATCAAACCCGCTATTTACCGACGGTTCAGTGTTTTGTGAGTCGCCAATTTCTTC
GAAGAAATTGGCGACTCACAAAACACTGAACCGTCGGTAAATAGCGGGTTTGATCCCGATAAATTTCGAGAAGCCCA
CTCCGATGACGATCCCCACGACGAGGAAGAGGATGAAGACGAAACAAACTCACGACTCT

AGAGTCGTGAGTTTGTTTCGTCTTCATCCTCTTCCTCGTCGTGGGGATCGTCATCGGAG
CCCTTTCAAACTGCTTACGAAGCATGTCTCCGTTCGTATTATCGTTAGAA
ATCTAACGATAATACGAACGGAGACATGCTTCGTAAGCAGTTTGAAAGGG
TATCATTAAGAATATTTACACGGTGACGACACGGGGAGGAAATATGCGGTCGAGGGGGGG
CCCCCCCTCGACCGCATATTTCCTCCCCGTGTCGTCACCGTGTAAATATTCTTAATGATA
ATAAATGTTAGTCTTGAATCTGTCTTTACGCGTTTGCGGCGTCCCGTAAT
ATTACGGGACGCCGCAAACGCGTAAAGACAGATTCAAGACTAACATTTAT
AGCAGGATTGCTAGGGCGTCATATTCTCACGGCCGTTGTTTACTGTTGGTG
CACCAACAGTAAACAACGGCCGTGAGAATATGACGCCCTAGCAATCCTGCT
AGCTTGTTCGTGTTATGTATTAAATAAGCCGGTTTTCATCACAATGGA
TCCATTGTGATGAAAACCGGCTTATTTAATACATAACACGAACAAGCT
TGCCCCTTGCCCTTCCCAAGTACACCAGGGTGATAAAATCCACTTGAAAGTTTGTATCG
CGATACAAACTTTCAAGTGGATTTTATCACCCTGGTGTACTTGGGAAGGGCAAGGGGCA
CTCCGCAACCCCTCCTGCGTTCACTCCGTACATACGTTCTCCGTGA
TCACGGAGAACGTATGTACGGAGTGAACGCAGGAGGGGTTGCGGAG
AAATTCTAACAACTCTGGAGTCTCCAAATGATGTAACCTACACAACACCCGGTTCTACCAACGCACTGTTCTTTAAGACGTCCACAC
GTGTGGACGTCTTAAAGAACAGTGCGTTGGTAGAACCGGGTGTTGTGTAGGTTACATCATTTGGAGACTCCAGAGTTGTTAGAATTT
TCCGATTACTGCTGTGGTTATTTCCAACGAGGCAAAGTTGTCGACGTTAAGAAAAATATTGCCACATTTCAC
GTGAAATGTGGCAATATTTTTCTTAACGTCGACAACTTTGCCTCGTTGGAAATAACCACAGCAGTAATCGGA
GAAGTTGGATTTATTTGAATGTTGTTCATACGATTAATAACAATTGAACGGGGGGTTACTTGAATAGACGCGGTTTCTGTACGTTT
AAACGTACAGAAACCGCGTCTATTCAAGTAACCCCCCGTTCAATTGTTATTAATCGTATGAACAACATTCAAATAAATCCAACTTC
CACAAAACAGATTTGAAGGTCGTGTATGTGCTGGCCTGTGG
CCACAGGCCAGCACATACACGACCTTCAAATCTGTTTTGTG
TTATAGTGACACTAATTTATTTATGGGGGTTGGGAATAGAGAAGCAGAATCTGTCTAGAATAGGTCCGATTAAC
GTTAATCGGACCTATTCTAGACAGATTCTGCTTCTCTATTCCCAACCCCCATAAATAAATTAGTGTCACTATAA
GTCCATTTGAGACTCCCCACTAGAAAAAGAATCCAATTCCCTTTGATCCATGCTCT
AGAGCATGGATCAAAGGGAATTGGATTCTTTTTCTAGTGGGGAGTCTCAAATGGAC
ATGACATGGTTAAGACCAACGGTTGGCTTAAAACGTACGTTTAAC
GTTAAACGTACGTTTTAAGCCAACCGTTGGTCTTAACCATGTCAT
CAGATGCACAATCTGAGGAATGTACATCTGCTTCTTCCGCCTCGGCAGCAGCCGTCTCCAGGGAATCCAAAACCTTGGCCATGCGCGTTAGT
ACTAACGCGCATGGCCAAGGTTTTGGATTCCCTGGAGACGGCTGCTGCCGAGGCGGAAGAAGCAGATGTACATTCCTCAGATTGTGCATCTG
GCATACCCCGGTCCCGTCGGACCAGGCTCGCAAGGCGTACTGTACCCCCGAAACCATCGCCCGTCTGGTCGAC
GTCGACCAGACGGGCGATGGTTTCGGGGGTACAGTACGCCTTGCGAGCCTGGTCCGACGGGACCGGGGTATGC
CCATCAAATTCACGAACACTGCGCGCGTAGGCCCCGCAAGGCGATATAAAAAGACGCTCTGCTG
CAGCAGAGCGTCTTTTTATATCGCCTTGCGGGGCCTACGCGCGCAGTGTTCGTGAATTTGATGG
TAATCTATCGGATGACTCTATTCCGCCCGGATAATGTCCTAGATTTTACCTTATGTGTTATTTTAATGTATCTGGA
TCCAGATACATTAAAATAACACATAAGGTAAAATCTAGGACATTATCCGGGCGGAATAGAGTCATCCGATAGATTA
CACCGGGAAGACATGGGCGGTATCATCCAATAATAACTGCGTGTTTTTGGATCGAGTGGCCTTACTTG
CAAGTAAGGCCACTCGATCCAAAAACACGCAGTTATTATTGGATGATACCGCCCATGTCTTCCCGGTG
GAGCGTTTTAAGTTTGGGGTACGTTTTTAAAGCTTGATTGGCAAACGTTACCGCAAATCAATCTTAGATCCAAGGGATAATGGCCGTTGGGCAGGCCTCCAAAAGGCAATTTGAGCGTTTTC
AGTCCGCTTGAAAACGGTCTAAGTATTTG
TTATACACCCAATCACACGTCACGTATTAAGCTTTACAGTCCCCCGTTATCTGATATAATCACTTTTCTTAACACGTCATCGGGAAAACAGATGTTTATATTATACCTCTCGCGGTCATTTATG
GCAAATACTTAGACCGTTTTCAAGCGG
TATCTTTTAAACACCGATTCGGAATAGTTTGGCTACAAAACATATCACCTGTAATTGATCCCATCTTGGAAACGGCAGTAAATAC
GTATTTACTGCCGTTTCCAAGATGGGATCAATTACAGGTGATATGTTTTGTAGCCAAACTATTCCGAATCGGTGTTTAAAAGATA
TACCCCCGCGCACCGGGCTCTCATGAACGACGCCGAACGGTACTTCGCCGCCGCGCTAT
ATAGCGCGGCGGCGAAGTACCGTTCGGCGTCGTTCATGAGAGCCCGGTGCGCGGGGGTA
CTTTAATGTTGGCAGCAAGTCCTCTTTACGCTCACCAAACTTTAACATCCTTTTGGAAAGACCGCGATG
CATCGCGGTCTTTCCAAAAGGATGTTAAAGTTTGGTGAGCGTAAAGAGGACTTGCTGCCAACATTAAAG
CTTGCTGTTCTCATTTCTAGGTTTTGTCTTAAATACACCCGCCATGAGCATCTC
GAGATGCTCATGGCGGGTGTATTTAAGACAAAACCTAGAAATGAGAACAGCAAG
GCACTTTTTTCTTAGAGCTTTAACTGGCACGTCTTCCAGTGAGAT
ATCTCACTGGAAGACGTGCCAGTTAAAGCTCTAAGAAAAAAGTGC
GCCCAACGCGGTGAGCGCTTTCGCGGCCGTATATTCCGCGGTTTCTGCACCCGCGTATCGCTCGATGGCACGCTGGGGTCTCTTTGG
CCAAAGAGACCCCAGCGTGCCATCGAGCGATACGCGGGTGCAGAAACCGCGGAATATACGGCCGCGAAAGCGCTCACCGCGTTGGGC
TTCGGCGCCTGGTTTAAAGGCGGGTAAGGGGGTTTGATTATTTAGGACGTTAGCCAAAAACGGTAA
TTACCGTTTTTGGCTAACGTCCTAAATAATCAAACCCCCTTACCCGCCTTTAAACCAGGCGCCGAA
CTGCTAACCATAGACTGTTCTACTGACTTTTCATCATACGCATTTACGCACCGGGAT
ATCCCGGTGCGTAAATGCGTATGATGAAAAGTCAGTAGAACAGTCTATGGTTAGCAG
GTGTTATTTTAATGTATCTGGAAGGCATTGGGCCCTCCGGGGCA
TGCCCCGGAGGGCCCAATGCCTTCCAGATACATTAAAATAACAC
CGTATTCAATGCGTAGCTCATTGACCTCATTGGCCACGCGTAATGCCGATAGTGTAGTTTCTTCC
GGAAGAAACTACACTATCGGCATTACGCGTGGCCAATGAGGTCAATGAGCTACGCATTGAATACG
TAATAGCCCGGCTTAATGTTTCTCCAAACATATAAAGCTGCC
GGCAGCTTTATATGTTTGGAGAAACATTAAGCCGGGCTATTA
ACCCTAGATGTAGTTACAGCGAGGTTGATCCTTGGCATGCGGTTCTAGAAACAACCTCGA
TCGAGGTTGTTTCTAGAACCGCATGCCAAGGATCAACCTCGCTGTAACTACATCTAGGGT
TGTACGTACCAAACGAAGCCAGTTTTTCATTGGTGTATATGCGTATGTTGGAGTTACCAGAAGGTTTGCAGGAG
CTCCTGCAAACCTTCTGGTAACTCCAACATACGCATATACACCAATGAAAAACTGGCTTCGTTTGGTACGTACA
ATGTCATCTGGTGCTAACACACGCGCATCCGTCGCCATTGTCCCGTGTTCCATTTCGG
CCGAAATGGAACACGGGACAATGGCGACGGATGCGCGTGTGTTAGCACCAGATGACAT
GACGGACATCGGTTTTGTCTGGAAAATTTACACACGGGGTCTGCAAGTCAAT
ATTGACTTGCAGACCCCGTGTGTAAATTTTCCAGACAAAACCGATGTCCGTC
AGGATGGACTTCCATAATCCCATATCCAGCGTTAATGCATCGCAGAGTCTCTGAACTGCCTCGGGGGTTAATTTGCGCGCT
AGCGCGCAAATTAACCCCCGAGGCAGTTCAGAGACTCTGCGATGCATTAACGCGGGATATGGGATTATGGAAGTCCATCCT
GCCACGATCCCGGGGACAGTTCCGATAGCGCTAATTGTAGCA
TGCTACAATTAGCGCTATCGGAACTGTCCCCGGGATCGTGGC
TCGGCGGACTACGTCCTGCTACATCGGCAGGAATTGCACGAGGGCAA
TTGCCCTCGTGCAATTCCTGCCGATGTAGCAGGACGTAGTCCGCCGA
TTTTATTAACCTCACCATTTTGCGTTTAACGGGATTTGTGG
CCACAAATCCCGTTAAACGCAAAATGGTGAGGTTAATAAAA
AAAGAGAAAATAGGTTTCAGGGTATGCGTACCAATCCCAAATCCCTATGCGTTAGTGGGG
CCCCACTAACGCATAGGGATTTGGGATTGGTACGCATACCCTGAAACCTATTTTCTCTTT
TTGCGCTACAATATCACCGCAACGTTATGCACACAGCGTGCCTTTACCCCTCCTCACCCACCAACAGGCTTGGCGGCTTTTAATATTACCACTCCGTTTTAGGG
CCCTAAAACGGAGTGGTAATATTAAAAGCCGCCAAGCCTGTTGGTGGGTGAGGAGGGGTAAAGGCACGCTGTGTGCATAACGTTGCGGTGATATTGTAGCGCAA
CCGCGCGTTTGCGGGGCGACCATCGGGGGGGACTTGCATTACCCTATCCCAGTATTGTCTGTACGCCTCTAATGG
CCATTAGAGGCGTACAGACAATACTGGGATAGGGTAATGCAAGTCCCCCCCGATGGTCGCCCCGCAAACGCGCGG
ACCCCCGGAAGAAACCGACATTCCACCCGAAGAGTTAATTCGGCGTGTATAT
ATATACACGCCGAATTAACTCTTCGGGTGGAATGTCGGTTTCTTCCGGGGGT
ACTCTTTTAATCTGCATTCGCACTTCCCGTTTTTTCACTGTATGGGTTTTCATGTTTTGGCATGTGTCCAACCACCGTTCGCACTTTCTTT
AAAGAAAGTGCGAACGGTGGTTGGACACATGCCAAAACATGAAAACCCATACAGTGAAAAAACGGGAAGTGCGAATGCAGATTAAAAGAGT
TGTTTTCTCGGTTTGCGCGTTCCTTTTCCAGCGATGATAGAACGCGTAAATCTTATGATGGTAGTTACCAAAGTTTTAA
TTAAAACTTTGGTAACTACCATCATAAGATTTACGCGTTCTATCATCGCTGGAAAAGGAACGCGCAAACCGAGAAAACA
CCCCCCGCACATTGCCGATCAAAAGCTCGCTTCCGTTGAAATTAACATTATCGAC
GTCGATAATGTTAATTTCAACGGAAGCGAGCTTTTGATCGGCAATGTGCGGGGGG

GTTATTGGCTCGAGATCCATATGGACCTGCCGTGGACATATGGAGTGCCGGGATTGT
ACAATCCCGGCACTCCATATGTCCACGGCAGGTCCATATGGATCTCTAGCCAATAAC
ATGAAACTGGTCGCGTGGTAGACGATCATAGCAAAATTGATA
TATCAATTTTGCTATGATCGTCTACCACGCGACCAGTTTCAT
TCATCGCGTTCAACCTTTGGGTTACAGATACCAACAGCCTGAAGATCTGG
CCAGATCTTCAGGCTGTTGGTATCTGTAACCCAAAGGTTGAACGCGATGA
ACCGGATAAAAGCGTACTGTTTTTATTTAAATTTACACGCTCGACGTTGCCCCGGTTCGGTGATCACCGGGA
CCCGGTGATCACCGAACCGGGGCAACGTCGAGCGTGTAAATTTAAATAAAAAACAGTACGCTTTTATCCGGT
TGTTATATTTGAACCTTCCGGATCTAATATAACAAGGTGGAAGTCGCTTATACGCCCCGATCTTCATATTGAAGTTTGGTT
AACCAAACTTCAATATGAAGATCGGGGCGTATAAGCGACTTCCACCTTGTTATATTAGATCCGGAAGGTTCAAATATAACA
CAGTCAACCCATCCGAGTGAATCTTAAACCTGACCCGTTGGCGTCGTTTACACAAGTTATACCGCCACTGGCGTTGGAAACAACGTG
CACGTTGTTTCCAACGCCAGTGGCGGTATAACTTGTGTAAACGACGCCAACGGGTCAGGTTTAAGATTCACTCGGATGGGTTGACTG
CGAAAAGGTGTTTGTATTTAAATCTTGAGAAGAGATGGGTGTTCGAGCGTGTAAATGCATTTGCACATCGTTG
CAACGATGTGCAAATGCATTTACACGCTCGAACACCCATCTCTTCTCAAGATTTAAATACAAACACCTTTTCG
ATTGACGTTGGTCCACATTTACAATTTTATGTCTGTCATCGCCGTTTAAC
GTTAAACGGCGATGACAGACATAAAATTGTAAATGTGGACCAACGTCAAT
CATTGCTTGATGTTGAGGCCACACTTGTTGCCCTCCGCCAACAACATTTAGATC
GATCTAAATGTTGTTGGCGGAGGGCAACAAGTGTGGCCTCAACATCAAGCAATG
GGGCCGCGCGCGTCTGTTATTCCAGATGATTTGCTTAGACGACATTTAAAAACGGGTCCTGCGGTCTCAGCG
CGCTGAGACCGCAGGACCCGTTTTTAAATGTCGTCTAAGCAAATCATCTGGAATAACAGACGCGCGCGGCCC
CCCATTAGATCCTGACCGTCCTCGCATACGTAGTCGTCTTGTGTTAGCTCTTCGCCAACATCTTCCGTTCTGGGTTCTGGTT
AACCAGAACCCAGAACGGAAGATGTTGGCGAAGAGCTAACACAAGACGACTACGTATGCGAGGACGGTCAGGATCTAATGGG
TACAAAACCCTGACCAGAAAGCTTACACGCAACTCAGGCAAAGTATTC
GAATACTTTGCCTGAGTTGCGTGTAAGCTTTCTGGTCAGGGTTTTGTA
CGCCGATGGGGAGGGGGCGCGGTACCCCGCCGATGGGGAGGGGGCGCGGTACCCCGCCGATGGGGAGGGGGCGCGGTACCCC
GGGGTACCGCGCCCCCTCCCCATCGGCGGGGTACCGCGCCCCCTCCCCATCGGCGGGGTACCGCGCCCCCTCCCCATCGGCG
CCCATCCGGTTACCAAAAAAACCCGCAAGCGACCCCGCGGGCCGCCTTTGG
CCAAAGGCGGCCCGCGGGGTCGCTTGCGGGTTTTTTTGGTAACCGGATGGG
ATACTTAAATCCTTCAAAACACATGTTTACAATACCAAACGTATACAGTCCTTGTATGGGTTCCCTTCGTACAGACCCCACGG
CCGTGGGGTCTGTACGAAGGGAACCCATACAAGGACTGTATACGTTTGGTATTGTAAACATGTGTTTTGAAGGATTTAAGTAT
CAGGGAATGTTTTCGGGCCATGGTATCGTACTCTGCAGGAACGGCTTGTGTTAGATAGGCCCG
CGGGCCTATCTAACACAAGCCGTTCCAGCAGAGTACGATACCATGGCCCGAAAACAATCCCTG
TTGTTGGCGCATGCCATTGGCGTGGGTACTCCTACCCCCGGGTTGTT
AACAACCCGGGTGTAGGAGTACCCACGCCAATGGCATGCGCCAACAA
CCAGGGTTCCTGCAGGGTCCACTGGAGGAGCCACACAATATTCGCGAATTCCGAT
ATCGGACTTCGCGAATATTGTGTGGCTCCTCCAGTGGACCCTGCAGGAACCCTGG
TACCGCGACCCTGGGATGTTTTACAGGCGCGTTTGTTTGTCTCGGTTATAAGTATGCAGTCGGGTCATTATAACCGGAGGCAATCCCGCCG
CGGCGGGATTGCCGCCGGTTATAATGACCCGACTGCATACTTATAACCGAGACAAACAAACGCGCCTGTAAAACATCCCAGGGTCGCGGTA
CCAAGGCCGAGTTGAGACGGGAAAATTAGAAGCTGTTTTTCCAGCGGCAAAAATCATCCGGTTTGGGTATGTCTTATAGCAATTAAAGTTATTACAATGGCCGGAAGGTGGCGGGGGTGGTGAA
GGCGAGGGTAAAGCAAACTTGCGGGAG
GGGGGCGCGGTACCCCGCCGATGGGGAGGGGGCGCGGTACCCCGCCGATGGGGAGGGGGCGCGGTACCCCGCCG
CGGCGGGGTACCGCGCCCCCTCCCCATCGGCGGGGTACCGCGCCCCCTCCCCATCGGCGGGGTACCGCGCCCCC
CCCGCAAGTTTGCTTTACCCTCGCCTTCACCACCCCCGCCACCTTCCGGCCATTGTAATAACTTTAATTGCTATAAGACATACCCAAACCGGATGATTTTTGCCGCTGGAAAAACAGCTT
CTAATTTTCCCGTCTCAACTCGGCCTTGGAG
GTTAAGGTCCCACCCCCGGCGCGGAAACCCGGTGGACCTGTACCCGGCCCGGTTGAGCAATTGTACC
GGTACAATTGCTCAACCGGGCCGGGTACAGGTCCACCGGGTTTCCGCGCCGGGGGTGGGACCTTAAC
CCCCTTACACGAAGCAGCGGTTAATGGGGCGGGTGCCGGGATTGACCGTGATGGTGC
GCACCATCACGGTCAATCCCGGCACCCGCCCCATTAACCGCTGCTTCGTGTAAGGGG
ACATCGGCATTAACAACACGTACGTGCCTAGGAACGCGAGAT
ATCTCGCGTTCCTAGGCACGTACGTGTTGTTAATGCCGATGT
GGTTCCGCGGCAGGGTCGGGTTCCGGCAGGGCGTGGCGGCAAAACACCCTCG
CGAGGGTGTTTTGCCGCCACGCCCTGCCGGAACCCGACCCTGCCGCGGAACC
CCCGTCCAGCCCGCGCAGCCCGTCCAGCCCGTCCAGCCCGCGCAGCCCGTCC
GGACGGGCTGCGCGGGCTGGACGGGCTGGACGGGCTGCGCGGGCTGGACGGG
GGGACGACGGGGACGCACCGGGGTCATCCTTTGGGGTGAGCGTCGTGTCGGTGGCCCCGGGCGTGCTGCGAGACCGCC
GGCGGTCTCGCAGCACGCCCGGGGCCACCGACACGACGCTCACCCCAAAGGATGACCCCGGTGCGTCCCCGTCGTCCC
GGGATTTTTTGCCGGGAAACCCCCCCCGCCAGCCTTTAACAAAACCCGCGCCGTTTGCGTCCACCCCTCGTTTACTGCTCGGATGGCCACCGTGCAC
GTGCACGGTGGCCATCCGAGCAGTAAACGAGGGGTGGACGCAAAAGGCGCGGGTTTTGTTAAAGGCTGGCGGGGGGGGTTTCCCGGCAAAAAATCCC
CAAACCGTCGGTCTCCTCCGCCCGGACGCCGAGCGGCGATTTCCGCCAAGGCGCCGGGATCAAAGCTTAGCGCAGGGCGCCAGGCCGTGGGAAACAATGGGTCGTCGACCAGACGGGCGATG
GTTTCGGGGGTACAGTACGCCTTGCGAGC
GTCCAACAGGTCCATTAACTCCAAAGTATCAGGCG
CGCCTGATACTTTGGAGTTAATGGACCTGTTGGAC
TGAAACGATCTCATGTTCCCGTCCCACCATGACCCCGTTCTCTCCAAATAGTAGATCGTCAGGCTGACTCGAGGTGACCACCCGGGCCCTGTGTTCGGCGGCCGCGGCGGCCGCGTCCAACAGG
TCCATTAACTCCAAAGTATCAGGCGAC
CCCCAGCGCGATGGATACGCCGCCGATGCAGCGCTCTACACCCCAACGCGCGGGGTCGCCTGATACTTTGGAGTTAATGGACCTGTTGGACGCGGCCGCCGCGGCCGCCGAACACAGGGCCCG
GGTGGTCACCTCGAGTCAGCCTGACGAT
GCGGCGGCCGCGTCCAACAGGTCCATTAACTCCAAAGTATCAGGCGACCCCGCGCGTTGGGGTGTAGAGCGCTGCATCGGCGGCGTATCCATCGCACCGGGGTGAATTTAGACGTACC
CGAGTTTTC
GAAAACTCGGGTACGTCTAAATTCACCCCGGTGCGATGGATACGCCGCCGATGCAGCGCTCTACACCCCAACGCGCGGGGTCGCCTGATACTTTGGAGTTAATGGACCTGTTGGACGC
GGCCGCCGC
CGATACGGAGGGAATTGAAACGATCTCATGTTCCCGTCCCACCATGACCCCGTTCTCTCCAAATAGTAGATCGTCAGGCTGACTCGAGGTGACCACCCGGGCCCTGTGTTCGGCGGCCGCGGCG
GCCGCGTCCAACAGGTCCATTAGATCG
AATGGACCTGTTGGACGCGGCCGCCGCGGCCGCCGAACACAGGGCCCGGGTGGTCACCTCGAGTCAGCCTGACGATCTACTATTTGGAGAGAACGGGGTCATGGTGGGACGGGAACATGAGAT
CGTTTCAATTCCCTCCGTATCGAGATCG
CCAGTCGTTTTTCTTCACATGAATACATATCTCTTAGTTCGTCCATAAGGTCTAAGTTGGGTCTAAGTAACTCACCCGAGGTGGTGACCTTACTAAACATATTATTATAAATTGGAGAGA
AACCCTCACAGCACTCCGTTACCAGTGCAGA
TATTTAGATTTACCATGGTGTACCCTGCGAGATGATATTTGCGCTTATGGGTTATACAACTCGCAGTTCTTAGCGTTAATGCCCACAGTTTCATCTGCACAGGTAACGGAGTGCAGTGAG
GGTTTCTCTCCAATTTATAATAATATGTTTA
CTCGCCGGTTTTATTCACGGAAGCGTTGTAGCAGACGAGCATATTACAGATTTCCAACAAACACTAGGATGTCTCGCTTTAGTGGGTGGACTGGCATACCAATTAGTGGAAACGTACGC
TCCTACTACCGAGTATGTGTTAACATATACAC
TCTCGTTTACTGTCCGTGTATATGTTAACACATACTCGGTAGTAGGAGCGTACGTTTCCACTAATTGGTATGCCAGTCCACCCACTAAAGCGAGACATCCTAGTGTTTGTTGGAAATCTG
TAATATGCTCGTCTGCTACAACGCTTCCGTG
ACCTTTTACTTGGATACTGATGGGGTTCCACTTCGATATAGAGTGTGTTATAAATCAGTTGGGGTTAAACTTGGAACCATGCTATGCAGTCAGGGTGGATTATCTTTA
TAAAGATAATCCACCCTGACTGCATAGCATGGTTCCAAGTTTAACCCCAACTGATTTATAACACACTCTATATCGAAGTGGAACCCCATCAGTATCCAAGTAAAAGGT
GAGGAATGACACCCAAACAGACAAAATATAGAAGATCATAGTCACTGTTAACGTTGAACTGCGCAAGGCGGCGACTTTCTTCCAATGCCGCCCTTACACGCGGTTGGTGCATTAACAT
TCCAAGTCCCCGTTCATATTGCAACATAACACT
CGTTGTGTATTAAAGCATTGTATAAAAACACGCATGCGGGCTTGCTGTTCTCATTTCTAGGTTTTGTCTTAAATACACCCGCCATGAGCAT
ATGCTCATGGCGGGTGTATTTAAGACAAAACCTAGAAATGAGAACAGCAAGCCCGCATGCGTGTTTTTATACAATGCTTTAATACACAACG
GCCGGAACCCCCGGAACATATAGGACGGGCACGTCGGTGAATTGCATCATTGAGGAAGTTGAAGCCAGATCAATATTCCCTTATGATAGTTTTGGACTTTCCACG
CGTGGGTTCTTGGGACTCCAAGGGACCCGCAGCCCAGGTACGCGTCAAAAAGCCTGTGACAAATTCCCCCGGGGCGGTCATATAATTCGGCGCGGATGCATTTATTATCGGGCGACAA
TCCAATAGTCAGAAGTCCGG
CCGGACTTCTGACTATTGGATTGTCGCCCGATAATAAATGCATCCGCGCCGAATTATATGACCGCCCCGGGGGAATTTGTCACAGGCTTTTTGACGCGTACCTGGGCTGCGGGTCCCTTG
GAGTCCCAAGAACCCACG
GAAGCCTTAAAGGCCGAGCTGGTATACACGAGAGCAGTCCATGGTTTTAGACCTCGGGCGAATTGCGTGGTTTTAAGTGACTATATTCCGAGGGTCGCCTGTAATATGGGGACAGTTAATAAAC
CTGTGGTGGGGGTATTGATGGGGTTCG
ACGCAACGTTCCCGTGATAATACCGAACCCCATCAATACCCCCACCACAGGTTTATTAACTGTCCCCATATTACAGGCGACCCTCGGAATAAAGTCACTTAAAACCACGCAATTCGCC
CGAGGTCTAAAACCATGGACTGCTCTCGTGTAT
CCCGTAGCTGCACGCGGAGTTGATTGTAAAGACGGGCGCATTTCCCGTACGTTTATGAACACAACGGTAACATGTCTACAGTCCGCCGGGAGAATGTATGTTGGAGATAGAGCGTACT
CCGCGTTCGAGTGTGCG
CGCACACTCGAACGCGGAGTACGCTCTATCTCCAACATACATTCTCCCGGCGGACTGTAGACATGTTACCGTTGTGTTCATAAACGTACGGGAAATGCGCCCGTCTTTACAATCAACTC
CGCGTGCAGCTACGGG
GGGTATGCGCCAGCTGAGGGATACCGTGGCTTTAATAAAGGAAACCACACCCACGGGATCTGATGAAATAATGGTGACCCCCCTCCGTGTGTTTGTA
GGAATCATACCCAAATATGACTCCGCCCACTCACGGATGCCTGGAGAGTTGGGCAAGGCAGGGGGTGTTATTGCTGAATACCA
TGGTATTCAGCAATAACACCCCCTGCCTTGCCCAACTCTCCAGGCATCCGTGAGTGGGCGGAGTCATATTTGGGTATGATTCC
TCACCCCCGTCTCTTCGCAACTGGATTCGGTTGAAGTTTTCAGCAAGTTTAACATTTCCCCTGAGTGGTATGACCTGTTGTCGGACGAACTTAAAGAGCCG
CGGCTCTTTAAGTTCGTCCGACAACAGGTCATACCACTCAGGGGAAATGTTAAACTTGCTGAAAACTTCAACCGAATCCAGTTGCGAAGAGACGGGGGTGA
GCTATTATAATTCTAACATCAACAGTTGCGGCGGGTTTGACGTGTATTCAGATGATGAGATTAAGCGTTAAACCTATTGACTGTTTTATGG
CCATAAAACAGTCAATAGGTTTAACGCTTAATCTCATCATCTGAATACACGTCAAACCCGCCGCAACTGTTGATGTTAGAATTATAATAGC
CAAGAATATGCCAATGGATTATTTAGTTATGTTTCGGCGGTACGAATTCCACAGCAAAAACAAATGGATTACCCACCCCCAGCCATCCAATGTAATGTTTTATGGATTCGGGATGGCGT

CTCTAATATGAAATATTCTGCTGTCGTTACCC
CTATAGACACGTTGGGAAATGGATAGACGTCAGGGGTAACGACAGCAGAATATTTCATATTAGAGACGCCATCCCGAATCCATAAAACATTACATTGGATGGCTGGGGGTGGGTAATCCATTTG
TTTTTGCTGTGGAATTCGTACCGCCGA
CCCAACGTGTCTATAGGTATTATTGATGGACACATAGTATGTACGGCAAAATGTGTGCCACGTGGCGTTGTACATTTCGTATGGTGGGTTAACGATTCTCCCATCAACCACG
CGTGGTTGATGGGAGAATCGTTAACCCACCATACGAAATGTACAACGCCACGTGGCACACATTTTGCCGTACATACTATGTGTCCATCAATAATACCTATAGACACGTTGGG
CAAACAGTGCGCTTCAAGTCTCAATGAAACAGTAAAAGATTCTCCCCCCGCGATTTATGAAGATAGGTTAGAACACACGCCGGTACAATTACCCCGCGACG

CGTCGCGGGGTAATTGTACCGGCGTGTGTTCTAACCTATCTTCATAAATCGCGGGGGGAGAATCTTTTACTGTTTCATTGAGACTTGAAGCGCACTGTTTG

TTGTGTATGCTTCTTCCCGGGCGCGTAGTTCTACGGTAAACGAATCCTGGACCTCCTGAACGTAAGCTGGAAGCCCCGTCGGTTGCTTTGGTG

CACCAAAGCAACCGACGGGGCTTCCAGCTTACGTTCAGGAGGTCCAGGATTCGTTTACCGTAGAACTACGCGCCCGGGAAGAAGCATACACAA

TCACGGTAGTCCTGCCCCCCGGTCCGGGGTTGCTGTGCAGATTCAATCGAGCCATACACCACCGGGGTCGCCGATCGAACAGCAGGTT

AACCTGCTGTTCGATCGGCGACCCCGGTGGTGTATGGCTCGATTGAATCTGCACAGCAACCCCGGACCGGGGGGCAGGACTACCGTGA

TGTTTGTTACAAACGGTCCTGTTGAATCATGCAAAAGACAACGCAGGGATGTTTTTAATCCCGCCTCATCACGCCCGTAAATA

TATTTACGGGCGTGATGAGGCGGGATTAAAAACATCCCTGCGTTGTCTTTTGCATGATTCAACAGGACCGTTTGTAACAAACA

TGCACAGCAACCCCGGACCGGGGGGCAGGACTACCGTGATCGTCCAGTATCTGTGGGAATTAATCAAGACCCACGAACCATGGACAGACTGCCTTTTCGAGCCACGGATAGAGGAACAGAGGG

CCCTCTGTTCCTCTATCCGTGGCTCGAAAAGGCAGTCTGTCCATGGTTCGTGGGTCTTGATTAATTCCCACAGATACTGGACGATCACGGTAGTCCTGCCCCCCGGTCCGGGGTTGCTGTGCA

CAGGGATGTTTTTAATCCCGCCTCATCACGCCCGTAAATACCTATATAGTTTAATATCAACATTTTTGTAGGCTCTACAATTTCGGGTTGATACAGTTCCGCAAGTTGATCATCAAGCCATCCGAGTAAAGGTTGCATGTAACACGTGAAT

TGGGAATTAATCAAGACCCACGAACCATGGACAGACTGCCTTTTCGAGCCACGGATAGAGGAACAGAGGGAAACGCGAGATTCCCGTGTTACATGCAACCTTTACTCGGATGGCTTGATGATCAACTTGCGGAACTGTATCAACCCGAAAT

GGTACACAGACCGCTCCTTTGACATAAGTTTTCCTCGAATGGTTTGACGGCGGCATGGCGTTCTGGAGCGTACACGCATCGAAGGCCCGCTCCGAGATACGGATTTATATCGCCTAG

CTAGGCGATATAAATCCGTATCTCGGAGCGGGCCTTCGATGCGTGTACGCTCCAGAACGCCATGCCGCCGTCAAACCATTCGAGGAAAACTTATGTCAAAGGAGCGGTCTGTGTACC

ATGTCGGACAATAACCTCCGAGACCTAAATGATAACCCATTCCGGATAACGACAGGCAGTTATCCGCCACTGTCTGACCCAAGTTAAACGGAAGGGTGACAGGGGTCGTCTTAATAATTGGCACGATTAAGCC

GGCTTAATCGTGCCAATTATTAAGACGACCCCTGTCACCCTTCCGTTTAACTTGGGTCAGACAGTGGCGGATAACTGCCTGTCGTTATCCGGAATGGGTTATCATTTAGGTCTCGGAGGTTATTGTCCGACAT

CGCAACCCAGAACAACAAACAGAGACTGTGGTGCCATCCATTGGAACATCGGACGTTTACTGTAAAATGTGTGACCTTAACTTTGAAGGAGAATTGCTTTTGTATTCCAAAAGCAA

ATTGGCGAGAACAGGGGTCCGCATCTGGTCCTTGAGCCATCGACGTACATCAAAACTTCATTAAGTAGCAATAAACGTACACGCCCTAATGACTGGTAGACCGATACCATATCCGGCCCATATGACATTGGCTTTAT

ATAAAGCCAATGTCATATGGGCCGGATATGGTATCGGTCTACCAGTCATTAGGGCGTGTACGTTTATTGCTACTTAATGAAGTTTTGATGTACGTCGATGGCTCAAGGACCAGATGCGGACCCCTGTTCTCGCCAAT

ACTATGCTAGAAATACCTAGCCTTTCCCCTGCTCGTATAGAACCCTCACTCGCTAAAATCGCGTTAATTAACTCTCTATTAAAAACACGACTGTCCATGGTTTTTAC

GTAAAAACCATGGACAGTCGTGTTTTTAATAGAGAGTTAATTAACGCGATTTTAGCGAGTGAGGGTTCTATACGAGCAGGGGAAAGGCTAGGTATTTCTAGCATAGT

CCCGGAAACCCGTACGGTCCCCCGAAGTGTCCCGGAGGGCAACCATAACCCCCTGTATTAAATGGGAAGGCGGGCGGGTG

CACCCGCCCGCCTTCCCATTTAATACAGGGGGTTATGGTTGCCCTCCGGGACACTTCGGGGGACCGTACGGGTTTCCGGG

CCAACGCGGGTTTTAGGCACCCCATTCCTCGACGCATTCTAGCCAGGACGATTTTGCCTAATCTAAAATGCATGGGGAACATAGCGCAGTATATCGTGGGAAAAAAGGCGCTGAAATCAAACTTTGCCATCCAACGATCTTGTAGTTGTAC

TTCAAACAGCCTGTACAACTACAAGATCGTTGGATGGCAAAGTTTGATTTCAGCGCCTTTTTTCCCACGATATACTGCGCTATGTTCCCCATGCATTTTAGATTAGGCAAAATCGTCCTGGCTAGAATGCGTCGAGGAATGGGGTGCCTAA

CTAATCTAAAATGCATGGGGAACATAGCGCAGTATATCGTGGGAAAAAAGGCGCTGAAATCAAACTTTGCCATCCAACGATCTTGTAGTTGTACAGGCTGTTTGAAGACGGTAGATATCCGGCCCGCGTCCCAATTGACAGTTGCATCTGC

ATCTAGCCCCCTGGGGACAGCATCCCCCCTTACCTCCGCGGGTGCAGGATCTTATTGGGCAAATCCCTCAAGATACTGGACATGCAGATGCAACTGTCAATTGGGACGCGGGCCGGATATCTACCGTCTTCAAACAGCCTGTACAACTACA

CTCAACCTTTAGCGGCCGCCTTGGCTAATTGGAAAGATTGCGCGCGGATGGTTGACTGTTCATCATCGGAACATCGCACAAGTGGGATGATTACCTGCGCGGAACGTGCATTAAAAGAGGATATAGAGTTTGAAGATATATTAATAGACAA

TAAGACGATTTTTTAAGTTTGTCTATTAATATATCTTCAAACTCTATATCCTCTTTTAATGCACGTTCCGCGCAGGTAATCATCCCACTTGTGCGATGTTCCGATGATGAACAGTCAACCATCCGCGCGCAATCTTTCCAATTAGCCAAGG

GGTCATTTCATTATCCACACCTACCGCCGAAGAGGCCTCATAAGTGACGTTGTTTTCTATTGCACGGGGAACATTTATTAAGTGGGAGGCAAATAACAATTCTGCTAAAAAACGATTTGTTATTTTCAGAA

TTCTGAAAATAACAAATCGTTTTTTAGCAGAATTGTTATTTGCCTCCCACTTAATAAATGTTCCCCGTGCAATAGAAAACAACGTCACTTATGAGGCCTCTTCGGCGGTAGGTGTGGATAATGAAATGACC

TTCACAATCTTCATTTGATTCCCCAACACCCAACGCGAGATCGTATATGGGCCCGCCGGGGTGGAATGTGGCGTTTATAACACCCGCG

CGCGGGTGTTATAAACGCCACATTCCACCCCGGCGGGCCCATATACGATCTCGCGTTGGGTGTTGGGGAATCAAATGAAGATTGTGAA

CTTCATTTGATTCCCCAACACCCAACGCGAGATCGTATATGGGCCCGCCGGGGTGGAATGTGGCGTTTATAACACCCGCGTTGGGTAATTTAGACTCCACCCCATTAACGTTGGTTATC

GATAACCAACGTTAATGGGGTGGAGTCTAAATTACCCAACGCGGGTGTTATAAACGCCACATTCCACCCCGGCGGGCCCATATACGATCTCGCGTTGGGTGTTGGGGAATCAAATGAAG

AGAGATAGATGGGACGTGGTTGCAAAACGTAGACGCGAAGCCGGAATTATGGGTCACGTTTATCTTCAGGCTAGCACCGGATATGGACTTGCTCGGATAACCAACGTTAATGGGGTGGAGTCTAAATTACCCAACGCGGGTGTTATAAACG

TAGATGGGACGTGGTTGCAAAACGTAGACGCGAAGCCGGAATTATGGGTCACGTTTATCTTCAGGCTAGCACCGGATATGGACTTG

CAAGTCCATATCCGGTGCTAGCCTGAAGATAAACGTGACCCATAATTCCGGCTTCGCGTCTACGTTTTGCAACCACGTCCCATCTA

GGACTAAATGAGTTGAAGTAAAAGCCGTAGTCCATAATTCCGGTAAGTTTTCCGTTTTTTCAAGAAGCGCCTTTGCTTGGGTTTCTATGTCCGCGGCGGAC

GTCCGCCGCGGACATAGAAACCCAAGCAAAGGCGCTTCTTGAAAAAAAGGAAAACTTACCGGAATTATGGACTACGGCTTTTACTTCAACTCATTTAGTCC

TGTCCTAGAGGAGGTTTTATCTGCCCCGTAACAGGTCCCTCGTCGGGAAATCGAGAAACCACCCTATCCGACCAAGTTCGCGGTATAATTGTCAGTGGCGGGGC

CCCCCGCCACTGACAATTATACCGCGAACTTGGTCGGATAGGGTGGTTTCTCGATTTCCCGACGAGGGACCTGTTACGGGGCAGATAAAACCTCCTCTAGGACA

AATGTTTGATCACACACAATCGGATGTTGCTTATACAGATCGAGCAACTCTTAACCCATGGGCATCACAAAAACATTCATACGGTGACAGGCTATACAACGGAACATACAACCTTACAGGCTTCTCCTATCTAC

GTAGATAGGAGAAGCCTGTAAGGTTGTATGTTCCGTTGTATAGCCTGTCACCGTATGAATGTTTTTGTGATGCCCATGGGTTAAGAGTTGCTCGATCTGTATAAGCAACATCCGATTGTGTGTGATCAAACATT

TTTTTGAACATGTTTTTGATCGCCGTTATACTCCGGTATACTGGAGTCTTCAGGAGCAAAATTCGGAAACAAAAGCAATACCGGCATCTTATCTGACATACGGACAC

GTGTCCGTATGTCAGATAAGATGCCGGTATTGCTTTTGTTTCCGAATTTTGCTCCTGAAGACTCCAGTATACCGGAGTATAACGGCGATCAAAAACATGTTCAAAAA

AGAAGCCGTACGGCTTGCACATCAGGTTTTTGAAACCTGGCGTCAAACATTACAGTCATCGTTACTTGAGTTTCTGCGTGGACTAACCGGTTGTCTCTAT

ATAGAGACAACCGGTTATTCCACGCAGAAACTCAAGTAACGATGACTGTAATGTTTGACGCCAGGTTTCAAAAACCTGATGTGCAAGCCGTACGGCTTCT

CCCACGCCAATGGCATGCGCCAACAACTTTCCCGGGATGACCCGTGTTATAACTGCCGCGAATCGCATTCGGTATGCCTCTAACAGCGAGAGCGTGTCTGGTGGAGCACCGTTAATGTAATATGACGACAGCGCTATATCCACCAGTGAAG

GTCGGAAACCCTCAAGAATGTAATTCGTGTGCAATAACCTCAGCATCGCGGTTGTTTCTCGTGGGACTACAAGCAAGTGTTATCACGTCCGGGTTAATTTTACAATATCACGTCTGCGAAGCTGCCGTCAATGCA

TGCATTGACGGCAGCTTCGCAGACGTGATATTGTAAAATTAACCCGGACGTGATAACACTTGCTTGTAGTCCCACGAGAAACAACCGCGATGCTGAGGTTATTGCACACGAATTACATTCTTGAGGGTTTCCGAC

GTGTCTGAACATGGTGTCATAGAGACGGTCGCACTGCCCCATCCGGACAATTTAAAAGAATGTTTGTATTGCGGAAGTGTTTTTCTTAGGTA

TACCTAAGAAAAACACTTCCGCAATACAAACATTCTTTTAAATTGTCCGGATGGGGCAGTGCGACCGTCTCTATGACACCATGTTCAGACAC

TGACCGGTTTTCGACCGTTTATGTACTGAGGGCTTTGATATATAGCATTCAAAAGCCACCAACAGTAAACAACGGCCGTGAGAATATGACGCCCTAGCAATCCTGCTTCATCAATAACAATAACGTTGCTCCGGGTAAAAGCCGGAAGGGA

GGGTTTAAATAAAGTATGGAAGGTTAGCGAAGATAAACTCGCCAAGGTGTTATTATCCACAGCCGTGAACAATATGCTTTTAAGAGATAGATGGGA

TCCCATCTATCTCTTAAAAGCATATTGTTCACGGCTGTGGATAATAACACCTTGGCGAGTTTATCTTCGCTAACCTTCCATACTTTATTTAAACCC

TGGCGAGTTTATCTTCGCTAACCTTCCATACTTTATTTAAACCCGCGTAGTCTTTAACCAGCGACAATAACCGCGCTTTACTTTCCATCGATAAAACCCGGAATGGTTCAATTGAAGATTCCGGGGTACAGTCATAATTGACCACAGATCG

GTGGTCAATTATGACTGTACCCCGGAATCTTCAATTGAACCATTCCGGGTTTTATCGATGGAAAGTAAAGCGCGGTTATTGTCGCTGGTTAAAGACTACGCGGGTTTAAATAAAGTATGGAAGGTTAGCGAAGATAAACTCGCCAAGATCG

CCCAGCATGCGGCACAAGAACTAAAAACTCTGCTACCCCAGATGACCCCGGCAAACATGTCCTCCGGTGCATGGAATATATTGAAAGAGCTTGTTAATGCCGTTCAGGACAACACTTCCTAAATATACCTAGTATTTACGTATGTACCAGT

CAATGTGTATCATCTTTTTACTGGTACATACGTAAATACTAGGTATATTTAGGAAGTGTTGTCCTGAACGGCATTAACAAGCTCTTTCAATATATTCCATGCACCGGAGGACATGTTTGCCGGGGTCATCTGGGGTAGCAGAGTTTTTAGT

CCAGGTATACGACCGCCCCCCGGTAAGGAAATGGCCAACAAAGTAAGATATTCCAATTACTCCTTTTTTGTACAGGCGGTAGTTAGGGCTGCATTACTATCGATCAGCACGTCTCGT

ACGAGACGTGCTGATCGATAGTAATGCAGCCCTAACTACCGCCTGTACAAAAAAGGAGTAATTGGAATATCTTACTTTGTTGGCCATTTCCTTACCGGGGGGCGGTCGTATACCTGG

ATCGCAAAATAAATGTTTATATACGGCCTCGGAACCTGCTGCTTTTACAATGGTTATGAATCGACCCTCATCATTAAATGGAAATTTAAGTTTAATGGGGT

ACCCCATTAAACTTAAATTTCCATTTAATGATGAGGGTCGATTCATAACCATTGTAAAAGCAGCAGGTTCCGAGTCCGTATATAAACATTTATTTTGCGAT

CCTTTTAAACTGCCGCAAAGCGATCCAATAGTGCCCTATGGTTAATAAACAACATAACATGCATTCGGTATTCGTGCGACCACGGCACTTTGCGTTTTTTAAGGTAGT

ACTACCTTAAAAAACGCAAAGTGCCGTGGTCGCACGAATACCGAATGCATGTTATGTTGTTTATTAACCATAGGGCACTATTGGATCGCTTTGCGGCAGTTTAAAAGG

CAGGTTCCGAGGCCGTATATAAACATTTATTTTGCGATCTCCTATGCGCTCTCTCGGAATTACAGACAAACCCTAAAATTTTATTTGCCCATCC

GGATGGGCAAATAAAATTTTAGGGTTTGTCTGTAATTCCGAGAGAGCGCATAGGAGATCGCAAAATAAATGTTTATATACGGCCTCGGAACCTG

CATCGTACGCCTGATATGCCATCATAATAAGGCCATGGTAAAGCGCTCCGTCATATATTCTCATATTTCTGGTTGTTGCCGTGGCGGCTCCCGCATCGGGGG

CCCCCGATGCGGGAGCCGCCACGGCAACAACCAGAAATATGAGAATATATGACGGAGCGCTTTACCATGGCCTTATTATGATGGCATATCAGGCGTACGATG

TGGGGTAGTTGGGGCTTCTTCATCAGCGGGAATTTCCGGAACGATAACTGCCTGTAACGCAGGGTATAGGCGATCATAACGCACGCCCATTGTAC

GTACAATGGGCGTGCGTTATGATCGCCTATACCCTGCGTTACAGGCAGTTATCGTTCCGGAAATTCCCGCTGATGAAGAAGCCCCAACTACCCCA

TTATTAATTGGTGAAAGCAGCGATAGCGGTGGGGCTTTCTCCAATTAATAAATTCCAACCCGAGGGACATCTAAATCGTGTTGCACGCGCGGCCCTACTTTCGGACCTCAAACGTAGAGTCTGTGCGGATATGTTTTTTATGACCCGACAC

CATCACCGATGGTTGGGTGCACGAAACCATATCCGACAGATACGCAGAGATCAGCCTAGGTTCCCTGGCGTGTCGGGTCATAAAAAACATATCCGCACAGACTCTACGTTTGAGGTCCGAAAGTAGGGCCGCGCGTGCAACACGATTTAGA

TACATGGTAGGCAACGGGTTGGCGTATAGTTGCGTGGTGGTTGGCTCCCAGAAAAGGAGGGATTGGTGGTACCATTTTTGCCAAAACCCGCCTAACATTTGGCATCATTGCGTGGAATGACAAATGCTAG

CTAGCATTTGTCATTCCACGCAATGATGCCAAATGTTAGGCGGGTTTTGGCAAAAATGGTACCACCAATCCCTCCTTTTCTGGGAGCCAACCACCACGCAACTATACGCCAACCCGTTGCCTACCATGTA

ATATTTTAAGGTATGGAGTTCGGAAACGCGATCGAACAGGAATCGGAACGTTATCTTTATTTGGAATGCAAGCTCGATACAATTTGCGAAATGAATTTCCTCTTTTAACTACAAAGCGTGTTTTTTGG

CCAAAAAACACGCTTTGTAGTTAAAAGAGGAAATTCATTTCGCAAATTGTATCGAGCTTGCATTCCAAATAAAGATAACGTTCCGATTCCTGTTCGATCGCGTTTCCGAACTCCATACCTTAAAATAT

GTACCGACGGCTCATAGCCACGGCCGCGGCCGCGTGGGGGAGAGCCCAGAGGGCCTCCCCCGTGGCCATGGCTTCGCCTACATGCGGAACGGGAGACGCTACGCTCCCCGTAACGGCGGTACCCGCCC

GGGCGGGTACCGCCGTTACGGGGAGCGTAGCGTCTCCCGTTCCGCATGTAGGCGAAGCCATGGCCACGGGGGAGGCCCTCTGGGCTCTCCCCCACGCGGCCGCGGCCGTGGCTATG
AGCCGTCGGTAC
CACGTAGAGCGGCAAGAATGTGATCGCTAATTTCTGCGGATTTTTCATATCGTTCCCGTAGAGGGTTTAGATTCATTTTTAAGAACCTAGAGGAGACCGTACGCGACATGGCAACATACACACT
ATTTAGGCGCAGTTTATCCGCCGTAAA
ATCTGGACCCGTCATTCCACCTCAGTGGTTTTACCACCATAACACTACAGACACATACGGTGATGCGGCGGCAATGGCGTTTGGAAAAACCAACGAACCGGCGGCACGAGCGATAG
TTGAAGCATTGTTTATA
TATAAACAATGCTTCAACTATCGCTCGTGCCGCCGGTTCGTTGGTTTTTCCAAACGCCATTGCCGCCGCATCACCGTATGTGTCTGTAGTGTTATGGTGGTAAAACCACTGAGGTGGAA
TGACGGGTCCAGAT
GGGTTAATTTGGATGCTAACTCTTCCGGATGCGGCTCCTGAGGCTGTGTGGACGTCTTAAAGAACAGTGCGTTGGTAGAACCGGGTGTTGTGTAGGTTA
TAACCTACACAACACCCGGTTCTACCAACGCACTGTTCTTTAAGACGTCCACACAGCCTCAGGAGCCGCATCCGGAAGAGTTAGCATCCAAATTAACCC
ATAAAATTCTAACAACTCTGGAGTCTCCAAATGATGTAACCTACACAACACCCGGTTCTACCAACGCACTGTTCTTTAAGACGTCCACACAGCCTCAGGAGCCGCAT
ATGCGGCTCCTGAGGCTGTGTGGACGTCTTAAAGAACAGTGCGTTGGTAGAACCGGGTGTTGTGTAGGTTACATCATTTGGAGACTCCAGAGTTGTTAGAATTTTAT
CCGCACAGTGGGGTGGGATAGTCGGCCAGTTATATTCGTAAATGGCAGCCGCTTGTGTATCGACGCGCAATGGGACTCCCACCTCGTGTGCGCCGACCACA
TGTGGTCGGCGCACACGAGGTGGGAGTCCCATTGCGCGTCGATACACAAGCGGCTGCCATTTACGAATATAACTGGCCGACTATCCCACCCCACTGTGCGG
GTGTATTAAAGCATTGTATAAAAACACGCATGCGGGCTTGCTGTTCTCATTTCTAGGTTTTGTCTTAAATACACCCGCCATGAGCATCTCTGGACCCCCAACGACGTTTATTTTATATAGGTTAC
ATGGGGTCAGGCGGGTTCTTCACTGG
CAGGGCGTGGCGGCAAAACACCCTCGCCCAGGTGGGTACGTCGCCGGCCTCCGGCCCGGCGGCCCCCGGTCTCCGTCCCTCGGGAAGGAAGACGGG
CCCGTCTTCCTTCCCGAGGGACGGAGACCGGGGGCCGCCGGGCCGGAGGCCGGCGACGTACCCACCTGGGCGAGGGTGTTTTGCCGCCACGCCCTG
CGTGGCCATGGCTTCGCCTACATGCGGAACGGGAGACGCTACGCTCCCCGTAACGGCGGTACCCGCCCGTCCCGGTGGCAACAGCTTTTGGTAGAACTGGTTCA
TGAACCAGTTCTACCAAAAGCTGTTGCCACCGGGACGGGCGGGTACCGCCGTTACGGGGAGCGTAGCGTCTCCCGTTCCGCATGTAGGCGAAGCCATGGCCACG

TGAGCGGCCCCGACACCGGGGGAATTATCGGGGTTCTGGATCGCGGCAGGGAAAATGATTTCTGTCTCTGGCGCCCCGGTTCCCCCGCAAGACGTTTGGTCTTACGAATCCTCGGATCGGGACCGCTGATGGATCGA

TCGATCCATCAGCGGTCCCGATCCGAGGATTCGTAAGACCAAACGTCTTGCGGGGGAACCGGGGCGCCAGAGACAGAAATCATTTTCCCTGCCGCGATCCAGAACCCCGATAATTCCCCCGGTGTCGGGGCCGCTCA

TGAGCGGCCCCGACACCGGGGGAATTATCGGGGTTCTGGATCGCGGCAGGGAAAATGATTTCTGTCTCTGGCGCCCCGGTTCCCCCGCAAGACGTTTGGTCTTACGAATCCTCGGATCGGGACCGCTGATGGATCGA

CCCAAACGGGGGATTTCGGCGTATTCCCCGGGGGGCCCTGCATACCCCGGTCCCGTCGGACCAGGCTCGCAAGGCGTACTGTACCCCCGAAACCATCGCCCGTCTGGTCGACGACCCATTGTTTCCCACGGCCTGGCGCCCTGCGCTAAGC

CCCAGCGGAGTGGAGGCGCTGCGACGGAGGTGCGCCTGGATGCGGCAGATCCCAGACCCGGAGGATGTGAGGCTTCTGATCATCTACGATCCGTTGCCCGGAGAGGACATCAAC

GTTGATGTCCTCTCCGGGCAACGGATCGTAGATGATCAGAAGCCTCACATCCTCCGGGTCTGGGATCTGCCGCATCCAGGCGCACCTCCGTCGCAGCGCCTCCACTCCGCTGGG

TCCAATGTTTGATATCGGCCGTTATATTTTACATACAAGTGACCAACGCTTTGATCTTCAAGGGCGACCACGTGAGCTTGCAAGTTAACAGCAGTCTCACGTCTATCCTTATTCCCATGCAAAATGATAA

TTATCATTTTGCATGGGAATAAGGATAGACGTGAGACTGCTGTTAACTTGCAAGCTCACGTGGTCGCCCTTGAAGATCAAAGCGTTGGTCACTTGTATGTAAAATATAACGGCCGATATCAAACATTGGA

TGGTTACATGAGGACGTTGTTACGACAGAAACTAAGTCCGTTGTTAAGGAGGGGATAGAAAATCACGTATATCCAACGGATATGTCCACGTTACCCGAAAAGTCCC

GGGACTTTTCGGGTAACGTGGACATATCCGTTGGATATACGTGATTTTCTATCCCCTCCTTAACAACGGACTTAGTTTCTGTCGTAACAACGTCCTCATGTAACCA

GTTGTGCAATGGCGGCCTCCAACATCACATCGGATTCTGGTGTCGCATTTTGTATGCCCCTTCTTGTTTTTGTATTTGGGCGATAAATTGGATGTTTTTAATTCTACGTCGCTTAACGCTTA

TAAGCGTTAAGCGACGTAGAATTAAAAAACATCCAATTTATCGCCCAAATACAAAAACAAGAAGGGGCATACAAAATGCGACACCAGAATCCGATGTGATGTTGGAGGCCGCCATTGCACAAC

CCATCGAGACAGACAAACTCAGTCCATCTCGGGTAAAAAGCCGGGCGATGAGCAAGCGGGTCATGCGCATGCATCGGGGGACGGAGTAGTTC

GAACTACTCCGTCCCCCGATGCATGCGCATGACCCGCTTGCTCATCGCCCGGCTTTTTACCCGAGATGGACTGAGTTTGTCTGTCTCGATGG

GTTCGTAAACGATCATCCGGTGGACACACAGAAAGAGAGCGCGCGGCGGCCGACGGCTGAGGGTCGGGAGTCTGTGTGGCCGGGGTTGTTGGAGAAG

CTTCTCCAACAACCCCGGCCACACAGACTCCCGACCCTCAGCCGTCGGCCGCCGCGCGCTCTCTTTCTGTGTGTCCACCGGATGATCGTTTACGAAC

AAACACCGTGATCGATCCCTGTCAAATCGAAGACGGCGTCCTACAACGCCTCCTGCAATGACCACGGGAGAAAGAAATGATCAGACACATGACGAATCGTACAGGTTGCGATTTTCCAAGAGAGACGCCCGCCGAGAGCGTATTCGAAAAG

ACTCTTTTCGAATACGCTCTCGGCGGGCGTCTCTCTTGGAAAATCGCAACCTGTCCGGTTCGTCATGGGTCTGGTCATTTCTTTCTCCCGTGGTCAGTGCGGGAGGCGTTGTGGGACGCCGTCTTCGATTTGACAGGGATCGATCACGGTG

GTAGGTTCCGGTTCACCCGATAATCTTTGTGTCTCGGGGAAGCAAATTCGCTGAAGCAGACCACAGCCGTTAATAATAGCCCGGCTTAATGTTTCTCCAAACATATAAAGCTGCCACCCAGATGAATTTACTGGTACAGAGAGACCACTGG

CCAGTGGTCTCTCTGTACCAGTAAATTCATCTGGGTGGCAGCTTTATATGTTTGGAGAAACATTAAGCCGGGCTATTATTAACGGCTGTGGTCTGCTTCAGCGAATTTGCTTCCCCGAGACACAAAGATTATCGGGTGAACCGGAACCTAC

CACAGAAGGACTCGCCCGGGCCGTGTTTGCATCGTCGCGCACCTTTGGGCCAGCGAGTTTTGCTCGTATCGAGACTGCGTTTGCCAACCTGTACCCGGGCG

CGCCCGGGTACAGGTTGGCAAACGCAGTCTCGATACGAGCAAAACTCGCTGGCCCAAAGGTGCGCGACGATGCAAACACGGCCCGGGCGAGTCCTTCTGTG

TAAACGAACATGAAAAACACGCAGAACTTACAGCCGTAACGGTTGAGTTGGCGTTATTTGGAAAAACTCCCATACACTTTGATAGGGCG

CGCCCTATCAAAGTGTATGGGAGTTTTTCCAAATAACGCCAACTCAACCGTTACGGCTGTAAGTTCTGCGTGTTTTTCATGTTCGTTTA

TGGCAAAGTTTATGGGCGGTGTCGTTGGTTCCAACTGGCTTCTCAGTCCATTTAGGGGCTTTTATTGCTTTTCTGGGGTAGAAGGCGTTACTTTTGC

GCAAAAGTAACGCCTTCTACCCCAGAAAAGCAATAAAAGCCCCTAAATGGACTGAGAAGCCAGTTGGAACCAACGACACCGCCCATAAACTTTGCCA

TAATGTTTTTAAACCTGCCCCTCGGTGTATGTACGCCATCAGCGAGCTTAAATTCTGGTTAACGTCTAATTCCAC

GTGGAATTAGACGTTAACCAGAATTTAAGCTCGCTGATGGCGTACATACACCGAGGGGCAGGTTTAAAAACATTA

GTACGTTGGGGTCCTTCAGTGGAATTAGACGTTAACCAGAATTTAAGCTCGCTGATGGCGTACATACACCGAGGGGCAGGTTTAAAAACATTATAAGAATCAAGCATGGTGCTCGCAATTTCGTATGTTTGGTCAAACGTATCGTTCTTTG

CCCTGATGTACCGGATATCAAACGTCAATCCGCCGTTGAAGCGTTACGAACACTTCAAACCAAGACGGTAGTCAATCCCATGGGAGCAAAGAACGATACGTTTGACCAAACATACGAAATTGCGAGCACCATGCTTGATTCTTATAATGTT

ACAACAGAGTTTGCCAAAAAGCGTGGCTTCAAACCGGTTACCTCCCGCGCCTCGCATACGAATCTTGGTATTGCTTGTATTCTAAGATCTTCGATCACGTCGCTCACATCCAACCCC

GGGGTTGGATGTGAGCGACGTGATCGAAGATCTTAGAATACAAGCAATACCAAGATTCGTATGCGAGGCGCGGGAGGTAACCGGTTTGAAGCCACGCTTTTTGGCAAACTCTGTTGT

CAACTCGTTCCGAACTCTCTTAACGTTTACTTCCATAATGCACACCTAACCGTTGATGGTGATGCATTGCTCACACTACAAGAGTTAATGGGAGATATGGCTGAACGAACGACGGCCATTTTAGTATCAAGCGCCCCCGATGCGGGAGCCG

CTGCATGCAATCCTAGAGGTCGAGCATCTGGAATGTTATATATGGGTGACCGTGACGCCGACATAGAGGCTATAATGTTTGATCACACACAATCGGAT

ATCCGATTGTGTGTGATCAAACATTATAGCCTCTATGTCGGCGTCACGGTCACCCAGATATAACATTCCAGATGCTCGACCTCTAGGATTGCATGCAG

AACTGAGGAATGACACCCAAACAGACAAAATATAGAAGATCATAGTCACTGTTAACGTTGAACTGCGCAAGGCGGCGACTTTCTTCCAATGCCGCCCTTACACGCGGTTGGTGCATTAACATTCCAAGTCCCCGTTCATATTG

CAATATGAACGGGGACTTGGAATGTTAATGCACCAACCGCGTGTAAGGGCGGCATTGGAAGAAAGTCGCCGCCTTGCGCAGTTCAACGTTAACAGTGACTATGATCTTCTATATTTTGTCTGTTTGGGTGTCATTCCTCAGTT

GGGGCTCGGGGCAGCCGACTTTGTGGACGAGGCGGCACATAGCCACCGCGCAGCAAACCGATGGGGCCTGGGTGCCGCGCTTCGACCCGTCTTCCTTCCCGAGGGACGGAGACCGGGGGCCG

CGGCCCCCGGTCTCCGTCCCTCGGGAAGGAAGACGGGTCGAAGCGCGGCACCCAGGCCCCATCGGTTTGCTGCGCGGTGGCTATGTGCCGCCTCGTCCACAAAGTCGGCTGCCCCGAGCCCC

TGATGGTCACGGGCTCGGATACCCGCCTCTTGGCCTTGGAAACCACATGATCGTCTGCAACCCGGGCGTCCGCGACGGG

CCCGTCGCGGACGCCCGGGTTGCAGACGATCATGTGGTTTCCAAGGCCAAGAGGCGGGTATCCGAGCCCGTGACCATCA

TTGTCGAGGAGGCTTCTGCTCTCGACTGGCTGGGACTTGCGCTTGCGCGGAGTTCGTAAACGATCATCCGGTGGACACACAGACTCCCGACCCTCAGCCGTCGGCCGCCGCGCGCTCTCTTTCTGT

ACAGAAAGAGAGCGCGCGGCGGCCGACGGCTGAGGGTCGGGAGTCTGTGTGTCCACCGGATGATCGTTTACGAACTCCGCGCAAGCGCAAGTCCCAGCCAGTCGAGAGCAGAAGCCTCCTCGACAA

CACCACAGGGCCCGAGGTGATGGTCACGGGCTCGGATACCCGCCTCTTGGCCTTGGAAACCACA

TGTGGTTTCCAAGGCCAAGAGGCGGGTATCCGAGCCCGTGACCATCACCTCGGGCCCTGTGGTG

TTACGAACTCCGCGCAAGCGCAAGTCCCAGCCAGTCGAGAGCAGAAGCCTCCTCGACAAGATTAGGGAGACACCCGTCGCGGACGCCCGGGTTGCAGACGATCATGTGGTTTCCAAGGCCAAGAGGCGGGT

ACCCGCCTCTTGGCCTTGGAAACCACATGATCGTCTGCAACCCGGGCGTCCGCGACGGGTGTCTCCCTAATCTTGTCGAGGAGGCTTCTGCTCTCGACTGGCTGGGACTTGCGCTTGCGCGGAGTTCGTAA

CATCAAATGCACTTGGCCCCGCATGCCGAAGCCATGCTACAATTAGCGCTATCGGAACTGTCCCCGGGATCGTGGCCGCGGATAAACGGGGCGGTAAATTTTGAAAGTTTATAACCCATTAATACCATATATGGACATCCATAGGGGGGGT

GCTGTCATTGTCCTGTCTAAGAGAACGCATACAGGAAGCTACGGCGTTAGCGCTATGTCTGAGATCGTGTATAAATGCACAAAATTG

CAATTTTGTGCATTTATACACGATCTCAGACATAGCGCTAACGCCGTAGCTTCCTGTATGCGTTCTCTTAGACAGGACAATGACAGC

CAATGGATGCTGACGACACACCCCCCAACCTCCAAATATCTCCAACTGCAGGACCTTTGCGTTCCCACCACAATACCGACGGACATGAACCAAATGCAACCGCAGCCGATCAGCAAG

CTTGCTGATCGGCTGCGGTTGCATTTGGTTCATGTCCGTCGGTATTGTGGTGGGAACGCAAAGGTCCTGCAGTTGGAGATATTTGGAGGTTGGGGGGTGTGTCGTCAGCATCCATTG

CTTCCGTTCAGGACATTGTCACGCAGTTTGCATTGCTGTTAGGGCGTCTGGAAGAAGAAACTGGTACGTTGGACATTCAGGCGGTTGACTGGATGTACCAAGCTCGCAATATTATTGACTCCCATCCACTAAGTGTGCGT

ACGCACACTTAGTGGATGGGAGTCAATAATATTGCGAGCTTGGTACATCCAGTCAACCGCCTGAATGTCCAACGTACCAGTTTCTTCTTCCAGACGCCCTAACAGCAATGCAAACTGCGTGACAATGTCCTGAACGGAAG

CACTACCAGTACGTGCTGCGTTTTGTCTAGAATATACGGCGATAGCCATTTTTTTACCGTTCCAGACGAGGGTTTTATGTGCACACAAATTCCCGCTAGAGCGTTTTTCGATGATGTGTGGATGGGACGTGAAGAGTCGTATACAATTATA

CAGTTATAATTGTATACGACTCTTCACGTCCCATCCACACATCATCGAAAAACGCTCTAGCGGGAATTTGTGTGCACATAAAACCCTCGTCTGGAACGGTAAAAAAATGGCTATCGCCGTATATTCTAGACAAAACGCAGCACGTACTGGT

CTTAGCGTATTAAAGCAAGACAACTCTGCTTCCGCGGAAGCCCGTTGCGTTGCGCGTGTATTAATGGTTTTAATTGTACGTGATAATTCATCGAACTCTCGTCTGGATAAGTAACCTGCGGTTTGAGCTTCCCCGACTAC

GTAGTCGGGGAAGCTCAAACCGCAGGTTACTTATCCAGACGAGAGTTCGATGAATTATCACGTACAATTAAAACCATTAATACACGCGCAACGCAACGGGCTTCCGCGGAAGCAGAGTTGTCTTGCTTTAATACGCTAAG

CCGACAAAATGTTACATCCCTACCCATAGCCGCCGTGGTATCAATACATGACAGTGTTATGTTGCAATATGAACGGGGACTTGGAATGTTAATGCACCAACCGCGTGTAAGTGCGGCATTGGAAGAAAGTCGCCGCCTTGCGCAGTTCAAC

CCAGCAATGTTGAACGGTACCCCAAGGCCCATATCCCCCGATCTCTGGTATACTTGGCAGGATAATTCACCGTTTGCAACGTAAAACTGACATAACGTGTGACATGGAGGTA

TACCTCCATGTCACACGTTATGTCAGTTTTACGTTGCAAACGGTGAATTATCCTGCCAAGTATACCAGAGATCGGGGGATATGGGCCTTGGGGTACCGTTCAACATTGCTGG

ATGGCATCTTCCGACGGTGACAGACTTTGTCGCTCTAATGCAGTGCGTCGTAAAACAACGCCTAGTTATTCCGGACAATATCGAACCGCGCGGCGAAGTGTGGTCGTAGGACCCCCCGATGATTCAGACGACTCGTTGGG

CCCAACGAGTCGTCTGAATCATCGGGGGGTCCTACGACCACACTTCGCCGCGCGGTTCGATATTGTCCGGAATAACTAGGCGTTGTTTTACGACGCACTGCATTAGAGCGACAAAGTCTGTCACCGTCGGAAGATGCCAT

ATGCATGGCTACGCTTTTCCAATCCAAACCCAGAATTTCATTCCTGTATTGCATAAGATTTTCGGCTAGGTCCACGGAGGGAATGGGCTGTTCTCGGGGATAGATGGTCTCCAATCCCACAAAACGGAAGTTCATCTCTATAG

CTATAGAGATGAACTTCCGTTTTGTGGGATTGGAGACCATCTATCCCCGAGAACAGCCCATTCCCTCCGTGGACCTAGCCGAAAATCTTATGCAATACAGGAATGAAATTCTGGGTTTGGATTGGAAAAGCGTAGCCATGCAT

ATGTGATTTAGAGGTATTACTGTTAGGCGAGGTCGACCTCCCAACTGTTTGTGAATCCTACGCAACTATTGCACACGAATTAACCGGATATGAGGCTGTTCGCACAGCAGCCACAAATTTTATGATAGAGTTTGCCGATTGTTATAAGGAA

TTTCCTTATAACAATCGGCAAACTCTATCATAAAATTTGTGGCTGCTGTGCGAACAGCCTCATATCCGGTTAATTCGTGTGCAATAGTTGCGTAGGATTCACAAACAGTTGGGAGGTCGACCTCGCCTAACAGTAATACCTCTAAATCACA

ATAACAGCTCATCCTCGCGGTCCGTTTCATATGTTGAGTCATTTTCATAGACGTAGCCGTAGCCTTGTGATGGGTAATTTGTGCGGCGAGAATTTCTATGTGCAGGTTTTACTTTTCGTATGTATCCCCGTACCCGCTCGGG

CCCGAGCGGGTACGGGGATACATACGAAAAGTAAAACCTGCACATAGAAATTCTCGCCGCACAAATTACCCATCACAAGGCTACGGCTACGTCTATGAAAATGACTCAACATATGAAACGGACCGCGAGGATGAGCTGTTAT

AATACGCGGGTTTTTGTAAAGGGGTTGTTATCAACGACATACATTAGAGTGTTTAACAACCATAAAACTCCCTCATATAAAAACCGACGCGTTGAGTGTCAAATAGGACCTTTGGAAAAAATGCGTCG

CGACGCATTTTTTCCAAAGGTCCTATTTGACACTCAACGCGTCGGTTTTTATATGAGGGAGTTTTATGGTTGTTAAACACTCTAATGTATGTCGTTGATAACAACCCCTTTACAAAAAC

CCGCGTATT

GCCGTAAAAATTACATCATCCGGGTCTTCGGGTATGGAGGCCGTTACTTTGGACTCGGGGGCTTTGGTAAAGCAGAAGGCTTTATCGGCAGAAGT

ACTTCTGCCGATAAAGCCTTCTGCTTTACCAAAGCCCCCGAGTCCAAAGTAACGGCCTCCATACCCGAAGACCCGGATGATGTAATTTTTACGGC

CATGCGGTTCCCTTCCGGCTTTTACCCGGAGCAACGTTATTGTTATTGATGAAGCAGGATTGCTAGGGCGTCATATTCTCACGGCCGTTGTTTACTGTTGGTGGCTTTTGAATGCTATATATCAA
ATCCCTCAGTACATAAACGGTCGAAA

TGATCCGGTAAAGTCCAGTGAAGAACCCGCCTGACCCCATGTAACCTATATAAAATAAACGTCGTTGGGGGTCCAGAGATGCTCATGGCGGGTGTATTTAAGACAAAACCTAGAAATGAGAA
CAGCAAGCCCGCATGCGTGTTTTTATACA

CCTCCGACGCTTCGGATAATACCTGCACCATATGCATGAGCACCGTTTCCGATCTCGGAAAAACCATGCCGTGTTTGCACGACTTCTGCTTTGTTTGTATTCGGGCATGGACCTCCACC
AGCGTCCAGTG

CACTGGACGCTGGTGGAGGTCCATGCCCGAATACAAACAAAGCAGAAGTCGTGCAAACACGGCATGGTTTTTCCGAGATCGGAAACGGTGCTCATGCATATGGTGCAGGTATTATC
CGAAGCGTCGGAGG

CTTTTTTCTGTGTTACCGGGGATTAAACCGGGAAGCGCAAGGGGTGCTAAGCGCCGTTTATTTCATACCGGCAGAGACGTTAAACGATGCTTAACAATAGACCTGACATCTGAGTCTG
ATTCGGCATGTAAGGG

CCCTTACATGCCGAATCAGACTCAGATGTCAGGTCTATTGTTAAGCATCGTTTAACGTCTCTGCCGGTATGAAATAAACGGCGCTTAGCACCCCTTGCGCTTCCCGGTTTAATCCCCG
GTAACACAGAAAAAAG

CCCTGGGCCCCTTCAATCGTCGGCTAGAGAGCCCACAGCAGAGTCACCAAGTGACTCTCAACAGGATTCTATACAACCACCGACCCGAGACTCGAGCCCTGGTG

CACCAGGGCTCGAGTCTCGGGTCGGTGGTTGTATAGAATCCTGTTGAGAGTCACTTGGTGACTCTGCTGTGGGCTCTCTAGCCGACGATTGAAGGGGCCCAGGG

CCTGTACGGGAGACGCAGCGCCCGCCATCCAGCATATATGTTTAAAACATACAACATGCTTTCAAGACGTGGTGGTGGATGTGGATTGCGCGGAAAATACTAAAGAGGATCAGTTGGCCGAA
ATCAGTTACCGTTTTCAAGGTAAGAAGGA

CACATATGAAACTCAGCCCCTCTTGGTTGAGGAGTTACTGCGGGCGTAGGGTTTCTTGTTTTTTCATCCCCTTTCCAGGTGACCAAAAACGTGGCGTAGGTAGACGTACCATCGGAGCCGCGCA
TGTTCCAAATGTACACACCCAAGTATT

GAACCGTGTTCCGTCGCTCCCCTCCAACACCGTCTCCGCGGCCCCAAAACCGGGCGGCCACATTACTCTGGGAATCGGGGGG

CCCCCCGATTCCCAGAGTAATGTGGCCGCCCGGTTTTGGGGCCGCGGAGACGGTGTTGGAGGGGAGCGACGGAACACGGTTC

TACCCCGGTCCCGTCGGACCAGGCTCGCAAGGCGTACTGTACCCCCGAAACCATCGCCCGTCTGGTCGACGACCCATTGTTTCCCACGGCCTGGCGCCCTGCGCTAAGCTTTG

CAAAGCTTAGCGCAGGGCGCCAGGCCGTGGGAAACAATGGGTCGTCGACCAGACGGGCGATGGTTTCGGGGGTACAGTACGCCTTGCGAGCCTGGTCCGACGGGACCGGGGTA

GTCTCCGCGGCCCCAAAACCGGGCGGCCACATTACTCTGGGAATCGGGGGGAGGGCATTCCGAGCCTCGTCCGCCGACGCATACAGCGCCACCGACCGACCGGCCACG

CGTGGCCGGTCGGTCGGTGGCGCTGTATGCGTCGGCGGACGAGGCTCGGAATGCCCTCCCCCCGATTCCCAGAGTAATGTGGCCGCCCGGTTTTGGGGCCGCGGAGAC

TCGATCCATCAGCGGTCCCGATCCGGGGATTCGTAAGACCAAACGTCTTGCGGGGGAACCGGGGCGCCAGAGACAGAAATCATTTTCCCTGCCGCGATCCAGAACCCCGATAATTC
CCCCGGTGTCGGGGCCGCTCA

CTGGAGAACTACTCCGTCCCCCGATGCATGCGCATGACCCGCTTGCTCATCGCCCGGCTTTTTACCCGAGATGGACTGAGTTTGTCTGTCTCGATGGACCACCGACGGCAAACCTGGT
GAATTTCCTCTCGTCGTTT

AAACGACGAGAGGAAATTCACCAGGTTTGCCGTCGGTGGTCCATCGAGACAGACAAACTCAGTCCATCTCGGGTAAAAAGCCGGGCGATGAGCAAGCGGTCATGCGCATGCATC
GGGGGACGGAGTAGTTCTCCAG
GAGGGAGCGATCGGCTCCTGTTGGTTCTCGCGCGCCGGCCTCCGAGAATCGGGTGTGGAAGACCTCGGCCAGCGGGATTACAGGCGAGCCCATTAGATCC

GGATCTAATGGGCTCGCCTGTAATCCCGCTGGCCGAGGTCTTCCACACCCGATTCTCGGAGGCCGGCGCGCGAGAACCAACAGGAGCCGATCGCTCCCTC

GCGGCGCGGGCTCGTTACGAGGCCTCAACTGAACCCGTGCCGCTTTACGTGCCGGAGTTGGGAGATCCGGCTAGACAGTACCGCGCGCTGATTAACCTGATCTACTGT

ACAGTAGATCAGGTTAATCAGCGCGCGGTACTGTCTAGCCGGATCTCCCAACTCCGGCACGTAAAGCGGCACGGGTTCAGTTGAGGCCTCGTAACGAGCCCGCGCCGC

TTCGGGATTCCGACGCGGAATCATCGGACGGGGAAGACTTTATAGTGGAAGAAGAATCAGAGGAGAGCACCGATTCTTGTGAACCAGACGGGGT

ACCCCGTCTGGTTCACAAGAATCGGTGCTCTCCTCTGATTCTTCTTCCACTATAAAGTCTTCCCCGTCCGATGATTCCGCGTCGGAATCCCGAA

TTCCTCCAAAGCCTTATTTGCTTCTGTAAGTTTAGCCCCGGCAGCCTGTCTGATGTCTGCTCTAAGTTCACTTAACGTTACACTTTCGGTTATAAAC

GTTTATAACCGAAAGTGTAACGTTAAGTGAACTTAGAGCAGACATCAGACAGGCTGCCGGGGCTAAACTTACAGAAGCAAATAAGGCTTTGGAGGAA

CGATGAGGACTCTTTCCCCTCGTGTCCCGATGATCCATTTTACTCGCAAATTATTGCCGGTTATGCGCCCCAAGGCCCCCCAAACCTCGACACAACTGATTTTTACCCAACGGAGCCACTATTTAAGTCTCCC

GTTAGACTTAAATAGTGGCTCCGTTGGGTAAAAATCAGTTGTGTCGAGGTTTGGGGGGCCTTGGGGCGCATAACCGGCAATAATTTGCGAGTAAAATGGATCATCGGGACACGAGGGGAAAGAGTCCTCATCG

TTAAAGAAGGTGTCTTCATTACATTACCAAGCGAAGGTTTATTGAATCGGGCTATAGAGTATTTTACAACTTTCGATCATAAACGATTTATAG

TCCTTTATACTATCTACGGAAGTTGCGTACGTGTCCCCCGATGCCAACATATCCCCCGTTTCTCTTTTAAATGTTGTCCATGCATCGTCCCACGTAACCTCACCGGGTTCTATTCGTCG

CAAAGTGCAAAGGGCCACGGAGCGCAAACTTCCAACACAGATCCGATCAATAACGGTTCCTTTGATGGCGTCCTTGAGCCGCCTGGACAAGGGCGATTTACGGGAAAGAAAAACAATTC

GAATTGTTTTTCTTTCCCGTAAATCGCCCTTGTCCAGGCGGCTCAAGGACGCCATCAAAGGAACCGTTATTGATCGGATCTGTGTTGGAAGTTTGCGCTCCGTGGCCCTTTGCACTTTG

CCTGGAGTTAACAATGTTTACGGCCCGTTTTATTAGCTAAAGGACGTCCCGATTATTTTCCTGTATGCGTGTTTCAGCAGGGGAGTCATCAATACCTCCAGAAG

CTTCTGGAGGTATTGATGACTCCCCTGCTGAAACACGCATACAGGAAAATAATCGGGACGTCCTTTAGCTAATAAAACGGGCCGTAAACATTGTTAACTCCAGG

CGAGGAAAGTCCAAAACTATCATAAGGGAATATTGATCTGGCTTCAACTTCCTCAATGATGCAATTCACCGACGTGCCCGTCCTATATGTTCCGGGGGTTCCGGC

CTAACGTCATATATTTAATCATTTCCTGGGCTTCTCGAAATTTATCGGGATCAAACCCGCTATTTACCGACGGTTCAGTGTTTTGTGAGTCGCCAATTTCTTC

GAAGAAATTGGCGACTCACAAAACACTGAACCGTCGGTAAATAGCGGGTTTGATCCCGATAAATTTCGAGAAGCCCAGGAAATGATTAAATATATGACGTTAG

TGAGCAATTCCCTCGCCCGTCTCTATCTCCAAGAATTGGTCCGTGAAAACACTAATCATTCACCACAAAAACACCCGACTCGAAATACCAGATCCCGACGAAGCGTGCCAGTTGAGTTGCGTG

CACGCAACTCAACTGGCACGCTTCGTCGGGATCTGGTATTTCGAGTCGGGTGTTTTTGTGGTGAATGATTAGTGTTTTCACGGACCAATTCTTGGAGATAGAGACGGGCGAGGGAATTGCTCA

GTATCTCCTCGTCGTGGTGCCAGCTACAAAATCGCGAACGCGCCCTTTGGAGCGGACTATTTCCAATTAACCCAAGTGCTTTAGCGAGCACCATTTTGGATCAACGTGTTAAAGCTCGTATTCTCGGCGACGTTATCTCCGTTTCTAATTG

TCCCAGTTCTGGACAATTAGAAACGGAGATAACGTCGCCGAGAATACGAGCTTTAACACGTTGATCCAAAATGGTGCTCGCTAAAGCACTTGGGTTAATTGGAAATAGTCCGCTCCAAAGGGCGCGTTCGCGATTTTGTAGCTGGCACCAC

CCAGAGGGGCGGCGGTTGTCACAAGCCTAGTTGAGTGTATGCAAAATGTTGCTATTATGGATTATGATAGGACGCGTCTTTATTATAATTAT

ATAATTATAATAAAGACGCGTCCTATCATAATCCATAATAGCAACATTTTGCATACACTCAACTAGGCTTGTGACAACCGCCGCCCCTCTGG

CTTACTGCTGTCGTGGTCGCCATTGTTGCCGTTTTTCCCGAGGAACCTCCCAACTCAACTACACGAAACTACTGTCCGGAAGGG

CCCTTCCGGACAGTAGTTTCGTGTAGTTGAGTTGGGAGGTTCCTCGGGAAAAACGGCAACAATGGCGACCACGACAGCAGTAAG

CATTCCCTAAGGGCTGAGGAAGCATCGGCCATAATTGTGCGCAGGCTGGATATTCCCACTCTCATTTGCGTTCGGATTCCTTGTATTGTCCCAACAATATGACCAACCGCATCAGCAAGATCGTTTAAACGCCATACCCCAAATTGTTCAT

CCAAAGGTTGAACGCGATGAACAATTTGGGGTATGGCGTTTAAACGATCTTGCTGATGCGGTTGGTCATATTGTTGGGACAATACAAGGAATCCGAACGCAAATGAGAGTGGGAATATCCAGCCTGCGCACAATTATGGCCGATGCTTCCT

TGCGGTTTTCGTACACGCGACCGTTATTTCACAGGCGTTCATAATCAAGCTGCGGCGGATGGTGTCGGTTAATTGTCTCCGCCCAAGTTCGTCAATAGATGATACCATGAACAACGTATCAAATGGTACATAGTCGTCTTTGGTTTTCTCA

TAGCCAGCCAACACAGTGTTCAGACCGATTTACAGAAAATAGCGTTGATGCAAATGAAAATTTTCCGATTGGGCACGCGGGCTGTATTGAGAAAACCAAAGACGACTATGTACCATTTGATACGTTGTTCATGGTATCATCTATTGACGA

GTAAAGTGGCCAGGGCAGTGCCATGGAGACAACGCCAGGTGTCTGCGTTACCCAGTGGTTCAAGGGGCCAGGCGTCTAACGCAGCCGAGGAC

GTCCTCGGCTGCGTTAGACGCCTGGCCCCTTGAACCACTGGGTAACGCAGACACCTGGCGTTGTCTCCATGGCACTGCCCTGGCCACTTTAC

CATAAATTATCCTGAATGGTATATTCCAGCAGGGACCCAAAAAAACTTATAAATCGATGTGGAAATACATGACATTGTACCATCGCACGTAAACACTCCGAAAACCTTATGAGCCG

CGGCTCATAAGGTTTTCGGAGTGTTTACGTGCGATGGTACAATGTCATGTATTTCCACATCGATTTATAAGTTTTTTTGGGTCCCTGCTGGAATATACCATTCAGGATAATTTATG

CCATTTGATGATCTAACGCTTCCACCTCGGGTGTCGTGGTGTCGTACGGTGAGACTTTTTCAGAACGCGGCCCCTTCTTTTGAGTTCCCATGTCTCCCAACACCGGGGAGAGCAACGCCGCCGTCTATG

CATAGACGGCGGCGTTGCTCTCCCCGGTGTTGGGAGACATGGGACCTCAAAAGAAGGGGCCGCGGTCTGAAAAAGTCTCACCGTACGACACCACGACACCCGAGGTGGAAGCGTTAGATCATCAAATGG

AGTTGATACCAACCAAAACCTGGAGTTGATGGGGAACGTGTTCGAGTTCGACCGGCAAGTCGCCAATTAACGTTAAGCGACGTTGAAATTGACTGCATGTCGGATAAT

ATTATCCGACATGCAGTCAATTTCAACGTCGCTTAACGTTAATTGGCGACTTGCCGGTCGAACTCGAACACGTTCCCCATCAACTCCAGGTTTTGGTTGGTATCAACT

TACAAAATGACAGTGTGTTCCAGCCCGCGTGCCAGTTGTTAGTTTTAAGAAATATAATTGTATTAATAAGCATTATATATACATTTCTCAGAACCATAACAAACGGCAGATTAACCGTTTC

GAAACGGTTAATCTGCCGTTTGTTATGGTTCTGAGAAATGTATATATAATGCTTATTAATACAATTATATTTCTTAAAACTAACAACTGGCACGCGGGCTGGAACACACTGTCATTTTGTA

CTCCCGGTGTATCCATTAATTAAACGGGAGCTTCCGAAAGCAGTCAACCCATCCGAGTGAATCTTAAACCTGACCCGTTGGCGTCGTTTACACAAGTTATACCGCCACTGGCGTTGGAAACAACGTG

CACGTTGTTTCCAACGCCAGTGGCGGTATAACTTGTGTAAACGACGCCAACGGGTCAGGTTTAAGATTCACTCGGATGGGTTGACTGCTTTCGGAAGCTCCCGTTTAATTAATGGATACAACGGGAG

CTGCCAGCGCTCTTACTGCGCTTTTGCGTATTAGTGTATTATGGCGAAAGGAAGAGTGGCGGGATGGTTTAAATGGAACCGCAGCTGCAATTGTTGCGGCGGTTGAAC

GTTCAACCGCCGCAACAATTGCAGCTGCGGTTCCATTTAAACCATCCCGCCACTCTTCCTTTCGCCATAATACACTAATACGCAAAAGCGCAGTAAGAGCGCTGGCAG

TTTTCAGATAAAGCCTTTACCGCCGTCTCCAAAGCCTCGCCGGGCGTTGGAATAAGACATCCATCCCTCACGCGCTCGCTGGTTATGCGTTGGGAAATAGAACACCAGTCCCGGGTAGG

CCTACCCGGGACTGGTGTTCTATTTCCCAACGCATAACCAGCGAGCGCGTGAGGGATGGATGTCTTATTCCAACGCCCGGCGAGGCTTTGGAGACGGCGGTAAAGGCTTTATCTGAAAA

GTCTTATTCCAACGCCCGGCGAGGCTTTGGAGACGGCGGTAAAGGCTTTATCTGAAAAGACCGACAGCCTAACATCGCCGGTTTTACAAAGTACCGAAAGACACAGTGTTCTGCTTGGAT

ATCCAAGCAGAACACTGTGTCTTTCGGTACTTTGTAAAACCGGCGATGTTAGGCTGTCGGTCTTTTCAGATAAAGCCTTTACCGCCGTCTCCAAAGCCTCGCCGGGCGTTGGAATAAGAC

CTGGCGGCAAGAACCTGAGCATAATACTTTGTGGGTTTACCCCCATGTGCTAAAATACTCTGCGCCACGGCCCGGGAAAACCACATATGGGTGCTGGTCTCTAACACAACCCAACTGTG

CACAGTTGGGTTGTGTTAGAGACCAGCACCCATATGTGGTTTTCCCGGGCCGTGGCGCAGAGTATTTTAGCACATGGGGGTAAACCCACAAAGTATTATGCTCAGGTTCTTGCCGCCAG

GCTAGTATCAGAGCCGCCCGATCGGTTCGACATAGACGCGGTTCTCCAGATGCAGTGCATGTCGGACAATAACCTCCGAGACCTAAATGATA

TATCATTTAGGTCTCGGAGGTTATTGTCCGACATGCACTGCATCTGGAGAACCGCGTCTATGTCGAACCGATCGGGCGGCTCTGATACTAGC

TTGCTTTTGGAATACAAAAGCAATTCTCCATCAAAGTTAAGGTCACACATTTTACAGTAAACGTCCGATGTTCCAATGGATGGCACCACAGTCTCTGTTTGTTGTTCTGGGTTGCG

TATGACAACACACGAAAATAAAAACAGTTATCAATTTTGCTATGATCGTCTACCACGCGACCAGTTTCATGCTCGTGGGGGGCGGTATGA

TCATACCGCCCCCCACGAGCATGAAACTGGTCGCGTGGTAGACGATCATAGCAAAATTGATAACTGTTTTTATTTTCGTGTGTTGTCATA

ATAGTCACTGTTAACGTTGAACTGCGCAAGGCGGCGACTTTCTTCCAATGCCGCCCTTACACGCGGTTGGTGCATTAACATTCCAAGTCCCCGTTCATATTGCAACATAACACTGTCATGTATTGATACCACGGCGGCTATGGGTAGGGAT

GTCAGCTCTGTGGTCCGATTTAACGTATACAGTGGTGCTATATATCGTTCACATAAAAATTGAAAGTTATTATTACCGCTTTTAAACTTCCCATCAAACCCCGTCGCTCCGCGCAAGATTA

TAATCTTGCGCGGAGCGACGGGGTTTGATGGGAAGTTTAAAAGCGGTAATAATAACTTTCAATTTTTATGTGAACGATATATAGCACCACTGTATACGTTAAATCGGACCACAGAGCTGAC

CCGCCGTGGTATCAATACATGACAGTGTTATGTTGCAATATGAACGGGGACTTGGAATGTTAATGCACCAACCGCGTGTAAGGGCGGCATTGGAAGAAAGTCGCCGCCTTGCGCAGTTCAACGTTAACAGTGACTATGATCTTCTATATTT

CCCTGCGTCATCGAGCTTCACTGGTGGATATAGCGCTGTCGTCATATTACATTAACGGTGCTCCACCAGACACGCTCTCGCTGTTAGAGGCATACCGAATGCGATTCGCGGCAGTTATAACACGGGTCATCCCGGGAAAGTTGTTGGCGCA

ATCTGTATATACACGACCACAAGGTGGGGGGTATATACCCTACCTTCACGCTTCC

GGAAGCGTGAAGGTAGGGTATATACCCCCCACCTTGTGGTCGTGTATATACAGAT

ATGAAATTGTAGCCCGCACACCAGACCTGATTCAGACTATAGTTTCGGTGTTAACGGAAATGTCAATGGATGCGTTTTATAACAGCTCCTTGATGTATGCGGTTTTGGCGTATCTGTCATCTGTATATACACGACCACAAGGTGGGGGGTA

CAACCTTTTCTAAGGCTACGGGAACGGGGACCTTGGAAAGCTTAAGTATTTCCCCTCCTGAATTATAATAGTCAAATAAATATATAGAACGATTACCTAACCAGCATGGGAAGGAAGCGTGAAGGTAGGGTATATACCCCCCACCTTGTGG

TTGTATATCACGAATCTTCTGTTCAACTGAAGCTATGCGAGCTGCAGAGCGGAGCTGGTTATTTAAATCGGCGCAGGCCTCTTTTGCAGCAGTAAAG

CTTTACTGCTGCAAAAGAGGCCTGCGCCGATTTAAATAACCAGCTCCGCTCTGCAGCTCGCATAGCTTCAGTTGAACAGAAGATTCGTGATATACAA

CTGAGTTTAGAGAAGGTAGCTATCTGTTTTACGGCGGATAAACTGCGCCTAAATAGTGTGTATGTTGCCATGTCGCGTACGGTCTCCTCTAGGTTCTTAAAAATGAATCTAAACCCTCTACGGGAACGATATGAAAAATCCGCAGAAATTA

GCATACAAAATGCGACACCAGAATCCGATGTGATGTTGGAGGCCGCCATTGCACAACTAGCAACGATTCGCGAAGAATCCCCCCCACATTCCGTTGT

ACAACGGAATGTGGGGGGGATTCTTCGCGAATCGTTGCTAGTTGTGCAATGGCGGCCTCCAACATCACATCGGATTCTGGTGTCGCATTTTGTATGC

ATATAACCGCATTGGTTGACATGTAGGATCGATGGGGACATACAACCACTCTAACAGCAAATCAAATGGCGCTTCATGTATCTTATACTGAAGATGCATTGCCAAGCTAAACGTATCACCAACTGAAAATACATGCGAG

CTCGCATGTATTTTCAGTTGGTGATACGTTTAGCTTGGCAATGCATCTTCAGTATAAGATACATGAAGCGCCATTTGATTTGCTGTTAGAGTGGTTGTATGTCCCCATCGATCCTACATGTCAACCAATGCGGTTATAT

TCATATGTGGAATTACCACTCGCATGTATTTTCAGTTGGTGATACGTTTAGCTTGGCAATGCATCTTCAGTATAAGATACATGAAGCGCCATTTGATTTGCTGTTAGAGTGGTTGTATGTCC

GGACATACAACCACTCTAACAGCAAATCAAATGGCGCTTCATGTATCTTATACTGAAGATGCATTGCCAAGCTAAACGTATCACCAACTGAAAATACATGCGAGTGGTAATTCCACATATGA

TACAAACACAAGGAGGGGGGTCACCATTATTTCATCAGATCCCGTGGGTGTGGTTTCCTTTATTAAAGCCACGGTATCCCTCAGCTGGCGCATACCC

ATGCCATCGTTCCGCTTTAACAAAGTTTGAAGAAAATGCAGCTTGTGGGGTGGATTTACCCCTTAGAAGGCCTTCTTTAGGTGCTTGTGGTGGAA

TTCCACCACAAGCACCTAAAGAAGGCCTTCTAAGGGGTAAATCCACCCCACAAGCTGCATTTTCTTCAAACTTTGTTAAAGCGGAACGATGGCAT

CCCCACAAGCTGCATTTTCTTCAAACTTTGTTAAAGCGGAACGATGGCATGATTTCGCACGCTTTTTCGCAAGAGAGCATACGTGAATTTTCTTTTTGCATAGACGTCTTCGCTCTCTAA

CGGACCTTATCGGGGG

CCCCCGATAAGGTCCGTTAGAGAGCGAAGACGTCTATGCAAAAAGAAAATTCACGTATGCTCTCTTGCGAAAAAGCGTGCGAAATCATGCCATCGTTCCGCTTTAACAAAGTTTGAAGAAAATGCAGCTTGTGGGG

CATACAGAGCTTATGCAGAACAACGCGTCGCCACTCGTCGTTCGGGCAAGACCACCGGCGGTTCTTATTCCGCTGGTTGATGTCCCAAGGCCACGATCCCGGAGAAAGG

CCTTTCTCCGGGATCGTGGCCTTGGGACATCAACCAGCGGAATAAGAACCGCCGGTGGTCTTGCCCGAACGACGAGTGGCGACGCGTTGTTCTGCATAAGCTCTGTATG

AAAGGGGGCGGTTGTAAAGCTGCTTTCGAAAAGCTCTGCTAATATGGCATATCGACCATCGTAAATAGCTTTCATTAACAGAAACTGAGCATGGACC

GGTCCATGCTCAGTTTCTGTTAATGAAAGCTATTTACGATGGTCGATATGCCATATTAGCAGAGCTTTTCGAAAGCAGCTTTACAACCGCCCCCTTT

TCGGGAAATCGAGAAACCACCCTATCCGACCAAGTTCGCGGTATAATTGTCAGTGGCGGGGCCATGGTTCAATTAGCCATATACGCCACGGTTGTGC

GCACAACCGTGGCGTATATGGCTAATTGAACCATGGCCCCGCCACTGACAATTATACCGCGAACTTGGTCGGATAGGGTGGTTTCTCGATTTCCCGA

GCACAACCGTGGCGTATATGGCTAATTGAACCATGGCCCCGCCACTGACAATTATACCGCGAACTTGGTCGGATAGGGTGGTTTCTCGATTTCCCGA

TCGGGAAATCGAGAAACCACCCTATCCGACCAAGTTCGCGGTATAATTGTCAGTGGCGGGGCCATGGTTCAATTAGCCATATACGCCACGGTTGTGC

CCCATTCAACACTCATCACAGGTCTATCGGAGAGGGTTTTGTATACCCAACACCCTTTTATAACACCGGGTTGTGCCATTTAATACATGACTGTGTTATTGCTCCCATGGCCGTTGCCTTGCGCGTCAGAAATGTAACTGCCGTCGCCCGA

CCACTTGAAGAGGACTGAAAATACGTGTACGTAATGTCAGGGGGGAGTACTGCCCCCTCGTGATTTTCATCAAAAGCAAGGTGGGCCGCTCCTCGGGCGACGGCAGTTACATTTCTGACGCGCAAGGCAACGGCCATGGGAGCAATAACAC

CGTGCAATTCCTGCCGATGTAGCAGGACGTAGTCCGCCGAAGATGGGTAGGGGTTCGGTGGGATTTAAGCGACCCCCCGATGCTGTTATACGACGCAACGATTC

GAATCGTTGCGTCGTATAACAGCATCGGGGGGTCGCTTAAATCCCACCGAACCCCTACCCATCTTCGGCGGACTACGTCCTGCTACATCGGCAGGAATTGCACG

ACTCACACACAGAGGCTTGCCCTCGTGCAATTCCTGCCGATGTAGCAGGACGTAGTCCGCCGAAGATGGGTAGGGGTTCGGTGGGATTTAAGCGACCCCCCGATGCTGTTATACGACGCAACGATTCG

CGAATCGTTGCGTCGTATAACAGCATCGGGGGGTCGCTTAAATCCCACCGAACCCCTACCCATCTTCGGCGGACTACGTCCTGCTACATCGGCAGGAATTGCACGAGGGCAAGCCTCTGTGTGTGAGT

CTTTCAATATTCCACGAATGTTGGAAACTGTTGGTCTTCCTTGGGCTGTTAGGGTTGGGAGAGTGGGTCGGTCTGACGTGGTAAGTGCGGCTTCACCAGAGTCTTCTGTCAAGTTGCATGTTAACGTCGACGTTAT

ATAACGTCGACGTTAACATGCAACTTGACAGAAGACTCTGGTGAAGCCGCACTTACCACGTCAGACCGACCCACTCTCCCAACCCTAACAGCCCAAGGAAGACCAACAGTTTCCAACATTCGTGGAATATTGAAAG

AACCATGGTGTTATAAAGCGGTGAATTATCACTGGATTTACACACTTTTGCGTCCTCTTCGGGGTGCTGGTTAACCATAACGCTAAGCAAGTCCTTTGTC

GACAAAGGACTTGCTTAGCGTTATGGTTAACCAGCACCCCGAAGAGGACGCAAAAGTGTGTAAATCCAGTGATAATTCACCGCTTTATAACACCATGGTT

AAGACAATTTTTATAAATTCATTCCTTTTTAAATATAGGTTATATGGTACACAAATATCATATCCCGCGTCTTCTTGGCGTTTTGGA

TCCAAAACGCCAAGAAGACGCGGGATATGATATTTGTGTACCATATAACCTATATTTAAAAAGGAATGAATTTATAAAAATTGTCTT

GCTTGTATTAAATTTAAACCCTCATGCACCGTAATACGAATAACGGCTCCGGTTAACAAACGGTCTAATTCGGCGTTATTCCTTGGGGGATTACTATTCCAAGCCGCTTCGGCAGCCTTTTGTGCCTGCATGGCGGCTACGCGCCGGACCG

CAAGCTCGTGGTGCGGTCCTACGCCATCATATAACAAACGCGTCTTTTGTGAAGCGGTCCGGCGCGTAGCCGCCATGCAGGCACAAAAGGCTGCCGAAGCGGCTTGGAATAGTAATCCCCCAAGGAATAACGCCGAATTAGACCGTTTGTT

ATGCAAACAGAGGGTTGACCGGAACGGGATAAAAAAAAGTACCCGTTGCAATGGTTTCATCGTACGCCTGATATGCCATCATAATAAGGCCATGGTAAAGCGCTCCGTCATATATTCTCATATTTCTGGTTGTTGCCGTGGCGGCTCCCGC

ACTTCGGATCGTATCTCATATACACGCCGAATTAACTCTTCGGGTGGAATGTCGGTTTCTTCCGGGGGTTCCTGGGGAACTTCTGG

CCAGAAGTTCCCCAGGAACCCCCGGAAGAAACCGACATTCCACCCGAAGAGTTAATTCGGCGTGTATATGAGATACGATCCGAAGT

CTATAAATCGTTTATGATCGAAAGTTGTAAAATACTCTATAGCCCGAATCAATAAACCTTCGCTTGGTAATGTAATGAAGACACCTTCTTTAA

ACCAACACGGTTGCACTGGACCCTAGATGTAGTTACAGCGAGGTTGATCCTTGGCATGCGGTTCTAGAAACAACCTCGACTGGGTCTGG

CCAGACCCAGTCGAGGTTGTTTCTAGAACCGCATGCCAAGGATCAACCTCGCTGTAACTACATCTAGGGTCCAGTGCAACCGTGTTGGT

CGACGAATAGAAACCGGTGAGGTTACGTGGGACGATGCATGGACAACATTTAAAAGAGAAACGGGGGATATGTTGGCATCGGGGGACACGTACGCTACTTCCGTAGATAGTATAAAGGC

TGGATTTAATTAAACAGCATGAGTATATGGCCCGTACCCTCAGACCCGTAATCGAGGCCGCATTTAGAGGTCGTTTACTCGTTCGCTCATTGGATGGAGACCCGAAAGGCAATGCCCGGG

CCCGGGCATTGCCTTTCGGGTCTCCATCCAATGAGCGAACGAGTAAACGACCTCTAAATGCGGCCTCGATTACGGGTCTGAGGGTACGGGCCATATACTCATGCTGTTTAATTAAATCCA

TGTTTGATGAACCCGTAGACTGACTGGATAGAACGTGGCCATCTCGATCTGTTTGGGGATTCGCAGCTAAATGAGGTCCGGCAAACTGGTAAAATCGATTAAATGATGGACCCGGTCCCCCATCCT

AGGATGGGGGACCGGGTCCATCATTTAATCGATTTTACCAGTTTGCCGGACCTCATTTAGCTGCGAATCCCCAAACAGATCGAGATGGCCACGTTCTATCCAGTCAGTCTACGGGTTCATCAAACA

TGCTACGAGCGGCCAAAAATGAAATTTCCTCCAGATCCAAATCTTCAACCCGGCACGCATAAACGTAACCCAGGGGCCCCGTGGGCACTGTCACAGTCTTCTGAGTATTTTCCATTTTGG

CCAAAATGGAAAATACTCAGAAGACTGTGACAGTGCCCACGGGGCCCCTGGGTTACGTTTATGCGTGCCGGGTTGAAGATTTGGATCTGGAGGAAATTTCATTTTTGGCCGCTCGTAGCA

CATTGTGTATTTTACGGACAGCAAGTTGAGGGGCGGAACTTTCGTAACCAATTCCAACCTGTTTTGCGGCGGCGTTTTGTTGACCTGTTTAATGGGGGGTTTATATCAACACGCTCTATAACCGTAACATTATCTGAAGGTCCTGTATCCG

CTTCCACGCTTACGCGCGCTAAATCCCCATCAAAGGTACGCCCCGCGGGCGCGTCTTGTCCCAATGTCGGATTTGGGGCGGATACAGGACCTTCAGATAATGTTACGGTTATAGAGCGTGTTGATATAAACCCCCCATTAAACAGGTCAAC

ACATTATCTGAAGGTCCTGTATCCGCCCCAAATCCGACATTGGGACAAGACGCGCCCGCGGGGCGTACCTTTGATGGGGATTTAGCGCGCGTAAGCGTGGAAGTTATTCGGGATATACGAGTTAAAAATAGGG

CCCTATTTTTAACTCGTATATCCCGAATAACTTCCACGCTTACGCGCGCTAAATCCCCATCAAAGGTACGCCCCGCGGGCGCGTCTTGTCCCAATGTCGGATTTGGGGCGGATACAGGACCTTCAGATAATGT

ATTAAGCCAGTGTCATTGTGTATTTTACGGACAGCAAGTTGAGGGGCGGAACTTTCGTAACCAATTCCAACCTGTTTTG

CAAAACAGGTTGGAATTGGTTACGAAAGTTCCGCCCCTCAACTTGCTGTCCGTAAAATACACAATGACACTGGCTTAAT

CTCAACATATACACGAGGTTTGACAGCCAAAAATGCTCTATCGTACATTTTACAGCGAATACATACAGACACAACGGAAATACACGCAGTATCGGAGTATATTACCAACGATAGAAAAGGCCTTAAAGTTCCAGACCG

CGGTCTGGAACTTTAAGGCCTTTTCTATCGTTGGTAATATACTCCGATACTGCGTGTATTTCCGTTGTGTCTGTATGTATTCGCTGTAAAATGTACGATAGAGCATTTTTGGCTGTCAAACCTCGTGTATATGTTGAG

TGTTATTGCTAATGATACGTGGTAATCAATAACAAACCGTTACACTTTACCGCACGAGGCTCGATTAATTGCGGAGGACCTTAAACAAATTAATCCATGTAATTTTGT

ACAAAATTACATGGATTAATTTGTTTAAGGTCCTCCGCAATTAATCGAGCCTCGTGCGGTAAAGTGTAACGGTTTGTTATTGATTACCACGTATCATTAGCAATAACA

ATCCCCGGGCTCGTATCAAAACCAGTACGACCACTTTGGTTAGGTGTATCGTTTGCAAAGTTGGCTGCTCGTAACGCCTCCGCGGAAACAACCGAATCCTCAAAATTAGACAATTCGTCAAAACCGGGTGGATTTGAGGG

CCCTCAAATCCACCCGGTTTTGACGAATTGTCTAATTTTGAGGATTCGGTTGTTTCCGCGGAGGCGTTACGAGCAGCCAACTTTGCAAACGATACACCTAACCAAAGTGGTCGTACTGGTTTTGATACGAGCCCGGGGAT

TGAAGAAAAACGACTGGAAGTTATAAACATACTTGAGAAAAACCAATGGTCAGTAATACGTTCGTTTGGCTGTTTATCTAATAGTCACCCACTCTTAAAATATAAAACAGCGTTTGA

TCAAACGCTGTTTTATATTTTAAGAGTGGGTGACTATTAGATAAACAGCCAAACGAACGTATTACTGACCATTGGTTTTTCTCAAGTATGTTTATAACTTCCAGTCGTTTTTCTTCA

CATTGAACAAGAGTGCATCGAGGTTGTCCACGCGCGTGTCTATAGTCAAATTCAACTAATGTTGTTCAGAGGGGATGAATCGTTGCGGGTACAATACGTAAACGTCACTATTAATAATCCGTCGATCCAACAAAAAGTACAATGGAGATCG

CCATTGTACTTTTTGTTGGATCGACGGATTATTAATAGTGACGTTTACGTATTGTACCCGCAACGATTCATCCCCTCTGAACAACATTAGTTGAATTTGACTATAGACACGCGCGTGGACAACCTCGATGCACTCTTGTTCAATGAGATCG

CAACTGGCCGTTGCCCCGGTCTCTCGACCTAGAAACATCATAACCGGTGTAGCCGGGACAATTTTCTGACATGCCAACGCTGTGAAAAATACCCGACAGACATCAGTCCATGTATAACCATCATTTATTCCGGGAATAAGAGTTGCGATTT

GACAACAGAAATCGTAAACCTGCCTAAAATCGCAACTCTTATTCCCGGAATAAATGATGGTTATACATGGACTGATGTCTGTCGGGTATTTTTCACAGCGTTGGCATGTCAGAAAATTGTCCCGGCTACACCGGTTATGATGTTTCTAGGT

TCTGGGGTAGAAGGCGTTACTTTTGCACAGAGACTTGCCTGGAAATATATTAGGGAGCTTGTGTTTGCAACCACACTATTCACCTCTGTTTTCCATTGTGGGGAGGTGCGGTTATGTCGCGTTGACCGTCTAGG

CCTAGACGGTCAACGCGACATAACCGCACCTCCCCACAATGGAAAACAGAGGTGAATAGTGTGGTTGCAAACACAAGCTCCCTAATATATTTCCAGGCAAGTCTCTGTGCAAAAGTAACGCCTTCTACCCCAGA

CGCTAACGTTAAATTTTGAGACGGTTAAACGTTTGAATCCATCCGATTATGCCACCTTTACAGTTGGAGGAAAACGTCTTTTTTTTGTGCGCTCTAACGTTCGAGAAAGTCTGCTGGGTGTT

AACACCCAGCAGACTTTCTCGAACGTTAGAGCGCACAAAAAAAAGACGTTTTCCTCCAACTGTAAAGGTGGCATAATCGGATGGATTCAAACGTTTAACCGTCTCAAAATTTAACGTTAGCG

CGACACCGTCTCGCGTGCTGCGGCGGAGGCGTCGTGTGTTTCCATTGCTGAATGGAATAGACGGGCCATGCCGTCTGGGATGGCCGGGTTTGGACGCATAATTGCAGATGCACATCGCCAGATTACATCACCCAAATTGGATATTAATAAG

ATAATATACTGTTAAGTGAGCCAAGCGACGGTTTATGTAGGCGGATGGTGGACGACTAAGCTCGGCCGTCATAACAAACTTATTAATATCCAATTTGGGTGATGTAATCTGGCGATGTGCATCTGCAATTATGCGTCCAAACCCGGCCATC

CGACACCGTCTCGCGTGCTGCGGCGGAGGCGTCGTGTGTTTCCATTGCTGAATGGAATAGACGGGCCATGCCGTCTGGGATGGCCGGGTTTGGACGCATAA

TTATGCGTCCAAACCCGGCCATCCCAGACGGCATGGCCCGTCTATTCCATTCAGCAATGGAAACACACGACGCCTCCGCCGCAGCACGCGAGACGGTGTCG

ATCGGTGGACACATCACAACCGGGGTTTGGCAAGGCCAACCAATCCTTTAACGGGGGGGCAATGTCCGGAACAACAAACGTTCAAAGTGCGTTTAAAACATCGGTGGTCAACAGTATCAACGGCATGCTCGAGGG

CCCTCGAGCATGCCGTTGATACTGTTGACCACCGATGTTTTAAACGCACTTTGAACGTTTGTTGTTCCGGACATTGCCCCCCCGTTAAAGGATTGGTTGGCCTTGCCAAACCCCGGTTGTGATGTGTCCACCGAT

CGCGCCGGGGCCCCGCGACGTATCCCGGCCGTTCAGGGGTCCTGGTGCAGATCCATATTCCATCTTTCCGTTAACATCAACCGATGCCCCGGGTTTTG

CAAAACCCGGGGCATCGGTTGATGTTAACGGAAAGATGGAATATGGATCTGCACCAGGACCCCTGAACGGCCGGGATACGTCGCGGGGCCCCGGCGCG

ATCGGTTGATGTTAACGGAAAGATGGAATATGGATCTGCACCAGGACCCCTGAACGGCCGGGATACGTCGCGGGGCCCCGGCGCGTTTTGTACTCCGGGTTGGG

CCCAACCCGGAGTACAAAACGCGCCGGGGCCCCGCGACGTATCCCGGCCGTTCAGGGGTCCTGGTGCAGATCCATATTCCATCTTTCCGTTAACATCAACCGAT

AGTACAAATTCCCCTCCGGTTGGAACGCCCGACGGGGGCTGTGGTGAGATACCACTAGCGTTACCTGCTACAGAAGCCATATCGCTGTCGTTCCTACACAACTGCGTAACCTTTAAATGCGGAACAGTCTTTTCACAATCTTCATTTGATT

CTGAGGTTTGATACCGGGGGTGCCGCATAGTCCCACGCCTCATATACCGATGACACGCACGGTTCCGTTATAATCAAACTCACATCCGATAGCGG

CCGCTATCGGATGTGAGTTTGATTATAACGGAACCGTGCGTGTCATCGGTATATGAGGCGTGGGACTATGCGGCACCCCCGGTATCAAACCTCAG

GGCCAGGCTCGTTGAGGACATCAACCGTGTTTTTTTATGTATTGCACAGTCGTCGGGACGCGTCACGCGAGATTCACGAAGATTGCGGCGCATATGCCTCGACTTTTAT

ATAAAAGTCGAGGCATATGCGCCGCAATCTTCGTGAATCTCGCGTGACGCGTCCCGACGACTGTGCAATACATAAAAAAACACGGTTGATGTCCTCAACGAGCCTGGCC

ATAGTATCAGAATTAATTTTGGCGCATTATATACACGTGTTAATAGGACCGTCCCTGGGAACGCTCGTGGCCTGTGCTACGTTGGGAACCGCCGCGCACTCGTATATGGACCGATTAT

ATAATCGGTCCATATACGAGTGCGCGGCGGTTCCCAACGTAGCACAGGCCACGAGCGTTCCCAGGGACGGTCCTATTAACACGTGTATATAATGCGCCAAAATTAATTCTGATACTAT

GTCCGGGCATCCAAACACGTAGCAGAACTGCCATGCGTTCTAAATTGTGAGTTGTGGCGAGTACATTTTTATAATTGGTACCAACGAAGACACACCCCTATATCCCTCCACCCATTTCTTTTAAGTCCC

GGGACTTAAAAGAAATGGGTGGAGGGATATAGGGGTGTGTCTTCGTTGGTACCAATTATAAAAATGTACTCGCCACAACTCACAATTTAGAACGCATGGCAGTTCTGCTACGTGTTTGGATGCCCGGAC

CTCCACCCATTTCTTTTAAGTCCCACCCACTAAAACGTGGGTATAAAATGTGTATTGGGGTAGGCGGACAGTCCCAACAAACAGGGAAGTTGAT

ATCAACTTCCCTGTTTGTTGGGACTGTCCGCCTACCCCAATACACATTTTATACCCACGTTTTAGTGGGTGGGACTTAAAAGAAATGGGTGGAG

GTGTGTCTTCGTTGGTACCAATTATAAAAATGTACTCGCCACAACTCACAATTTAGAACGCATGGCAGTTCTGCTACGTGTTTGGATGCCCGGACATTAGAATACAGCCAGTTGTTACCATGGAT

ATCCATGGTAACAACTGGCTGTATTCTAATGTCCGGGCATCCAAACACGTAGCAGAACTGCCATGCGTTCTAAATTGTGAGTTGTGGCGAGTACATTTTTATAATTGGTACCAACGAAGACACAC

CACACGTTGTTAGGCTGCGCTTCTTAAAACCAGTTATGAGATGGGAAAAAGGGCACCCACAAACATACGTATACATTATGTATAT

ATATACATAATGTATACGTATGTTTGTGGGTGCCCTTTTTCCCATCTCATAACTGGTTTTAAGAAGCGCAGCCTAACAACGTGTG

ATATAATATATATAACCTATGTATAATATTGTTTTACATCCTGCATCTTATGTTATATATATTATAT

ATATAATATATATAACATAAGATGCAGGATGTAAAACAATATTATACATAGGTTATATATATTATAT

ATTTTATATATATGATACTGTATATTATTATATACATAATGTATACGTATATTTGTGGGTGCCCTTTTTCACATATCATAACTTGTT

AACAAGTTATGATATGTGAAAAAGGGCACCCACAAATATACGTATACATTATGTATATAATAATATACAGTATCATATATATATAAAAT

CTTAAAACAAGTTATGAGTTGGGAAAAAGGGCACCCACAAACATACGTATAC

GTATACGTATGTTTGTGGGTGCCCTTTTTCCCAACTCATAACTTGTTTTAAG

TATTATTATATACATAATGTATACGTATGTTTGTGGGTGCCCTTTTTCCCAT

ATGGGAAAAAGGGCACCCACAAACATACGTATACATTATGTATATAATAATA
CTATATGTCTTACTGTATAATTATAAAACTTATATATTATCAATTATTATATTTTATACAATAAGACATATAG
CTATATGTCTTATTGTATAAAATATAATAATTGATAATATATAAGTTTTATAATTATACAGTAAGACATATAG
TCCCAAGCCCACACGTTATTAGGCTGCGCTTCTTAAAACAAGTTA
TAACTTGTTTTAAGAAGCGCAGCCTAATAACGTGTGGGCTTGGGA
TATATAATATATATAACCTATGTATAACATTGTTTTACATCCTGCATCTTATGTTATATATATTGTATAAAATATAATAATTGATAA
TTATCAATTATTATATTTTATACAATATATATAACATAAGATGCAGGATGTAAAACAATGTTATACATAGGTTATATATATTATATA
CCAAGCCCACACGTTATTAGGCTGCGCTTCTTAAAA
TTTTAAGAAGCGCAGCCTAATAACGTGTGGGCTTGG
ATTTTATACAATATATATAACATAAGATGCAGGATGTAAAACAATATTATACATAGGTTATATATATTGTATAAAAT
ATTTTATACAATATATATAACCTATGTATAATATTGTTTTACATCCTGCATCTTATGTTATATATATTGTATAAAAT
GTCATATTGTATAAATATAAAACTTATATATTATCAATTATTATATTTTAGACAATATATATAATATAAGATGCAGGATGTAAAACAATATTATACATTGGTTATACA
TGTATAACCAATGTATAATATTGATTTACATCCTGCATCTTATATTATATATATTGTCTAAAATATAATAATTGATAATATATAAGTTTTATATTTATACAAAATGAC
TACTATATGTCTTATTGTATAATTATAAAACTTATATATTATCAATTATTATATTTTAT
ATAAAATATAATAATTGATAATATATAAGTTTTATAATTATACAATAAGACATATAGTA
ATCAATTATTATATTTTATACAATATATATAACATAGGATGCAGGATGTAAAACAATATTATACAT
ATGTATAATATTGTTTTACATCCTGCATCCTATGTTATATATATTGTATAAAATATAATAATTGAT
ATATTTTATACAATATATATAACATAAGATGCAGGATGTAAAACAATATTATGTTATATATATTGTATAAAATAT
ATATTTTATACAATATATATAACATAATATTGTTTTACATCCTGCATCTTATGTTATATATATTGTATAAAATAT

CATATTGTATAATTATAAAACTTAAATATTATCAATTATTATATTTTATACAATATATATAACATAGGAAGCAGGATGTAAAACAATATTATACATAGGTTAT

ATAACCTATGTATAATATTGTTTTACATCCTGCTTCCTATGTTATATATATTGTATAAAATATAATAATTGATAATATTTAAGTTTTATAATTATACAATATG

CAGGATGTAAAACAATATTATACATAGGTTATATATATTATATATATGATACTGTATATTATTATATACATAATGTATACGTATGTTTGTGGGTGCCCTTTTTTCCATCTCATAACTTGTTTT

AAAACAAGTTATGAGATGGAAAAAAGGGCACCCACAAACATACGTATACATTATGTATATAATAATATACAGTATCATATATATAATATATATAACCTATGTATAATATTGTTTTACATCCTG

GTTTTACATCCTGCATCTTATGTTATATATATTGTATAAAATATAATTATTGATAATATATAAGTTTTATAATTATGCTACATGAC

GTCATGTAGCATAATTATAAAACTTATATATTATCAATAATTATATTTTATACAATATATATAACATAAGATGCAGGATGTAAAAC

CAGGTGGGCATGTGACTGAGGCTGGCCAGTGAGAGTATACCATCCTCAAAATTCAAGGTGGCAGCTCACCCCGGCAAGACAAACAGAAGTCCCTGGAGACCTGATTCAGTTGTTCAGGGAGAGAGACAGAGAGTTTGAACAATAGTGAGCC

ACATGCCTTCCTCTATGTGGGCTTCATTCTTAAACAGGCTTCCCCAACGAGATAGTCCACAGAAACCCGAGAACAACTGGCTCACTATTGTTCAAACTCTCTGTCTCTCTCCCTGAACAACTGAATCAGGTCTCCAGGGACTTCTGTTTGT

AGAACCACAGGAGGATCCAGAACAGTTGCCAGATACAAGGTCAGTCTACACAAACCAGTGATACTTCTACCGGCAACCCACCAGAAAGTGTGACTGCTCTTGAGATGGTAACCAGAAAACCCATAAAGTGTTTAGGAATTAATCTAAGAAG

GTGGGGAAAAGAGACATCATTATAACAACAGATCATCCCCTCCATGAGCACGGGATCTTCTTCATTCATCAGACCCACGCCTTCATCCAGGATTAAGTCTGATGGTCTTCTTTCTGGAGGTCTTCTTAGATTAATTCCTAAACACTTTATG

CAGACCCACGCCTTCATCCAGGATTAAGTCTGATGGTCTTCTTTCTGGAGGTCTTCTTAGATTAATTCCTAAACACTTTATGGGTTTTCTGGTTACCATCTCAAGAGCAGTCACACTTTCTGG

CCAGAAAGTGTGACTGCTCTTGAGATGGTAACCAGAAAACCCATAAAGTGTTTAGGAATTAATCTAAGAAGACCTCCAGAAAGAAGACCATCAGACTTAATCCTGGATGAAGGCGTGGGTCTG

CCGTAACATATTTTAACGCATCCCCGTGACCCCCTGTAAACGCACCAGCGTCACAGCGTTCTAGATAAAAAAGCAGTCGCGCCAACAGGGGTGCTCCAAATCCACAAATGAGTGCCAAATAATCCACGCTAAACTCTGTGTTTGATGAACC

TTAAAAGTCCACATTCATACGTTCCGATTCTTGGTGTACCCACAAGGAGACTTGGAGATTTGGTATTGAGCATGTCAAAATTAAAAAACTTAGTTGTAGCTTCTGGCGTTAAATGATCAGGAGTACGGATATCAGCCGGATCTATAAACAT

GTTTATAGATCCGGCTGATATCCGTACTCCTGATCATTTAACGCCAGAAGCTACAACTAAGTTTTTTAATTTTGACATGCTCAATACCAAATCTCCAAGTCTCCTTGTGGGTACACCAAGAATCGGAACGTATGAATGTGGACTTTTAAAG

CCGTAATATTAACGAAAGAACGTCGTCGTCTGATGTGAAAAAGGCCGGTTGTGGTGAAGGACAGTTGGTTTCAGGCTGCCGCCTTTGCGATGTATACTGGTGCGGGGTTGGTGTTGGTGTGGTCTGATCGTCA

TGACGATCAGACCACACCAACACCAACCCCGCACCAGTATACATCGCAAAGGCGGCAGCCTGAAACCAACTGTCCTTCACCACAACCGGCCTTTTTCACATCAGACGACGACGTTCTTTCGTTAATATTACGG

GCAGGGGTTCCCATATCTGAATAAAATGTTTAGCTGGAATTTTCTGGGTAGATCGTAAAAACTTAACTACCTTAACTCTGGCGATTTCTGTTTTCTCTGGTGACCATGGTCTTTC

GAAAGACCATGGTCACCAGAGAAAACAGAAATCGCCAGAGTTAAGGTAGTTAAGTTTTTACGATCTACCCAGAAAATTCCAGCTAAACATTTTATTCAGATATGGGAACCCCTGC

GGACAAGTTGTGGAGGCTGCCAGAAAAACATACGTCGATTTTTTTGAACATGTTTTTGATCGCCGTTATACTCCGGTATACTGGAGTCTTCAGGAGCAAAATTCGGAAACAAAAGCAATACCGGCATCTTATCTGACATACGGACACATGC

GGGGAATACAGGCACACGATGGTAGCAATTCCCAGTAAACAACAGTAGGAGGTCCATGTGGGTTGGGATTACGAACCATAATTATCTGTCTTGGTTTATAATCCTTATCTTGCATGTGTCCGTATGTCAGATAAGATGCCGGTATTGCTTT

GGACAAGTTGTGGAGGCTGCCAGAAAAACATACGTCGATTTTTTTGAACATGTTTTTGATCGCCGTTATACTCCGGTATACTGGAGTCTTCAGGAGCAAAATTCGGAAACAAAAGCAATACCGGCATCTTATCTGACATACGGACACATGC

GGGGAATACAGGCACACGATGGTAGCAACTCCCAGTAAACAACAGTAGGAGGTCCATGTGGGTTGGGATTACGAACCATAATTATCTGTCTTGGTTTATAATCCTTATCTTGCATGTGTCCGTATGTCAGATAAGATGCCGGTATTGCTTT

CGGCAATTAACGTAGCCAGGAGCATTACCGCACCCAAAGTGAACATCATCACCTGAATTATCCAAATTCGCCAATTAAGCGTATCCATTTGATGATCTAACGCTTCCACCTCGGGTGTCGTGGTGTCGTACGGTGAGACTTTTTCAGAACG

TGGGAACTCAAAAGAAGGGGCCGCGTTCTGAAAAAGTCTCACCGTACGACACCACGACACCCGAGGTGGAAGCGTTAGATCATCAAATGGATACGCTTAATTGGCGAATTTGGATAATTCAGGTGATGATGTTCACTTTGGGTGCGGTAAT

TGGGAACTCAAAAGAAGGGGCCGCGTTCTGAAAAAGTCTCACCGTACGACACCACGACACCCGAGGTGGAAGCGTTAGATCATCAAATGGATACGCTTAATTGGCGAATTTGGATAATTCAGGTGATGATGTTCACTTTGGGTGCGGTAAT

CGGCAATTAACGTAGCCAGGAGCATTACCGCACCCAAAGTGAACATCATCACCTGAATTATCCAAATTCGCCAATTAAGCGTATCCATTTGATGATCTAACGCTTCCACCTCGGGTGTCGTGGTGTCGTACGGTGAGACTTTTTCAGAACG

GTGGATGTTCAAAGGGCTGTACGCATATCAATGTCGTTTGAAGTAAATTTGGGACGCCGACGCCCCGATTGTGTTTGTATTATACAAAC

GTTTGTATAATACAAACACAATCGGGGCGTCGGCGTCCCAAATTTACTTCAAACGACATTGATATGCGTACAGCCCTTTGAACATCCAC

CGGACGCGTTCAACCCGAGGCTGGCAAAACTCCTGTCGCCGTTATCCCACGTGGATGTTCAAAGGGCTGTACGCATATCAATGTCGTTTGAAGTAAATTTGGGACGCCGACG

CGTCGGCGTCCCAAATTTACTTCAAACGACATTGATATGCGTACAGCCCTTTGAACATCCACGTGGGATAACGGCGACAGGAGTTTTGCCAGCCTCGGGTTGAACGCGTCCG

GCAGCGGCTGGCTCCGGATTCAACGGACCCGGCGTTTTCATAACCTCCGTTACGGGGGTGTGGTTATGCTTTTTATGCATATTTTCTATGTTTGTTACGGCGGTTGTGTCGGTCTCTCCAAG

CTTGGAGAGACCGACACAACCGCCGTAACAAACATAGAAAATATGCATAAAAAGCATAACCACACCCCCGTAACGGAGGTTATGAAAACGCCGGGTCCGTTGAATCCGGAGCCAGCCGCTGC

GTCAGTCTACGGGTTCATCAAACACAGAGTTTAGCGTGGATTATTTGGCACTCATTTGTGGATTTGGAGCACCCCTGTTGGCGCGACTGCTTTTTTATCTAGAACGCTGTGACGCTGGTGCGTTTACAGGGGGTCACGGGGATGCGTTAAA

GGGGAGGTGCGGTTATGTCGCGTTGACCGTAAAGAGGAATTGGCAAAGTTTATGGGCGGTGTCGTTGGTTCCAACTGGCTTCTCAGTCCATTTAGGGGCTTTTATTGCTTTTCTGGGGTAGAAGGCGTTACTTTTGCACAGAGACTTGCCT

ACGCGACATAACCGCACCTCCCCACAATGGAAAACAGAGGTGAATAGTGTGGTTGCAAACACAAGCTCCCTAATATATTTCCAGGCAAGTCTCTGTGCAAAAGTAACGCCTTCTACCCCAGAAAAGCAATAAAAGCCCCTAAATGGACTGA

ACGCGACATAACCGCACCTCCCCACAATGGAAAACAGAGGTGAATAGTGTGGTTGCAAACACAAGCTCCCTAATATATTTCCAGGCAAGTCTCTGTGCAAAAGTAACGCCTTCTACCCCAGAAAAGCAATAAAAGCCCCTAAATGGACTGA

GGGGAGGTGCGGTTATGTCGCGTTGACCGTAAAGAGGAATTGGCAAAGTTTATGGGCGGTGTCGTTGGTTCCAACTGGCTTCTCAGTCCATTTAGGGGCTTTTATTGCTTTTCTGGGGTAGAAGGCGTTACTTTTGCACAGAGACTTGCCT

Supplemental Table of Normal Values

	**Blood**	
**1**	**White Blood Cell Count**	5-14,000/ uL
**2**	Lymphocytes	20-40%
**3**	Neutrophils	40-60%
**4**	Eosinophils	1-4%
**5**	Hemoglobin	11-15 g/dL
**6**	Erythrocyte Sedimentation Rate (ESR)	<15 mm/hr
**7**	C-Reactive Protein (CRP)	<1.0 mg/mL
**8**	Aspartate Aminotransferase (AST)	15-40 units/L
**9**	Alanine Aminotransferase (ALT)	10-25 units/L
	**Cerebrospinal Fluid**	
**10**	Protein	16-31 mg/dL
**11**	Glucose	50-80 mg/dL
**12**	Cell Count	<8 cells/mm^3
**13**	IL-1RA	<10.8 pg/mL
**14**	IL-6	<2.0 pg/mL
**15**	IL-8	<6.0 pg/mL
**16**	IL-10	<2.0 pg/mL
**17**	IL-17F	<2.0 pg/mL
**18**	IFNg	<6.8 pg/mL
**19**	CXCL10	<67.8 pg/mL
**20**	CCL2	<73.3 pg/mL
**21**	GCSF	<17.3 pg/mL
**22**	CXCL9	<3.0 pg/mL

Note: Normal values taken from the Nelson Textbook of Pediatrics, 20th Edition. Editors: R.M. Kliegman, J.W. St.Geme, N.F. Schor: ELSEVER, 2015; and the Human Immunology and Immunotherapy Programs at the Washington University Immunomonitoring Laboratory, St. Louis, MO. Normal values for some cytokines in the cerebrospinal fluids of children are not yet established. .
